# Synthesis of highly condensed phospholes by the Lewis acid-assisted dehydrogenative Mallory reaction under visible light irradiation†

**DOI:** 10.1039/d4sc05657d

**Published:** 2024-11-14

**Authors:** Ikki Kamiyoshi, Yuki Kojima, Shibo Xu, Kosuke Yasui, Yuji Nishii, Koji Hirano

**Affiliations:** a Department of Applied Chemistry, Graduate School of Engineering, Osaka University Suita Osaka 565-0871 Japan k_hirano@chem.eng.osaka-u.ac.jp; b Innovative Catalysis Science Division, Institute for Open and Transdisciplinary Research Initiatives (ICS-OTRI), Osaka University Suita Osaka 565-0871 Japan

## Abstract

A photo-promoted oxidative cyclization, that is, the Mallory reaction of 2,3-diarylbenzophopholes has been developed. With the assistance of Bi(OTf)_3_ Lewis acid, the reaction proceeds smoothly under visible light irradiation even without any external oxidants. The newly developed dehydrogenative conditions are compatible with various functional groups and substitution patterns, which enables the streamlined synthesis of highly condensed dibenzophosphole derivatives of potent interest in material chemistry. Moreover, experimental and computational studies unveil the detailed reaction mechanism. The preliminary optoelectronic properties of some newly synthesized compounds are also demonstrated.

## Abstract 2

A Bi(OTf)_3_-assisted, visible-light-promoted dehydrogenative cyclization of 2,3-diarylbenzophospholes proceeds to form the corresponding highly condensed dibenzophospholes of interest in material chemistry.

## Graphical abstract

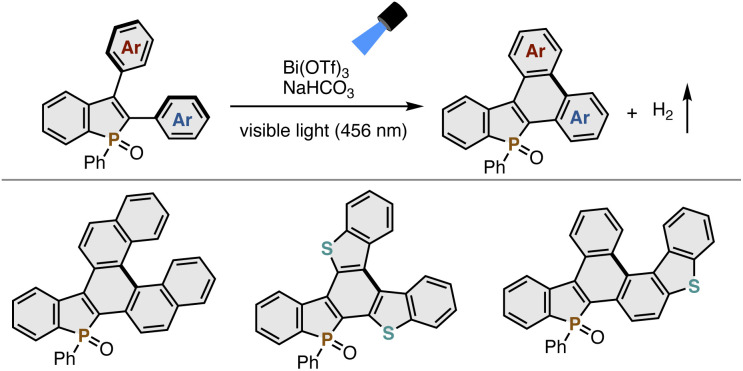

## Introduction

**Scheme 1:**
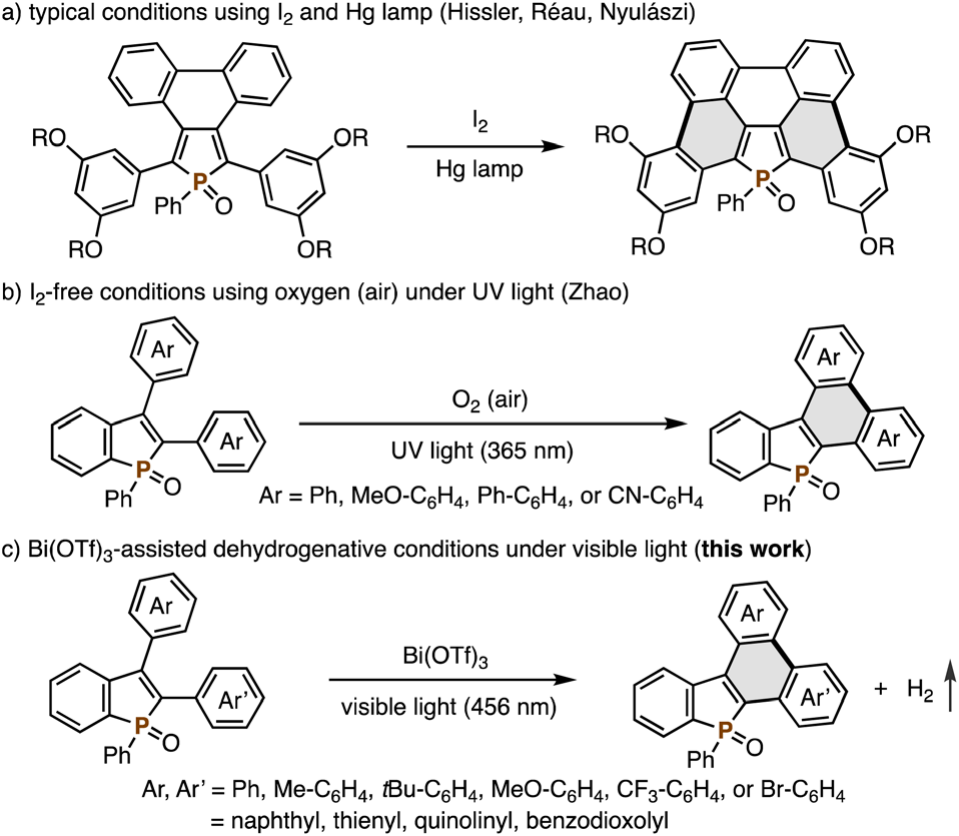
Mallory reactions for synthesis of highly condensed phosphole derivatives: (a) typical conditions using a mercury (Hg) vapour lamp and I_2_, (b) I_2_-free conditions using UV light and O_2_, and (c) Bi(OTf)_3_-assisted dehydrogenative conditions under visible light.

Highly condensed heteroaromatics are key structures in organic functional materials. In particular, phosphorus-incorporated phosphole derivatives have received significant attention because of their unique physical and optoelectronic properties in the design and synthesis of organic light-emitting diodes (OLEDs), organic photovoltaics (OPVs), and cell imaging dyes.^1^ Among various reported synthetic approaches to the aforementioned important heteroaromatic core,^2^ the photo-promoted oxidative cyclization reaction, namely, the Mallory reaction is one of the most promising candidates.^3^ Hissler, Réau, Nyulászi, and co-workers reported successful synthesis of highly condensed dibenzophospholes by the Mallory reaction of peripherally arylated phospholes (Scheme 1a).^4^ However, a high-pressure mercury vapour lamp and I_2_ external oxidant were necessary to promote the reaction. Additionally, the substituents on the phosphole nuclei are limited to strongly electron-donating dialkoxyphenyl or thienyl groups. Very recently, Zhao developed the reaction of 2,3-diarylbenzophospholes under I_2_-free conditions using molecular oxygen as the oxidant, but still UV light (365 nm) was required (Scheme 1b).^5^

On the other hand, in recent years our research group serendipitously found that 3-indolylphosphole underwent dehydrogenative cyclization even under visible light irradiation.^6^ This phenomenon was specific to a phosphole bearing the strongly electron-donating indole substituent at the C3 position, suggesting that the substrate scope can be extended by suitable electronic perturbation with the assistance of external additives. Herein, we report the significant substrate extension: the Bi(OTf)_3_-assisted Mallory reaction of 2,3-diarylbenzophospholes under blue LED irradiation (456 nm) has been developed (Scheme 1c). The reaction conditions accommodate electron-donating and electron-withdrawing aryl groups as well as heteroaryl groups on the phosphole core. In addition, mild reaction conditions with visible light of lower energy are tolerant of a wide range of functional groups such as the Ar–Br moiety, which can be further transformed. Moreover, even in the absence of any external oxidants, the reaction proceeds smoothly with concomitant evolution of molecular hydrogen. Experimental and computational mechanistic studies and preliminary optoelectronic properties of some newly synthesized condensed phosphole derivatives are also described.

## Results and discussion

We began optimization studies with 2,3-diphenylbenzophosphole oxide 1a (0.050 mmol) to identify suitable additives under blue LED irradiation (456 nm, 40 W, N_2_ conditions, at 40–50 °C under light irradiation) in MeCN solvent (Table 1). After careful investigation, several Lewis acidic metal triflates were found to promote the reaction: Bi(OTf)_3_, Al(OTf)_3_, In(OTf)_3_, and Sc(OTf)_3_ provided the desired 2a in 33–68% yields (entries 1–4). In addition, some Brønsted acids, TFA and (PhO)_2_P(O)(OH), also showed activity (entries 5 and 6), while stronger acids including TfOH, PTSA, and HCl, gave a complicated mixture and weaker AcOH resulted in no conversion (entries 7–10). Given the detrimental effects of TfOH (entry 7), we next tested the addition of bases to quench trace amounts of TfOH potentially generated *in situ* from metal triflates. Gratifyingly, full conversion of 1a and isolation of 2a in an almost quantitative yield were achieved by using a combination of Bi(OTf)_3_ and NaHCO_3_ (entry 11), whereas more basic additives such as Na_2_CO_3_ totally shut down the reaction (entry 12). In contrast, NaHCO_3_ had a negative impact when combined with other metal triflates (entries 13–15). Although details still remain unclear, Lewis basic NaHCO_3_ can decrease the Lewis acidity of metal triflates other than Bi(OTf)_3_. Thus, the combination is crucial for satisfactory conversion of 1a. Some control experiments were also performed: no reaction was observed in the dark even at elevated temperature (50 °C; entry 16). In the absence of additives, the starting 1a was recovered completely (entry 17). These results clearly indicate the indispensable role of both light and Bi(OTf)_3_ Lewis acid in the reaction (see the ESI† for more detailed optimization studies, including additional screening of acid additives and solvent effects).

**Table 1:** Condition optimization for the dehydrogenative Mallory reaction of 1a under visible light irradiation^a^

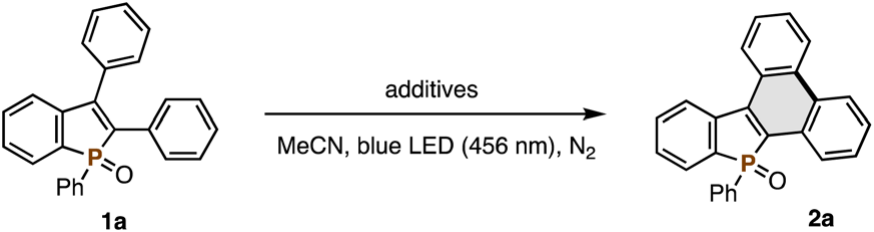		
Entry	Additives	Yield of 2a^b^ (%)
1	Bi(OTf)_3_	33
2	Al(OTf)_3_	49
3	In(OTf)_3_	68
4	Sc(OTf)_3_	34
5	TFA	40
6	(PhO)_2_P(O)(OH)	20
7	TfOH	0
8	PTSA	0
9	HCl	0
10	AcOH	0
11	Bi(OTf)_3_, NaHCO_3_	(>99)
12	Bi(OTf)_3_, Na_2_CO_3_	<5
13	Al(OTf)_3_, NaHCO_3_	38
14	In(OTf)_3_, NaHCO_3_	31
15	Sc(OTf)_3_, NaHCO_3_	8
16^c^	Bi(OTf)_3_, NaHCO_3_	0
17	None	0

a Conditions: 1a (0.050 mmol), additives (0.050 mmol), MeCN (1.0 mL), blue LED (456 nm, 40 W), ambient temperature (40–50 °C under light irradiation), 22 h, N_2_.

b Estimated by using ^31^P{^1^H} NMR based on 0.050 mmol with P(O)(OEt)_3_ as the internal standard. Isolated yield is in parentheses.

c In the dark at 50 °C. Tf = trifluoromethanesulfonyl, TFA = trifluoroacetic acid, PTSA = *p*-toluenesulfonic acid, and Ac = acetyl.

**Scheme 2:**
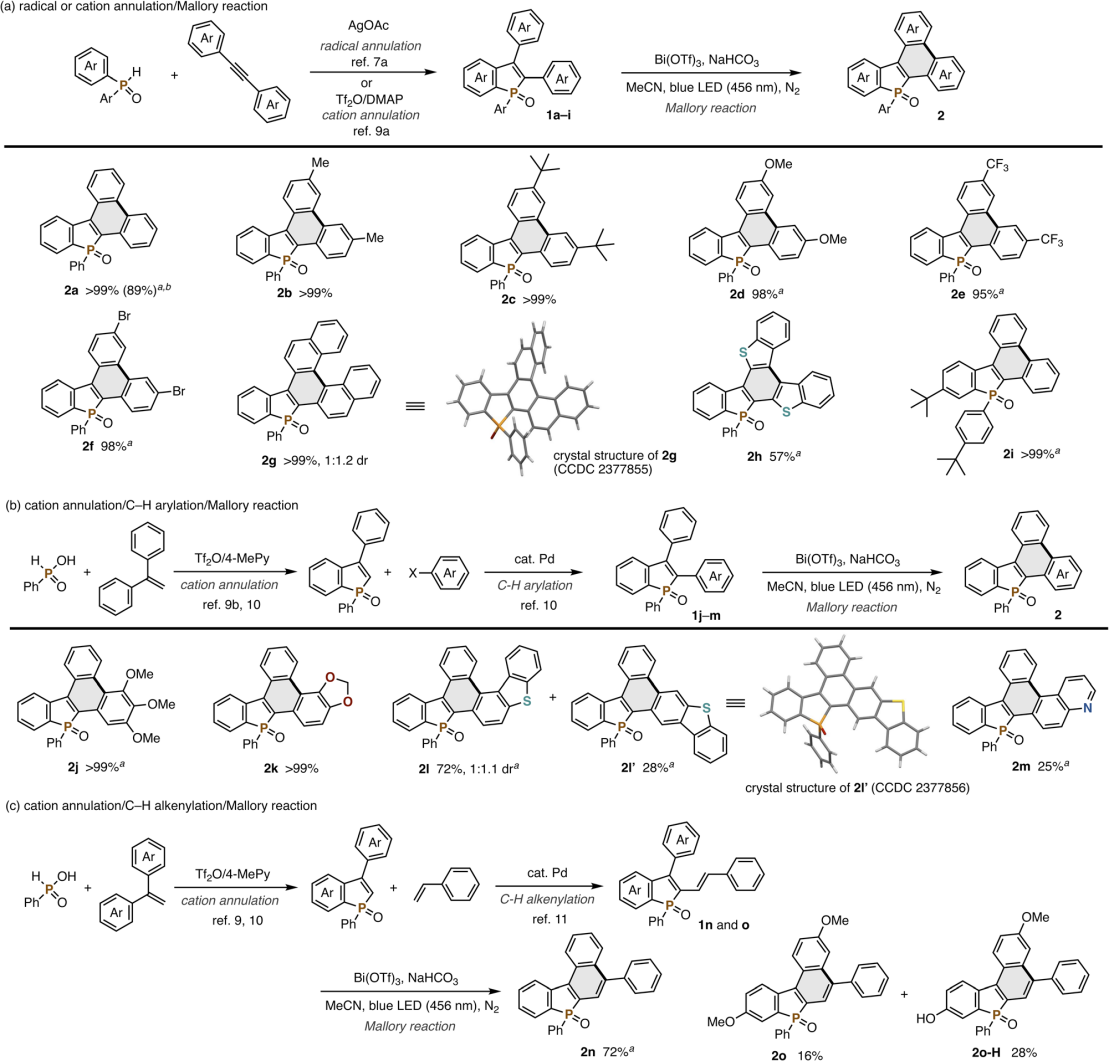
Products of the Bi(OTf)_3_-assisted dehydrogenative Mallory reaction of phosphole derivatives 1. Isolated yields are shown. Conditions of the Mallory reaction: 1 (0.030–0.11 mmol), Bi(OTf)_3_ (1.0 equiv.), NaHCO_3_ (1.0 equiv.), MeCN (1.0 mL), blue LED (456 nm, 40 W), ambient temperature (40–50 °C under light irradiation), 22 h, N_2_. ^*a*^With two LEDs (40 W × 2). ^*b*^On a 1.0 mmol scale.

**Scheme 3:**
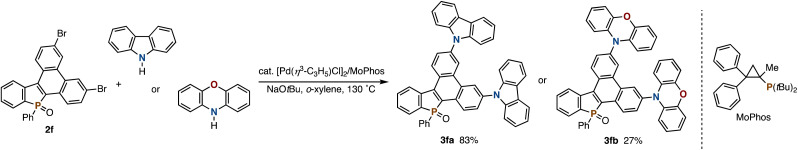
Double Buchwald–Hartwig amination of dibrominated benzophosphole 2f with carbazole and phenoxazine. See the ESI† for detailed reaction conditions.

Combined with our previously developed convergent synthetic methods of 2,3-disubstituted benzophospholes, the visible-light-promoted Mallory reaction can offer a modular approach to the highly condensed dibenzophospholes with structural diversity. One is the radical annulation–Mallory reaction sequence (Scheme 2a): a variety of 2,3-diarylbenzophospholes 1a–h readily prepared from diphenylphosphine oxide and symmetrical diaryl alkynes^7^ underwent the Mallory reaction smoothly (Scheme 2a). Electron-neutral (2b and 2c), -rich (2d), and -deficient (2e) substrates were all tolerated. Owing to the visible light of relatively lower energy, the reaction conditions were compatible with the Ar–Br bond (2f), which is a versatile handle in downstream transformation. Actually, the Pd-catalysed double Buchwald–Hartwig amination of 2f with carbazole and phenoxazine^8^ enabled concise synthesis of the dibenzophosphole-based donor–acceptor systems 3fa and 3fb in 83% and 27% yield, respectively (Scheme 3). In the case of 2-naphthyl-substituted benzophosphole 1g, dehydrogenative cyclization occurred regioselectively to deliver the helical 2g in an almost quantitative yield. Its structure was unambiguously confirmed by X-ray analysis (CCDC 2377855).† The heteroaromatic benzothiophene substituent could also be employed, and the corresponding P,S-doped triphenylene 2h was obtained in an acceptable yield. Combination with the phosphorus-cation-mediated annulation of the *t*-Bu-substituted diphenyl phosphine oxide^9*a*^ successfully provided dibenzophosphole 2i with the *t*-Bu-C_6_H_4_ substituent on the phosphorus, indicating that the diverse substitution pattern is accessible. The reaction also proceeded on a 1.0 mmol scale with synthetically useful efficacy (2a).

The phosphorus-dication-mediated annulation^9*b*^ and successive Pd-catalysed C–H arylation^10^ provided facile access to the benzophospholes 1j–m with two different aromatic rings at the C2 and C3 positions, which were also amenable substrates in the Mallory reaction (Scheme 2b). The highly electron-donating trimethoxyphenyl (2j) and methylenedioxyphenyl (2k) groups at the C2 position were well tolerated under specific reaction conditions. Notably, exclusive regioselectivity was observed in the latter case. On the other hand, the dibenzothiophene-substituted substrate 1l afforded a regioisomeric mixture of helical 2l and linear 2l′ (CCDC 2377856).† The Lewis basic quinoline-fused dibenzophosphole 2m was also obtained albeit with a moderate yield.

The Mallory reaction was also applicable to the C2-alkenyl-C3-arylbenzophospholes 1n and 1o, which were also easily synthesized *via* Pd-catalysed C–H alkenylation^11^ (Scheme 2c). In the case of 1n, we successfully obtained the 5,7-diphenyldibenzo[*b*,*e*]phosphindole 7-oxide structure 2n, which is employed as the OLED device for improvement of emission efficiency.^12^ With the MeO-substituted substrate 1o, the partial demethylation of the MeO group competitively occurred, but a good combined yield of 2o and 2o-H was observed.

**Scheme 4:**
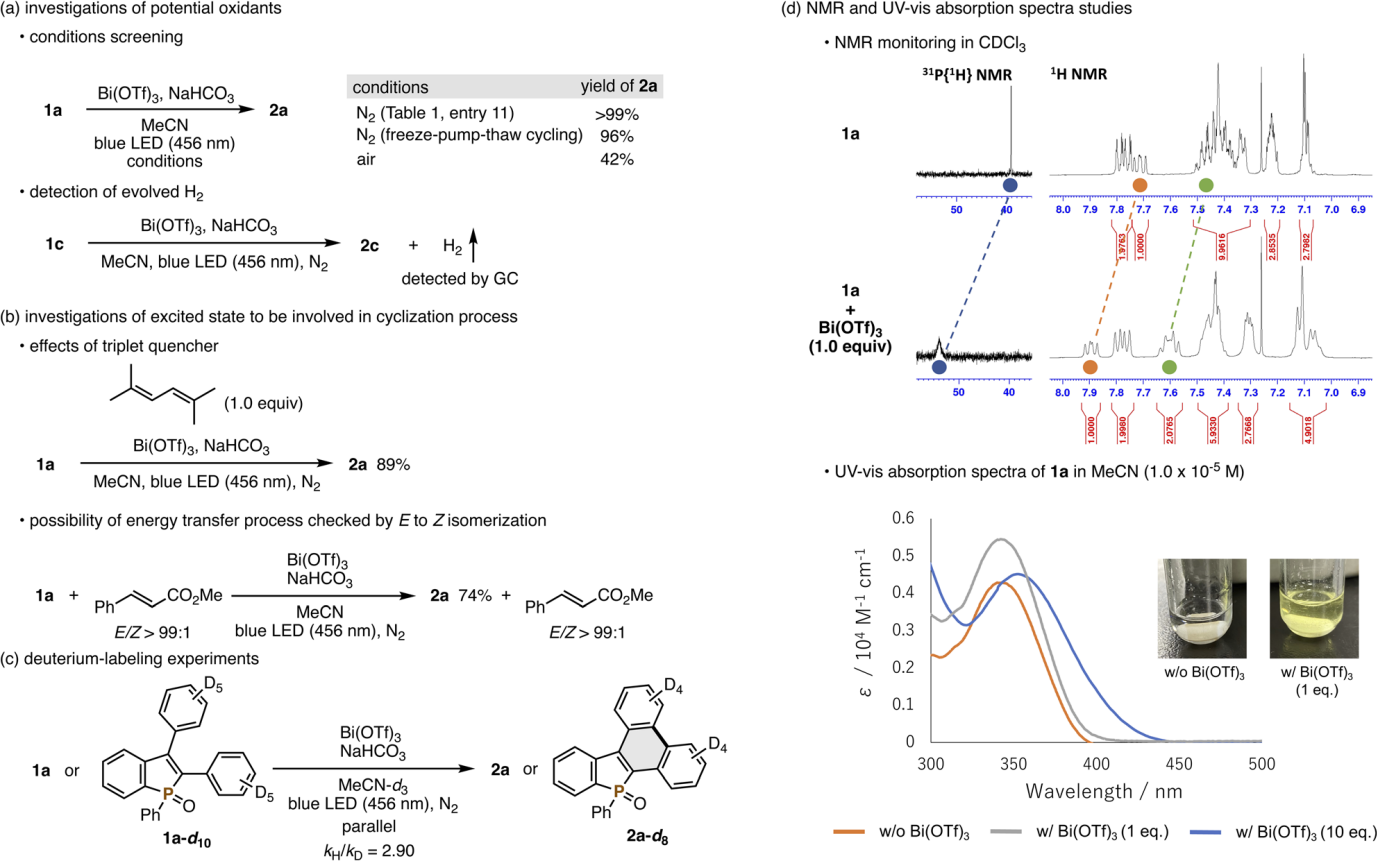
Experimental mechanistic studies.

To gain insight into the reaction mechanism, several experiments were performed next. First, we investigated potential oxidants in the present Mallory reaction (Scheme 4a). Zhao recently reported that residual oxygen in solvent is the actual external oxidant in the related UV-light-promoted Mallory reaction, which was supported by no conversion under rigorously deaerated conditions using freeze–pump–thaw cycling.^5^ Thus, our Bi(OTf)_3_-assisted, visible-light-promoted Mallory reaction was also conducted by using the same deaeration technique. Surprisingly, the reaction proceeded smoothly without any difficulty, and 2a was formed in 96% yield comparable with that under the standard nitrogen conditions (>99% yield, Table 1, entry 11). In addition, a lower yield was observed in air. These results suggest that oxygen in air unlikely works as the external oxidant. Finally, we successfully observed evolution of molecular hydrogen by GC after the reaction, thus indicating that the present Mallory reaction is acceptor-less, and a truly dehydrogenative reaction. To check whether the reaction is operated in the singlet or triplet excited state, we then carried out the reaction of 1a in the presence of 2,5-dimethyl-2,4-hexadiene, which is known as a triplet quencher,^13^ but 2a was still formed in a good yield (Scheme 4b). Even with (*E*)-methyl cinnamate, which undergoes *E* to *Z* isomerization by triplet–triplet energy transfer,^14^1a was converted to 2a efficiently, and cinnamate was recovered with the maintenance of its (*E*)-configuration. Although the possibility of a triplet state intermediacy cannot be completely excluded, given the effective quenching even in the case of intramolecular cyclization,^15^ the aforementioned outcomes support the reaction progress *via* the singlet excited state rather than the triplet excited state. Information about the rate-determining step was obtained from the deuterium-labeling experiment with 1a-*d*_10_ (Scheme 4c): the kinetic isotope effect (KIE) value from the parallel reaction was 2.90, thus suggesting the rate-limiting C–H cleavage. Finally, the role of Bi(OTf)_3_ was examined by using NMR and UV-vis absorption spectra (Scheme 4). Upon mixing of 1a and Bi(OTf)_3_ in CDCl_3_, some ^1^H and ^31^P{^1^H} NMR signals were shifted to the lower field. The absorption spectrum was also red-shifted, upon exposure of 1a to Bi(OTf)_3_, to the visible light region. The colour change in the solution could also be confirmed visually. Apparently, the complexation between the phosphine oxide moiety of 1a and Bi(OTf)_3_ enables excitation even with visible light.

**Scheme 5:**
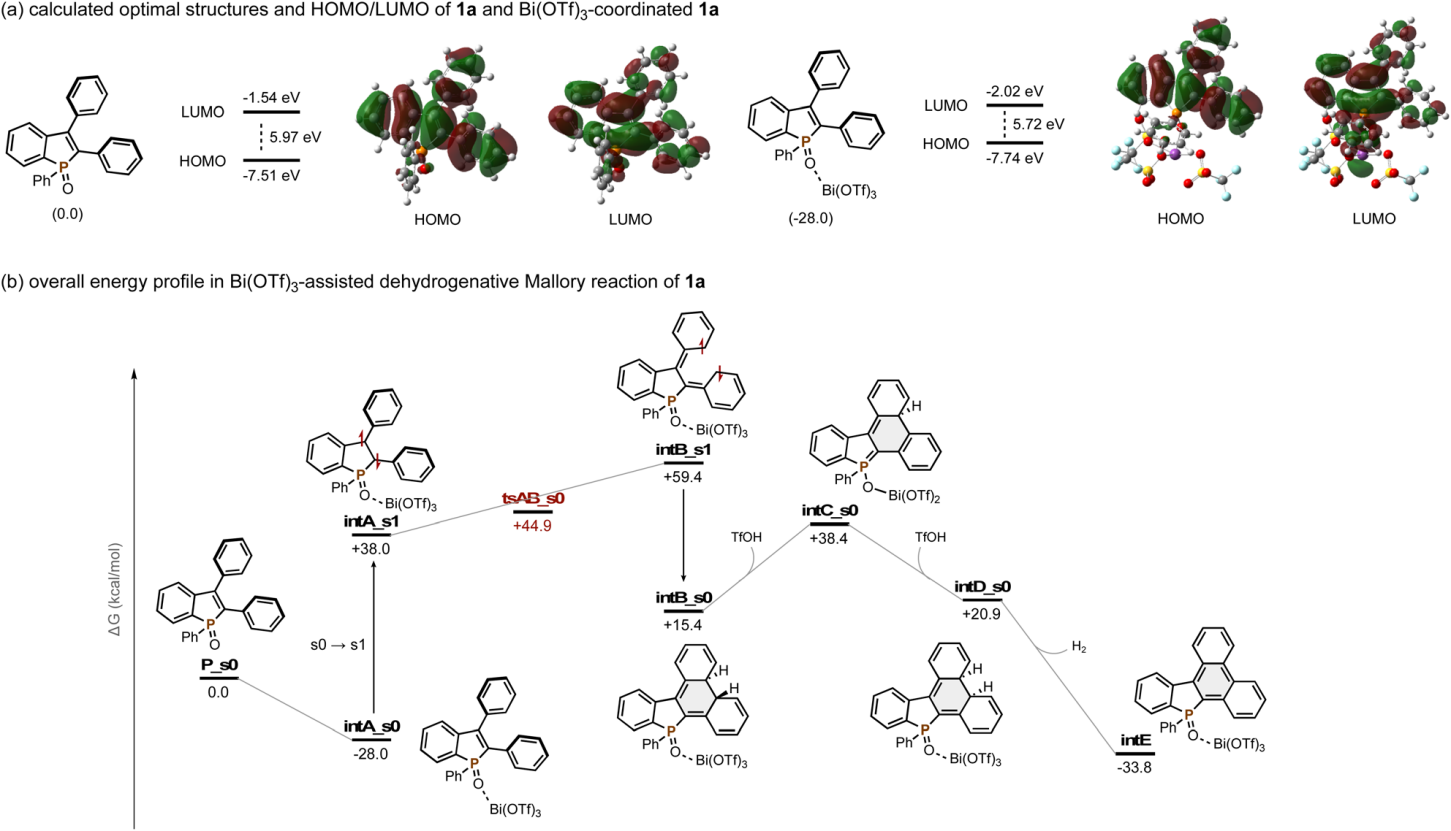
Computational mechanistic studies. Summary of the level of theory: M06-2X/6-311+G(d,p)&SDD/PCM(MeCN)//M06-2X/6-31G(d)&LanL2DZ.

Additional computational mechanistic studies were performed by using density functional theory (DFT) calculations (Scheme 5). All calculations were carried out using the Gaussian 16 program.^16^ The singlet ground-state (S_0_) and singlet exited-state (S_1_) geometries were optimized by the DFT and time-dependent density functional theory (TD-DFT) methods with the M06-2X functional and a standard 6-31G(d) basis set (LanL2DZ basis set for Bi). The M06-2X functional is a high-nonlocality functional with double the amount of nonlocal exchange (2X), with reliable performance for thermochemistry, hydrogen bonding, kinetics, and weak interactions.^17^ The optimized molecular structures were verified by vibrational analysis; equilibrium structures did not have imaginary frequencies and transition state structures had only one imaginary frequency. The intrinsic reaction coordinate (IRC) calculations were carried out to check the transition state leading to the reactant and the product. Single-point energies were calculated using the 6-311+G(d,p) basis set (SDD basis set for Bi), and the solvent effect of MeCN was taken into account by using the integral equation formalism PCM (IEF-PCM). We first optimized the molecular structures of 1a and Bi(OTf)_3_-coordinated 1a in the S_0_ state and calculated their HOMO and LUMO levels (Scheme 5a). Upon the coordination of Bi(OTf)_3_, the smaller HOMO–LUMO gap is obtained particularly by lowering the LUMO level, which is in good agreement with the NMR and UV-vis absorption spectral changes observed in Scheme 4d.^18^ In addition, the Bi(OTf)_3_ coordination is an energetically favoured, exothermic process (Δ*G* = −28.0 kcal mol^−1^). Thus, Bi(OTf)_3_-coordinated 1a (intA_s0) is a rational starting point in the present Mallory reaction (Scheme 5b). Consistent with no conversion under dark conditions (Table 1, entry 16), the activation barrier for direct electrocyclization in the ground state (tsAB_s0) is +72.9 kcal mol^−1^, which can exclude the thermal reaction pathway. On the other hand, the S_1_ intermediate intA_s1 undergoes conrotatory electrocyclization to form intB_s0*via*intB_s1 with a reasonable energy change (+38.0 to +59.4 to +15.4 kcal mol^−1^). The direct elimination of *vic*-hydrogens in intB_s0 is difficult because they are *anti* to each other. Thus, enolization-induced epimerization occurs through intC_s0 to afford the corresponding *syn*-isomer intD_s0. The process is uphill, but subsequent spontaneous elimination of molecular hydrogen into the gas phase and rearomatization into intE are strong driving forces for the reaction process. The overall energy profile reveals that the rate-limiting step is the C–H cleaved enolization from intB_s0 to intC_s0, which is also in accordance with the primary KIE value observed in Scheme 4c.

**Fig. 1:**
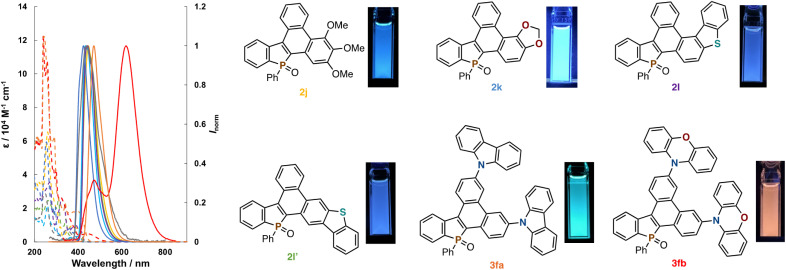
UV-vis absorption (dashed lines) and fluorescence spectra (solid lines) of 1a, 2a, 2j, 2k, 2l, 2l′, 3fa, and 3fb in CHCl_3_ (1.0 × 10^−5^ M), and fluorescence images of 2j, 2k, 2l, 2l′, 3fa, and 3fb.

The optical properties of some newly obtained compounds in Schemes 2 and 3 (2j, 2k, 2l, 2l′, 3fa, and 3fb) were preliminary surveyed in a solution state (1.0 × 10^−5^ M CHCl_3_ solution). The data of absorption/emission properties (*λ*_abs_/*λ*_em_) and fluorescence quantum yields (*Φ*_F_), also involving those of 1a and 2a as the references, are summarized in Fig. 1 and Table 2. Compared to the uncyclized 1a, the condensed 2a–2l′ all showed bathochromic shifts of their *λ*_abs_ values (379–412 nm) probably because of effective π-extension. On the other hand, impacts on the emission maxima *λ*_em_ were little or negligible. Relatively high quantum yields were observed in the push–pull-type alkoxy-substituted 2j and 2k, while higher fused 2l and 2l′ containing the dibenzothiophene core showed lower emission efficiency. In general, the smaller Stokes shifts were obtained from all compounds owing to their rigid planar structures. The carbazole-incorporated dibenzophosphole 3fa exhibited both absorption and emission maxima in the longer wavelength regions, which is reflected by the strongly electron-donating nature of the carbazole unit. Additionally, a very high quantum yield was obtained. The introduction of phenoxazine induced much larger red shifts of *λ*_abs_ and *λ*_em_ albeit with poor emission efficiency (3fb).

**Table 2:** Optical properties of 1a, 2a, 2j, 2k, 2l, 2l′, 3fa, and 3fb^a^

Compd	*λ*_abs_ (nm)	*λ*_em_ b (nm)	*Φ*_F_ c	Δ*ν*^d^/cm^−1^
1a	249, 342	438	0.11	6409
2a^e^	254, 315, 364, 382	425	0.56	2649
2j	260, 379	448	0.58	4064
2k	260, 403	434	0.75	1772
2l	212, 270, 324, 392	443	0.16	2937
2l′	262, 310, 344, 391, 412	432	0.16	1124
3fa	244, 291, 341, 396	473	>0.99	4111
3fb	240, 258, 298, 317, 440	620	0.04	6598

a Measured in 1.0 × 10^−5^ M solution of CHCl_3_.

b Excited at 1a (249 nm), 2a (254 nm), 2j (260 nm), 2k (260 nm), 2l (270 nm), 2l′ (262 nm), 3fa (244 nm), and 3fb (298 nm).

c Absolute fluorescence quantum yields.

d Stokes shifts.

e The optical data of 2a were taken from ref. 10.

We also examined the electrochemical properties by cyclic voltammetry (CV) and differential pulse voltammetry (DPV) in MeCN with tetrabutylammonium hexafluorophosphate (Bu_4_NPF_6_) as an electrolyte *versus* ferrocene/ferrocenium ions (Fc/Fc^+^) (Fig. S14–S19†), and their HOMO and LUMO levels were estimated according to the first oxidation potentials and the optical band gaps (*E*^opt^_g_). The data are summarized in Table 3. The CV of all compounds showed irreversible oxidation waves, and thus *E*_ox_ values were determined by DPV. The electron-donating alkoxy (2j and 2k) and amino (3fa and 3fb) substituents largely shifted *E*_ox_ values in a negative direction from those of the parent 2a. Notably, compared to the carbazole-substituted 3fa, the phenoxazine-substituted 3fb exhibited a higher-lying HOMO level but an almost identical LUMO level, thus suggesting its larger intramolecular charge transfer ability. On the other hand, the structural isomers 2l and 2l′ showed almost the same values for all parameters: their electrochemical properties are less dependent on the orientation of fused dibenzothiophene.

**Table 3:** Absorption wavelengths, HOMO–LUMO energy gaps and differential pulse voltammetry data of compounds 2a, 2j, 2k, 2l, 2l′, 3fa, and 3fb

Compd	λ^abs^_onset_^a^ (nm)	*E* ^opt^_g_ b (eV)	*E*_ox_ c (V)	*E*_HOMO_ d (eV)	*E*_LUMO_ e (eV)
2a^f^	397	3.12	1.32	−6.12	−3.00
2j	422	2.94	0.91	−5.71	−2.77
2k	424	2.92	0.99	−5.79	−2.87
2l	429	2.89	1.08	−5.88	−2.99
2l′	428	2.90	1.07	−5.87	−2.97
3fa	445	2.79	0.72	−5.52	−2.73
3fb	521	2.38	0.32	−5.12	−2.74

a Measured in CHCl_3_.

b Determined from the onset of the absorption spectra.

c Performed in MeCN in the presence of Bu_4_NPF_6_. *v* = 0.10 V s^−1^. Values determined by DPV, *versus* Fc/Fc^+^.

d The approximation for the Fc/Fc^+^ level is −4.8 eV *versus* vacuum: *E*_HOMO_ = −4.8 − *E*_ox_.

e Estimated from *E*_HOMO_ and *E*^opt^_g_: *E*_LUMO_ = *E*_HOMO_ + *E*^opt^_g_.

f The data of 2a were taken from ref. 10.

## Conclusions

We have revisited the classical Mallory reaction of 2,3-diarylbenzophosphole derivatives and developed visible-light-promoted conditions with the assistance of Bi(OTf)_3_ Lewis acid. The use of visible light with lower energy enables the concise synthesis of highly condensed dibenzophospholes bearing a variety of functional groups, which are of potent interest in organic material chemistry. Additionally noteworthy is the evolution of molecular hydrogen: the reaction proceeds even under external oxidant-free conditions and is thus truly dehydrogenative.^19^ Moreover, several experimental and computational studies uncover the detailed reaction mechanism. Development of catalytic conditions and application to other (hetero)aromatic systems as well as preparation of more condensed helically chiral molecules are ongoing in our laboratory.

## Data availability

All experimental procedures and spectroscopic data can be found in the ESI.†

## Author contributions

K. H. conceived the idea. I. K. and S. X. performed all experiments. Y. K. conducted computational studies with DFT. K. Y. and Y. N. assisted with X-ray analysis. K. H. supervised the project and wrote the manuscript. All the authors discussed the results and commented on the manuscript.

## Conflicts of interest

There are no conflicts to declare.

## Supplementary Material

SC-015-D4SC05657D-s001

SC-015-D4SC05657D-s002

## References

[cit1] (d) Baumgartner T. and Jäkle F., Main Group Strategies towards Functional Hybrid Materials, Wiley, 2018. Selected examples:

[cit2] Wu B., Yoshikai N. (2016). Org. Biomol. Chem..

[cit3] Mallory F. B., Mallory C. W. (1984). Org. React..

[cit4] Fadhel O., Szieberth D., Deborde V., Lescop C., Nyulászi L., Hissler M., Réau R. (2009). Chem.–Eur. J..

[cit5] Li J., Zhuang Z., Guo J., Dong X., Gong J., Tang B. Z., Zhao Z. (2023). Adv. Sci..

[cit6] Nishimura K., Xu S., Nishii Y., Hirano K. (2023). Org. Lett..

[cit7] Unoh Y., Hirano K., Satoh T., Miura M. (2013). Angew. Chem., Int. Ed..

[cit8] Suzuki K., Hori Y., Kobayashi T. (2008). Adv. Synth. Catal..

[cit9] Nishimura K., Unoh Y., Hirano K., Miura M. (2018). Chem.–Eur. J..

[cit10] Xu S., Nishimura K., Saito K., Hirano K., Miura M. (2022). Chem. Sci..

[cit11] Tokura Y., Xu S., Kojima Y., Miura M., Hirano K. (2022). Chem. Commun..

[cit12] Kang M. Y. ,, Hong S. G., Ha J. S., Lee D. H., Park T. Y., Jang B. J. and Seo S. D., Organic light emitting device comprising light efficiency improvement layer comprising novel dibenzo compounds, KR2016027940A, 2016-03-10

[cit13] Zhu M., Huang X.-L., Sun S., Zhenga C., You S.-L. (2021). J. Am. Chem. Soc..

[cit14] Nevesely T., Wienhold M., Molloy J. J., Gilmour R. (2022). Chem. Rev..

[cit15] Zhang S.-Z., Zhang S.-S., Li J.-L., Shen S., Yang X.-L., Niu X. (2023). J. Org. Chem..

[cit16] Frisch M. J. , Trucks G. W., Schlegel H. B., Scuseria G. E., Robb M. A., Cheeseman J. R., Scalmani G., Barone V., Petersson G. A., Nakatsuji H., Li X., Caricato M., Marenich A. V., Bloino J., Janesko B. G., Gomperts R., Mennucci B., Hratchian H. P., Ortiz J. V., Izmaylov A. F., Sonnenberg J. L., Williams-Young D., Ding F., Lipparini F., Egidi F., Goings J., Peng B., Petrone A., Henderson T., Ranasinghe D., Zakrzewski V. G., Gao J., Rega N., Zheng G., Liang W., Hada M., Ehara M., Toyota K., Fukuda R., Hasegawa J., Ishida M., Nakajima T., Honda Y., Kitao O., Nakai H., Vreven T., Throssell K., Montgomery Jr J. A., Peralta J. E., Ogliaro F., Bearpark M. J., Heyd J. J., Brothers E. N., Kudin K. N., Staroverov V. N., Keith T. A., Kobayashi R., Normand J., Raghavachari K., Rendell A. P., Burant J. C., Iyengar S. S., Tomasi J., Cossi M., Millam J. M., Klene M., Adamo C., Cammi R., Ochterski J. W., Martin R. L., Morokuma K., Farkas O., Foresman J. B. and Fox D. J., Gaussian 16, Revision B.01., Gaussian, Inc., Wallingford CT, 2016

[cit17] Zhao Y., Truhlar D. G. (2008). Theor. Chem. Acc..

[cit18] We also confirmed that the S_0_ to S_1_ transition arises from the HOMO–LUMO transition by TD-DFT calculation. Oscillator strengths of 1a and Bi(OTf)_3_-coordinated 1a are 0.2727 and 0.1982, respectively. See the ESI† for more details.

[cit19] Wang H., Gao X., Lv Z., Abdelilah T., Lei A. (2019). Chem. Rev..

