# Supplementary material for: Synthesis of highly condensed phospholes by the Lewis acid-assisted dehydrogenative Mallory reaction under visible light irradiation
Source: Chem Sci. 2024 Nov 14;15(48):20413–20. doi: 10.1039/d4sc05657d (PMC11580199; doi:10.1039/d4sc05657d)
Supplement: SC-015-D4SC05657D-s001 [file SC-015-D4SC05657D-s001.pdf]

Supplementary Information (SI) for Chemical Science.
This journal is © The Royal Society of Chemistry 2024

Supplementary Information

Synthesis of highly condensed phospholes by the Lewis acid-assisted dehydrogenative Mallory reaction under visible light irradiation

Ikki Kamiyoshi,† Yuki Kojima,† Shibo Xu,‡ Kosuke Yasui,†,‡ Yuji Nishii,†,‡ and Koji Hirano*,†,‡

†*Department of Applied Chemistry, Graduate School of Engineering, Osaka University, Suita, Osaka 565-0871, Japan*

‡*Innovative Catalysis Science Division, Institute for Open and Transdisciplinary Research Initiatives (ICS-OTRI), Osaka University, Suita, Osaka 565-0871, Japan*

E-mail: k_hirano@chem.eng.osaka-u.ac.jp (K.H.)

Contents

**Instrumentation and Chemicals**	S2–S3
**Experimental Procedures and Characterization Data for Products**	S4–S19
**X-Ray Analysis**	S20–S21
**Detailed Optimization Studies**	S22–S23
**Unsuccessful Substrates**	S24
**Detection of Evolved H_2**	S25
**Deuterium-Labeling Experiments**	S26–S28
**NMR Studies**	S29
**UV-vis Absorption Spectra Studies**	S30
**Computational Studies**	S31–S54
**Photoluminescence Properties**	S55–S56
**Electrochemical Properties**	S57–S60
**Copies of NMR spectra**	S61–S98
**References**	S99

Instrumentation and Chemicals

^{1}H, ^{13}C{^{1}H}, ^{19}F{^{1}H} and ^{31}P{^{1}H} NMR spectra were recorded at 400 MHz, 100 MHz, 376 MHz, and 162 MHz respectively, for $CDCl_3$ or DMSO-d_6 solutions. HRMS data were obtained by APCI using TOF. GC analysis was carried out using a silicon OV-17 column (i. d. 2.6 mm x 1.5 m) or a CBP-1 capillary column (i. d. 0.5 mm x 25 m). TLC analyses were performed on commercial glass plates bearing a 0.25 mm layer of Merck silica gel $60F_{254}$. Silica gel (60 N, spherical neutral, Kanto Chemical Co.) was used for column chromatography. Gel permeation chromatography (GPC) was performed by LC-20AR (pump, SHIMADZU, 7.5 mL/min $CHCl_3$) and SPD-20A (UV detector, SHIMADZU, 254 nm) with two in-line YMC-GPC T2000 (20 x 600 mm, particle size: 10 μm) (preparative columns, YMC). LED irradiation was performed by Kessil KSPR160L (456 nm, 40 W) at ambient temperature. UV-vis spectra were acquired with JASCO V-750 spectrometer. Photoluminescence spectra and quantum yield measurements were conducted with JASCO FP-8500 spectrometer equipped with an integration sphere system. The crystal measurement was performed with XtaLAB Synergy-S/Cu (Rigaku). Cyclic voltammograms and differential pulse voltammograms were recorded on ALS Electrochemical Analyzer Model 600E equipped with SVC-3 Voltammetry cell. Counter and working electrodes were made of Pt, and the reference electrode was Ag/Ag^{+}. The

working electrodes were polished on a cloth polishing pad in an alumina slurry and then washed in H_2O under sonication before use. The measurements were conducted in MeCN solvent (degassed by N_2 gas bubbling) containing tetrabutylammonium hexafluorophosphate as a supporting electrolyte at an indicated scan rate. All the potentials were calibrated with the standard ferrocene/ferrocenium (Fc/Fc^+) redox couple measured in identical conditions.

Unless otherwise noted, materials obtained from commercial suppliers were used without further purification. $Bi(OTf)_3$ was purchased from Thermo Fischer Scientific. The benzophospholes **1a**–**h**,[S1] **1i**,[S2] **1j**–**m**,[S3] and **1n**–**o**[S4] were prepared according to the literature. Unless otherwise noted, all reactions were performed under nitrogen atmosphere.

Experimental Procedures and Characterization Data for Products

Bi(OTf)$_3$-assisted dehydrogenative Mallory reaction of benzophospholes: General Procedure A

The benzophosphole oxide **1** (1.0 equiv) and $NaHCO_3$ (1.0 equiv) were placed in a 2–5 mL (for 0.030–0.11 mmol scale reactions) or 10–20 mL (for 1.0 mmol scale reactions) microwave vial (Biotage). The vial was introduced into a nitrogen-filled glove box, and Bi(OTf)$_3$ (1.0 equiv) and MeCN were added. The vial was capped with an aluminum cap and taken out from the glove box. The resulting mixture was stirred under blue LED irradiation at ambient temperature (40–50 ˚C by the light irradiation) (one or two Kessil KSPR160L, 456 nm, 40 W, see Figure S1). After 22 h, Et_3N (3.0 mL) was added to scavenge Bi(OTf)$_3$ from the phosphole. The reaction mixture was filtered through a short pad of silica gel (60 N, spherical neutral) and then concentrated in vacuo. The residue was purified by column chromatography on silica gel (60 N, spherical neutral) and/or GPC to give the corresponding condensed dibenzophosphole oxide **2**.

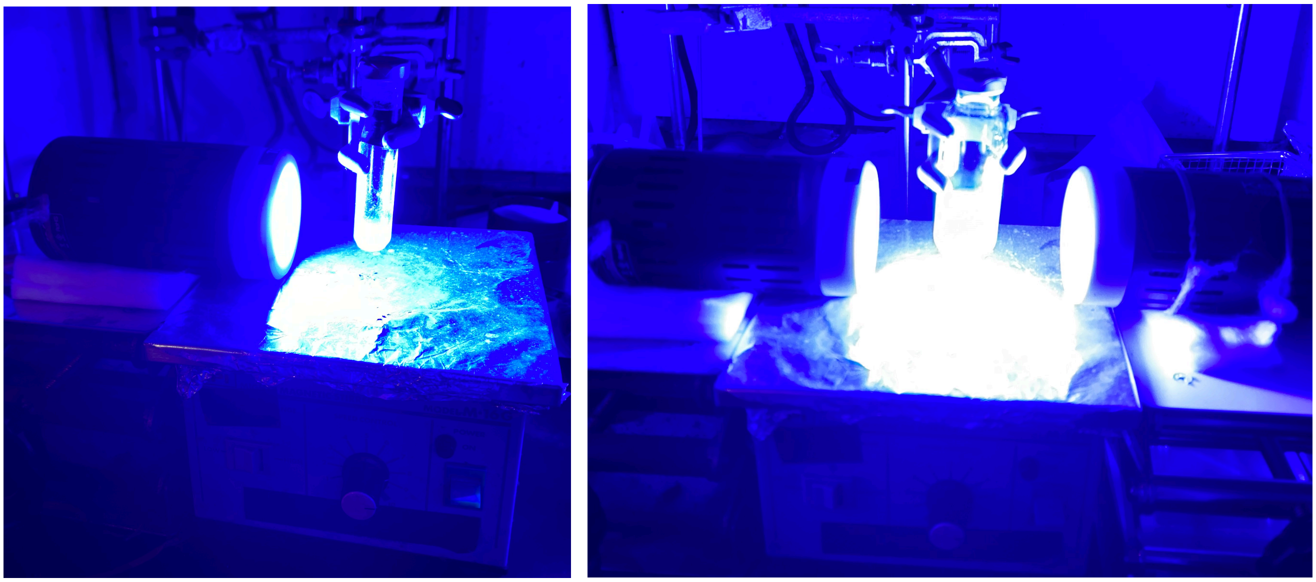

Figure S1. Pictures for reaction set-up with Kessil KSPR160L (456 nm, 40 W). The distance between the light source and reaction vessel was 2.0 cm. left: with one light source, right: with two light sources.

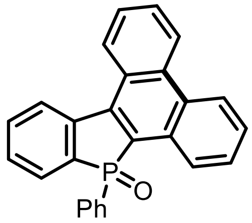

9-Phenyltribenzo[*b,e,g*]phosphindole 9-oxide (2a)

On a 0.050 mmol scale, synthesized from **1a** (19 mg, 0.050 mmol, 1.0 equiv), $NaHCO_3$ (4.2 mg, 0.050 mmol, 1.0 equiv), $Bi(OTf)_3$ (33 mg, 0.050 mmol, 1.0 equiv), and MeCN (1.0 mL), with one Kessil KSPR160L lamp, according to **General Procedure A**, purified by silica gel (60 N, spherical neutral) column chromatography with hexane/EtOAc (1/1, v/v) and GPC ($CHCl_3$): 19 mg (quant, 0.050 mmol scale); on a 1.0 mmol scale, synthesized from **1a** (380 mg, 1.0 mmol, 1.0 equiv), $NaHCO_3$ (360 mg, 1.0 mmol, 1.0 equiv), $Bi(OTf)_3$ (660 mg, 0.050 mmol, 1.0 equiv), and MeCN (20 mL), with two Kessil KSPR160L lamps, according to **General Procedure A**, purified by silica gel (60 N, spherical neutral) column chromatography with hexane/EtOAc (1/1, v/v) and GPC ($CHCl_3$): 324 mg (89%, 1.0 mmol scale); white solid; m.p. 174.5-175.5 °C; ^{1}H NMR (400 MHz, $CDCl_3$) δ 8.99-8.97 (m, 1H), 8.82-8.78 (m, 1H), 8.36 (d, J = 8.4 Hz, 1H), 8.53 (dd, J = 8.0, 3.5 Hz, 1H), 8.31 (d, J = 8.0 Hz, 1H), 7.86-7.72 (m, 5H), 7.69-7.62 (m, 2H), 7.57-7.53 (m, 1H), 7.47-7.42 (m, 2H), 7.37-7.33 (m, 2H); ^{13}C{^{1}H} NMR (100 MHz, $CDCl_3$) δ 142.5 (d, J = 23.2 Hz, 1C), 139.5 (d, J = 20.0 Hz, 1C), 134.6 (d, J = 105.8 Hz, 1C), 133.9 (d, J = 1.8 Hz, 1C), 133.1 (d, J = 1.9 Hz, 1C), 132.2 (d, J = 2.8 Hz, 1C), 131.1 (d, J = 10.9 Hz, 2C), 130.8 (d, J = 102.0 Hz, 1C), 130.7 (d, J = 8.2 Hz, 1C), 130.1 (d, J = 9.7 Hz, 1C), 130.0 (d, J = 102.4 Hz, 1C), 129.3 (d, J = 8.8 Hz, 1C), 128.94 (d, J = 11.1 Hz, 1C), 128.88 (d, J = 12.4 Hz, 2C), 128.6 (1C), 128.0 (1C), 127.99 (d, J = 13.2 Hz, 1C), 127.8 (1C), 127.4 (1C), 127.0 (d, J = 5.4 Hz, 1C), 125.8 (1C), 125.6 (d, J = 10.9 Hz, 1C), 124.1 (1C), 123.0 (1C); ^{31}P{^{1}H} NMR (162 MHz, $CDCl_3$) δ 34.1; HRMS (APCI) m/z ([M+H]$^+$) calcd for $C_{26}H_{18}OP$: 377.1090, found: 377.1093.

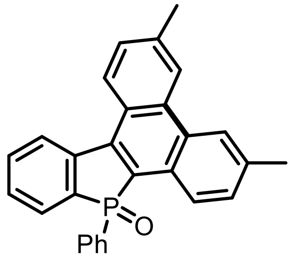

3,6-Dimethyl-9-phenyltribenzo[*b,e,g*]phosphindole 9-oxide (2b)

Synthesized from **1b** (20 mg, 0.050 mmol, 1.0 equiv), $NaHCO_3$ (4.2 mg, 0.050 mmol, 1.0 equiv),

$Bi(OTf)_3$ (33 mg, 0.050 mmol, 1.0 equiv), and MeCN (1.0 mL), with one Kessil KSPR160L lamp, according to **General Procedure A**, purified by silica gel (60 N, spherical neutral) column chromatography with hexane/EtOAc (1/1, v/v) and GPC ($CHCl_3$): 21 mg (quant, 0.050 mmol scale); yellow solid; m.p. 254.7-256.4 °C; ^{1}H NMR (400 MHz, $CDCl_3$) δ 8.83 (d, J = 8.6 Hz, 1H), 8.57 (s, 1H), 8.47 (dd, J = 8.0, 3.5 Hz, 1H), 8.43 (s, 1H), 8.17 (d, J = 8.2 Hz, 1H), 7.83-7.78 (m, 1H), 7.75-7.69 (m, 2H), 7.66-7.61 (m, 1H), 7.58-7.56 (m, 1H), 7.45-7.30 (m, 5H), 2.66 (s, 3H), 2.56 (s, 3H); ^{13}C{^{1}H} NMR (100 MHz, $CDCl_3$) δ 142.8 (d, J = 23.4 Hz, 1C), 138.5 (d, J = 19.6 Hz, 1C), 138.6 (1C), 137.6 (1C), 134.6 (d, J = 105.7 Hz, 1C), 133.8 (d, J = 1.6 Hz, 1C), 133.0 (d, J = 1.9 Hz, 1C), 132.0 (d, J = 2.8 Hz, 1C), 131.1 (d, J = 10.8 Hz, 2C), 131.0 (d, J = 115.1 Hz, 1C), 130.6 (d, J = 4.8 Hz, 1C), 130.0 (d, J = 9.6 Hz, 1C), 129.6 (1C), 128.9 (1C), 128.84 (d, J = 103.0 Hz, 1C), 128.80 (d, J = 12.4 Hz, 2C), 128.6 (d, J = 11.2 Hz, 1C), 127.4 (d, J = 8.9 Hz, 1C), 126.8 (d, J = 5.5 Hz, 1C), 126.1 (d, J = 12.1 Hz, 1C), 125.5 (1C), 125.4 (d, J = 10.6 Hz, 1C), 123.8 (1C), 122.8 (1C), 22.2 (1C), 22.1 (1C); ^{31}P{^{1}H} NMR (162 MHz, $CDCl_3$) δ 34.0; HRMS (APCI) m/z ([M+H]$^+$) calcd for $C_{28}H_{22}OP$: 405.1403, found: 405.1420.

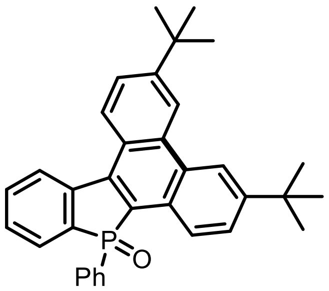

3,6-Di-*tert*-butyl-9-phenyltribenzo[*b,e,g*]phosphindole 9-oxide (2c)

Synthesized from **1c** (25 mg, 0.050 mmol, 1.0 equiv), $NaHCO_3$ (4.2 mg, 0.050 mmol, 1.0 equiv), $Bi(OTf)_3$ (33 mg, 0.050 mmol, 1.0 equiv), and MeCN (1.0 mL), with one Kessil KSPR160L lamp, according to **General Procedure A**, purified by silica gel (60 N, spherical neutral) column chromatography with hexane/EtOAc (1/1, v/v) and GPC ($CHCl_3$): 25 mg (quant, 0.050 mmol scale); yellow solid; m.p. 137.8-140.1 °C; ^{1}H NMR (400 MHz, $CDCl_3$) δ 8.92 (d, J = 8.9 Hz, 1H), 8.83-8.82 (m, 1H), 8.67 (s, 1H), 8.52 (dd, J = 8.0, 3.4 Hz, 1H), 8.22 (d, J = 8.6 Hz, 1H), 7.86-7.72 (m, 4H), 7.66-7.62 (m, 2H), 7.45-7.31 (m, 4H), 1.55 (s, 9H), 1.46 (s, 9H); ^{13}C{^{1}H} NMR (100 MHz, $CDCl_3$) δ 151.2 (1C), 150.6 (1C), 142.8 (d, J = 23.4 Hz, 1C), 138.6 (d, J = 19.8 Hz, 1C), 134.7 (d, J = 105.6 Hz, 1C), 133.9 (1C), 133.0 (1C), 132.0 (d, J = 2.5 Hz, 1C), 131.1 (d, J = 10.8 Hz, 2C), 131.0 (d, J = 111.4 Hz, 1C), 130.6 (1C), 130.0 (d, J = 8.9 Hz, 1C), 128.9 (d, J = 103.0 Hz, 1C), 128.8 (d, J = 12.4 Hz, 2C),

128.6 (1C), 128.5 (1C), 127.4 (d, J = 9.2 Hz, 1C), 126.7 (d, J = 5.4 Hz, 1C), 126.4 (1C), 126.1 (d, J = 12.1 Hz, 1C), 125.6 (d, J = 5.5 Hz, 1C), 125.4 (d, J = 10.7 Hz, 1C), 119.4 (1C), 118.4 (1C), 35.4 (2C), 31.3 (6C); $^{31}P\{^1H\}$ NMR (162 MHz, $CDCl_3$) δ 34.1; HRMS (APCI) m/z ($[M+H]^+$) calcd for $C_{34}H_{34}OP$: 489.2342, found: 489.2342.

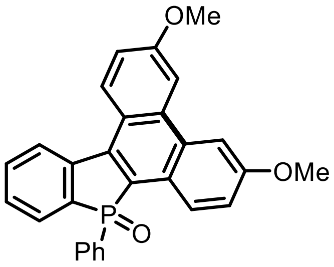

3,6-Dimethoxy-9-phenyltribenzo[*b,e,g*]phosphindole 9-oxide (2d)

Synthesized from **1d** (22 mg, 0.050 mmol, 1.0 equiv), $NaHCO_3$ (4.2 mg, 0.050 mmol, 1.0 equiv), $Bi(OTf)_3$ (33 mg, 0.050 mmol, 1.0 equiv), and MeCN (1.0 mL), with two Kessil KSPR160L lamps, according to **General Procedure A**, purified by silica gel (60 N, spherical neutral) column chromatography with hexane/EtOAc (1/1, v/v) and GPC ($CHCl_3$): 21.6 mg (98%, 0.050 mmol scale); yellow solid; m.p. 208.0-211.3 °C; ^1H NMR (400 MHz, $CDCl_3$) δ 8.84 (d, J = 9.3 Hz, 1H), 8.40 (dd, J = 8.0, 3.5 Hz, 1H), 8.18 (d, J = 8.9 Hz, 1H), 8.00 (d, J = 2.6 Hz, 1H), 7.89-7.88 (m, 1H), 7.78-7.69 (m, 3H), 7.64-7.59 (m, 1H), 7.46-7.32 (m, 5H), 7.16 (dd, J = 8.9, 2.5 Hz, 1H), 4.03 (s, 3H), 3.95 (s, 3H); $^{13}C\{^1H\}$ NMR (100 MHz, $CDCl_3$) δ 159.1 (d, J = 29.0 Hz, 1C), 142.8 (d, J = 23.4 Hz, 1C), 137.1 (d, J = 20.1 Hz, 1C), 135.2 (d, J = 1.9 Hz, 1C), 134.5 (d, J = 106.0 Hz, 1C), 133.0 (d, J = 1.8 Hz, 1C), 132.0 (d, J = 2.7 Hz, 1C), 131.7 (d, J = 8.7 Hz, 1C), 131.2 (d, J = 101.8 Hz, 1C), 131.1 (d, J = 10.8 Hz, 2C), 129.9 (d, J = 9.9 Hz, 1C), 128.8 (d, J = 12.4 Hz, 2C), 128.5 (1C), 128.4 (1C), 128.3 (1C), 127.4 (1C), 127.0 (d, J = 104.2 Hz, 1C), 125.0 (d, J = 10.7 Hz, 1C), 124.4 (d, J = 9.0 Hz, 1C), 122.8 (d, J = 12.3 Hz, 1C), 117.7 (1C), 116.9 (1C), 105.8 (1C), 105.2 (1C), 55.54 (1C), 55.52 (1C); $^{31}P\{^1H\}$ NMR (162 MHz, $CDCl_3$) δ 34.1; HRMS (APCI) m/z ($[M+H]^+$) calcd for $C_{28}H_{22}O_3P$: 437.1301, found: 437.1313.

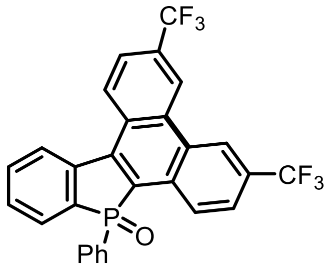

9-Phenyl-3,6-bis(trifluoromethyl)tribenzo[*b,e,g*]phosphindole 9-oxide (2e)

Synthesized from **1e** (26 mg, 0.050 mmol, 1.0 equiv), $NaHCO_3$ (4.2 mg, 0.050 mmol, 1.0 equiv), $Bi(OTf)_3$ (33 mg, 0.050 mmol, 1.0 equiv), and MeCN (1.0 mL), with two Kessil KSPR160L lamps, according to **General Procedure A**, purified by silica gel (60 N, spherical neutral) column chromatography with hexane/EtOAc (1/1, v/v) and GPC ($CHCl_3$): 24.3 mg (95%, 0.050 mmol scale); white solid; m.p. 238.3-240.0 °C; ^{1}H NMR (400 MHz, $CDCl_3$) δ 9.14 (d, J = 8.8 Hz, 1H), 9.06 (s, 1H), 8.93 (s, 1H), 8.52 (dd, J = 8.0, 3.5 Hz, 1H), 8.47 (d, J = 8.4 Hz, 1H), 8.06-8.04 (m, 1H), 7.90-7.69 (m, 5H), 7.56-7.48 (m, 2H), 7.41-7.37 (m, 2H); ^{13}C{^{1}H} NMR (100 MHz, $CDCl_3$) δ 141.4, 141.2, 140.9, 140.7, 134.9, 133.8, 133.59, 133.58, 133.3, 132.71, 132.68, 132.5, 132.0, 131.9, 131.5, 131.4, 131.3, 131.0, 130.93, 130.87, 130.6, 130.54, 130.50, 130.18, 130.16, 130.0, 129.94, 129.90, 129.8, 129.5, 129.20, 129.16, 129.07, 128.02, 127.96, 127.0, 125.9, 125.8, 125.4, 125.3, 124.9, 124.8, 124.20, 124.17, 122.7, 122.6, 121.40, 121.36 (All signals cannot be completely assigned because of complexity associated with C–F coupling and C–P coupling. Thus, the observed signals were simply drawn.); ^{31}P{^{1}H} NMR (162 MHz, $CDCl_3$) δ 33.3; ^{19}F{^{1}H} NMR (376 MHz, $CDCl_3$) δ -62.2, -62.4; HRMS (APCI) m/z ($[M+H]^+$) calcd for $C_{28}H_{16}F_6OP$: 513.0837, found: 513.0851.

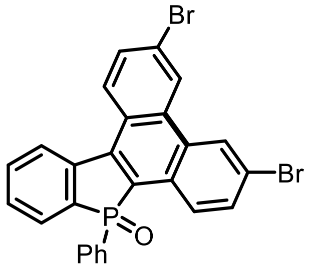

3,6-Dibromo-9-phenyltribenzo[*b,e,g*]phosphindole 9-oxide (2f)

Synthesized from **1f** (26 mg, 0.050 mmol, 1.0 equiv), $NaHCO_3$ (4.2 mg, 0.050 mmol, 1.0 equiv), $Bi(OTf)_3$ (33 mg, 0.050 mmol, 1.0 equiv), and MeCN (1.0 mL), with two Kessil KSPR160L lamps, according to **General Procedure A**, purified by silica gel (60 N, spherical neutral) column

chromatography with hexane/EtOAc (1/1, v/v) and GPC ($CHCl_3$): 24.9 mg (93%, 0.050 mmol scale); white solid; m.p. 271.7-273.6 °C; ^{1}H NMR (400 MHz, $CDCl_3$) δ 8.81-8.79 (m, 2H), 8.68 (s, 1H), 8.43-8.41 (m, 1H), 8.16 (d, *J* = 8.6 Hz, 1H), 7.87-7.81 (m, 2H), 7.72-7.64 (m, 4H), 7.48-7.45 (m, 2H), 7.39-7.34 (m, 2H); ^{13}C{^{1}H} NMR (100 MHz, $CDCl_3$) δ 141.6 (d, *J* = 22.8 Hz, 1C), 139.3 (d, *J* = 20.0 Hz, 1C), 134.3 (d, *J* = 106.2 Hz, 1C), 134.1 (d, *J* = 1.5 Hz, 1C), 133.4 (d, *J* = 1.8 Hz, 1C), 132.4 (d, *J* = 2.8 Hz, 1C), 131.8 (1C), 131.2 (1C), 131.0 (d, *J* = 10.8 Hz, 2C), 130.8 (d, *J* = 8.4 Hz, 1C), 130.23 (d, *J* = 9.8 Hz, 1C), 130.22 (d, *J* = 102.2 Hz, 1C), 130.1 (d, *J* = 101.6 Hz, 1C), 129.3 (d, *J* = 11.4 Hz, 1C), 129.0 (d, *J* = 12.5 Hz, 2C), 128.3 (d, *J* = 5.3 Hz, 1C), 128.1 (d, *J* = 9.1 Hz, 1C), 127.8 (1C), 126.8 (1C), 126.7 (d, *J* = 12.1 Hz, 1C), 126.0 (1C), 125.5 (d, *J* = 10.6 Hz, 1C), 123.7 (1C), 122.8 (1C); ^{31}P{^{1}H} NMR (162 MHz, $CDCl_3$) δ 33.4; HRMS (APCI) m/z ($[M+H]^+$) calcd for $C_{26}H_{16}Br_2OP$: 532.9300, found: 532.9280.

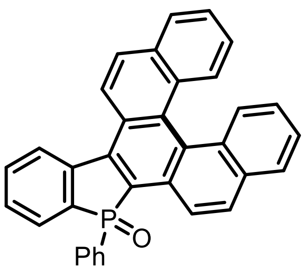

A 1:1.16 Diastereomixture of 11-phenylbenzo[*b*]dinaphtho[2,1-*e*:1',2'-*g*]phosphindole 11-oxide (2g)

Synthesized from **1g** (24 mg, 0.050 mmol, 1.0 equiv), $NaHCO_3$ (4.2 mg, 0.050 mmol, 1.0 equiv), $Bi(OTf)_3$ (33 mg, 0.050 mmol, 1.0 equiv), and MeCN (1.0 mL), with one Kessil KSPR160L lamp, according to **General Procedure A**, purified by silica gel (60 N, spherical neutral) column chromatography with hexane/EtOAc (1/1, v/v) and GPC ($CHCl_3$): 24 mg (quant, 0.050 mmol scale); yellow solid; m.p. 243.0-246.0 °C; ^{1}H NMR (400 MHz, $CDCl_3$) δ 8.90 (t, *J* = 9.7 Hz, 1H), 8.56 (dd, *J* = 8.0, 3.4 Hz, 1H), 8.38-8.34 (m, 1H), 8.28-8.25 (m, 1H), 8.16-8.08 (m, 1H), 8.00 (d, *J* = 8.2 Hz, 1H), 7.94-7.68 (m, 7H), 7.60-7.56 (m, 1H), 7.52-7.41 (m, 4H), 7.39-7.32 (m, 2H), 7.27-7.20 (m, 1H); ^{13}C{^{1}H} NMR (100 MHz, $CDCl_3$) δ 142.7, 142.5, 142.4, 142.3, 139.2, 139.0, 138.9, 138.8, 135.1, 134.8, 134.6, 134.1, 133.3, 132.8, 132.6, 132.5, 132.4, 132.3, 132.2, 131.5, 131.2, 131.1, 131.0, 130.9, 130.6, 130.5, 130.43, 130.36, 130.26, 130.2, 130.1, 129.5, 129.2, 129.12, 129.07, 128.99, 128.9, 128.8, 128.5, 128.4, 128.1, 128.0, 127.7, 127.6, 127.4, 127.2, 127.1, 126.9, 126.9, 125.84, 125.79, 125.74, 125.68, 125.1, 125.0, 124.9, 123.6, 123.5, 122.92, 122.86, 121.8, 121.7 (All signals cannot be

completely assigned because of complexity associated with diastereomers and C–P coupling. Thus, the observed signals were simply drawn.); $^{31}P\{^{1}H\}$ NMR (162 MHz, $CDCl_3$) δ 33.6, 32.8; HRMS (APCI) m/z ($[M+H]^+$) calcd for $C_{34}H_{22}OP$: 477.1403, found: 477.1417.

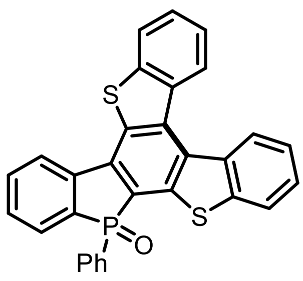

2h

Synthesized from **1h** (15 mg, 0.030 mmol, 1.0 equiv), $NaHCO_3$ (2.5 mg, 0.030 mmol, 1.0 equiv), $Bi(OTf)_3$ (20 mg, 0.030 mmol, 1.0 equiv), and MeCN (0.60 mL), with two Kessil KSPR160L lamps, according to **General Procedure A**, purified by silica gel (60 N, spherical neutral) column chromatography with hexane/EtOAc (1/1, v/v) and GPC ($CHCl_3$): 8.4 mg (57%, 0.030 mmol scale); yellow solid; m.p. 259.7-261.4 °C; ^{1}H NMR (400 MHz, $CDCl_3$) δ 9.04 (d, J = 8.1 Hz, 1H), 8.95-8.93 (m, 1H), 8.37 (dd, J = 7.9, 3.4 Hz, 1H), 8.08-8.06 (m, 1H), 7.92-7.85 (m, 2H), 7.81-7.76 (m, 3H), 7.63-7.48 (m, 6H), 7.42-7.38 (m, 2H); $^{13}C\{^{1}H\}$ NMR (100 MHz, $CDCl_3$) δ 141.4 (d, J = 20.6 Hz, 1C), 141.0 (1C), 140.6 (1C), 138.5 (d, J = 7.9 Hz, 1C), 136.5 (1C), 134.8 (d, J = 21.4 Hz, 1C), 133.9 (d, J = 19.9 Hz, 1C), 133.7 (d, J = 1.9 Hz, 1C), 133.5 (d, J = 106.9 Hz, 1C), 132.6 (d, J = 2.8 Hz, 1C), 132.4 (d, J = 8.4 Hz, 1C), 131.8 (d, J = 11.4 Hz, 1C), 131.6 (d, J = 11.0 Hz, 2C), 131.0 (1C), 130.3 (d, J = 9.9 Hz, 1C), 129.3 (d, J = 11.3 Hz, 1C), 129.1 (d, J = 104.6 Hz, 1C), 128.8 (d, J = 12.8 Hz, 2C), 127.9 (1C), 127.2 (1C), 125.5 (1C), 125.2 (1C), 124.9 (d, J = 105.6 Hz, 1C), 124.7 (d, J = 10.0 Hz, 1C), 124.2 (1C), 123.8 (1C), 123.4 (1C), 123.1 (1C); $^{31}P\{^{1}H\}$ NMR (162 MHz, $CDCl_3$) δ 32.7; HRMS (APCI) m/z ($[M+H]^+$) calcd for $C_{30}H_{18}OPS_2$: 489.0531, found: 489.0550.

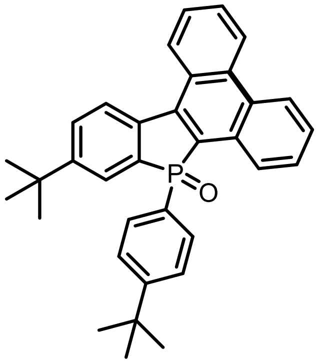

11-(*tert*-butyl)-9-(4-(*tert*-butyl)phenyl)tribenzo[*b,e,g*]phosphindole 9-oxide (2i)

Synthesized from **1i** (25 mg, 0.050 mmol, 1.0 equiv), $NaHCO_3$ (4.2 mg, 0.050 mmol, 1.0 equiv), $Bi(OTf)_3$ (33 mg, 0.050 mmol, 1.0 equiv), and MeCN (1.0 mL), with two Kessil KSPR160L lamps, according to **General Procedure A**, purified by silica gel (60 N, spherical neutral) column chromatography with $CHCl_3$ and GPC ($CHCl_3$): 24 mg (quant, 0.050 mmol scale); white solid; m.p. 318.1-319.8 °C; ^{1}H NMR (400 MHz, $CDCl_3$) δ 8.99 (d, J = 7.8 Hz, 1H), 8.82 (d, J = 8.1 Hz, 1H), 8.66 (d, J = 8.3 Hz, 1H), 8.45 (dd, J = 8.3, 3.8 Hz, 1H), 8.36 (d, J = 7.9 Hz, 1H), 7.88 (dd, J = 11.5, 1.8 Hz, 1H), 7.83-7.76 (m, 2H), 7.70-7.55 (m, 5H), 7.38-7.35 (m, 2H), 1.36 (s, 9H), 1.24 (s, 9H); ^{13}C{^{1}H} NMR (100 MHz, $CDCl_3$) δ 155.5 (d, J = 2.8 Hz, 1C), 152.3 (d, J = 10.2 Hz, 1C), 139.9 (d, J = 23.5 Hz, 1C), 139.2 (d, J = 20.0 Hz, 1C), 134.7 (d, J = 105.7 Hz, 1C), 133.8 (1C), 131.0 (d, J = 11.1 Hz, 2C), 130.40 (1C), 130.36 (d, J = 102.5 Hz, 1C), 130.04 (d, J = 2.3 Hz, 1C), 129.97 (d, J = 102.0 Hz, 1C), 129.6 (1C), 128.4 (1C), 128.10 (1C), 128.09 (d, J = 11.9 Hz, 1C), 128.0 (1C), 127.4 (d, J = 26.3 Hz, 1C), 127.1 (1C), 127.0 (d, J = 5.0 Hz, 1C), 125.88 (d, J = 12.7 Hz, 2C), 125.87 (1C), 125.2 (d, J = 11.4 Hz, 1C), 124.0 (1C), 123.0 (1C), 35.01 (1C), 34.96 (1C), 31.2 (3C), 31.0 (3C); ^{31}P{^{1}H} NMR (162 MHz, $CDCl_3$) δ 34.7; HRMS (APCI) m/z ($[M+H]^+$) calcd for $C_{34}H_{34}OP$: 489.2342, found: 489.2361.

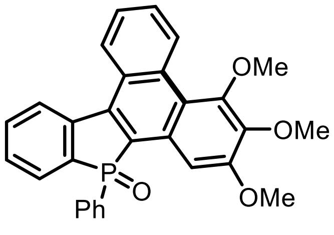

5,6,7-Trimethoxy-9-phenyltribenzo[*b,e,g*]phosphindole 9-oxide (2j)

Synthesized from **1j** (37 mg, 0.080 mmol, 1.0 equiv), $NaHCO_3$ (6.7 mg, 0.080 mmol, 1.0 equiv), $Bi(OTf)_3$ (53 mg, 0.080 mmol, 1.0 equiv), and MeCN (1.60 mL), with two Kessil KSPR160L lamps, according to **General Procedure A**, purified by silica gel (60 N, spherical neutral) column

chromatography with hexane/EtOAc (1/1, v/v) and GPC ($CHCl_3$): 37 mg (quant, 0.080 mmol scale); yellow solid; m.p. 158.2-161.5 °C; ^{1}H NMR (400 MHz, $CDCl_3$) δ 9.73-9.70 (m, 1H), 8.96-8.93 (m, 1H), 8.50 (dd, J = 8.1, 3.4 Hz, 1H), 7.84-7.65 (m, 6H), 7.55 (d, J = 0.8 Hz, 1H), 7.49-7.34 (m, 4H), 4.00 (s, 3H), 3.97 (s, 3H), 3.89 (s, 3H); ^{13}C{^{1}H} NMR (100 MHz, $CDCl_3$) δ 153.3 (1C), 152.4 (d, J = 2.0 Hz, 1C), 143.9 (1C), 142.6 (d, J = 23.2 Hz, 1C), 139.1 (d, J = 19.7 Hz, 1C), 134.2 (d, J = 106.0 Hz, 1C), 133.7 (1C), 133.1 (d, J = 1.7 Hz, 1C), 132.2 (d, J = 2.8 Hz, 1C), 131.1 (d, J = 10.8 Hz, 2C), 130.9 (d, J = 101.2 Hz, 1C), 130.1 (d, J = 9.8 Hz, 1C), 129.5 (d, J = 103.1 Hz, 1C), 128.83 (d, J = 12.4 Hz, 2C), 128.77 (d, J = 11.2 Hz, 1C), 128.6 (1C), 127.7 (1C), 127.4 (d, J = 11.9 Hz, 1C), 127.2 (d, J = 9.2 Hz, 1C), 126.3 (1C), 125.6 (d, J = 10.8 Hz, 1C), 125.3 (1C), 119.5 (d, J = 8.0 Hz, 1C), 103.6 (d, J = 6.4 Hz, 1C), 61.4 (1C), 60.6 (1C), 55.9 (1C); ^{31}P{^{1}H} NMR (162 MHz, $CDCl_3$) δ 34.0; HRMS (APCI) m/z ([M+H]$^+$) calcd for $C_{29}H_{24}O_4P$: 467.1407, found: 467.1415.

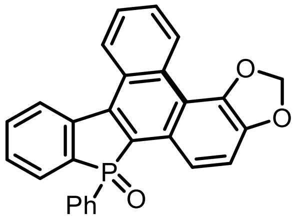

14-Phenylphosphindolo[3',2':9,10]phenanthro[3,4-*d*][1,3]dioxole 14-oxide (2k)

Synthesized from **1k** (21 mg, 0.050 mmol, 1.0 equiv), $NaHCO_3$ (4.2 mg, 0.050 mmol, 1.0 equiv), $Bi(OTf)_3$ (33 mg, 0.050 mmol, 1.0 equiv), and MeCN (1.0 mL), with one Kessil KSPR160L lamp, according to **General Procedure A**, purified by silica gel (60 N, spherical neutral) column chromatography with hexane/EtOAc (1/1, v/v) and GPC ($CHCl_3$): 21 mg (quant, 0.050 mmol scale); yellow solid; m.p. 251.7-253.7 °C; ^{1}H NMR (400 MHz, $CDCl_3$) δ 8.93 (d, J = 7.9 Hz, 1H), 8.56 (d, J = 7.6 Hz, 1H), 8.48 (dd, J = 8.0, 3.4 Hz, 1H), 7.97 (s, 1H), 7.82-7.63 (m, 7H), 7.47-7.34 (m, 4H), 6.07 (s, 1H), 6.03 (s, 1H); ^{13}C{^{1}H} NMR (100 MHz, $CDCl_3$) δ 149.2 (1C), 148.6 (1C), 142.7 (d, J = 23.2 Hz, 1C), 137.4 (d, J = 19.8 Hz, 1C), 134.2 (d, J = 106.2 Hz, 1C), 133.6 (1C), 133.1 (d, J = 1.9 Hz, 1C), 132.2 (d, J = 2.8 Hz, 1C), 131.1 (d, J = 10.8 Hz, 2C), 130.8 (d, J = 101.4 Hz, 1C), 130.0 (d, J = 9.8 Hz, 1C), 129.5 (d, J = 101.7 Hz, 1C), 128.9 (d, J = 12.4 Hz, 2C), 128.5 (d, J = 11.2 Hz, 1C), 128.2 (1C), 127.5 (d, J = 8.8 Hz, 1C), 127.3 (d, J = 11.9 Hz, 1C), 126.7 (1C), 126.0 (d, J = 8.9 Hz, 1C), 125.7 (1C), 125.3 (d, J = 10.8 Hz, 1C), 123.8 (1C), 104.1 (d, J = 6.1 Hz, 1C), 101.7 (1C), 101.3 (1C); ^{31}P{^{1}H} NMR (162 MHz, $CDCl_3$) δ 34.2; HRMS (APCI) m/z ([M+H]$^+$) calcd for $C_{27}H_{18}O_3P$: 421.0988, found: 421.0996.

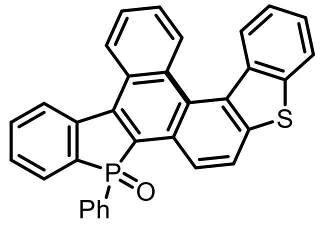

A 1:1.07 Diastereomixture of 5-phenylbenzo[*b*]phosphindolo[3',2':9,10]phenanthro[4,3-*d*]thiophene 5-oxide (2l)

Synthesized from **1l** (53 mg, 0.11 mmol, 1.0 equiv), $NaHCO_3$ (9.2 mg, 0.11 mmol, 1.0 equiv), $Bi(OTf)_3$ (73 mg, 0.11 mmol, 1.0 equiv), and MeCN (2.2 mL), with two Kessil KSPR160L lamps, according to **General Procedure A**, purified by silica gel (60 N, spherical neutral) column chromatography with hexane/EtOAc (1/1, v/v) and GPC ($CHCl_3$): 38.3 mg (72%, 0.11 mmol scale); yellow solid; m.p. 251.6-256.7 °C; ^{1}H NMR (400 MHz, $CDCl_3$) δ 9.10 (d, J = 8.3 Hz, 1H), 8.96-8.86 (m, 0.49 × 1H, 0.51 × 1H), 8.63 (d, J = 8.0 Hz, 1H), 8.46-8.44 (m, 0.49 × 1H, 0.51 × 1H), 8.26-8.14 (m, 0.49 × 1H, 0.51 × 1H), 7.94-7.59 (m, 8H), 7.46-7.32 (m, 6H); ^{13}C{^{1}H} NMR (100 MHz, $CDCl_3$) δ 142.0, 141.8, 141.3, 141.1, 139.7, 139.0, 136.3, 133.1, 132.6, 132.3, 131.1, 131.0, 130.42, 130.37, 130.2, 130.1, 129.3, 128.9, 128.8, 128.3, 128.0, 127.9, 126.5, 126.4, 125.4, 125.3, 125.2, 124.9, 124.59, 124.55, 124.50, 124.4, 123.3, 123.1 (All observed signals cannot be completely assigned because of complexity associated with diastereomers and C–P coupling. Thus, the observed signals were simply drawn.); ^{31}P{^{1}H} NMR (162 MHz, $CDCl_3$) δ 35.0, 33.9; HRMS (APCI) m/z ([M+H]$^+$) calcd for $C_{32}H_{20}OPS$: 483.0967, found: 483.0969.

^{1}H NMR (<u>600 MHz, DMSO-*d*$_6$, 80℃</u>) δ 9.06-9.03 (m, 2H), 8.66-8.64 (m, 1H), 8.58 (d, J = 8.3 Hz, 1H), 8.22-8.13 (m, 3H), 7.91 (t, J = 7.6 Hz, 1H), 7.86-7.82 (m, 2H), 7.62 (d, J = 5.1 Hz, 1H), 7.68-7.64 (m, 2H), 7.59-7.50 (m, 3H), 7.45-7.41 (m, 3H); ^{13}C{^{1}H} NMR (<u>150 MHz, DMSO-*d*$_6$, 80℃</u>) δ 141.3 (1C), 141.2 (1C), 141.1 (1C), 139.7 (1C), 138.7 (1C), 138.6 (1C), 136.0 (1C), 135.0 (d, J = 105.4 Hz, 1C), 134.0 (d, J = 1.8 Hz, 1C), 132.7 (d, J = 2.6 Hz, 1C), 132.1 (d, J = 1.8 Hz, 1C), 131.8 (d, J = 100.3 Hz, 1C), 131.0 (d, J = 10.6 Hz, 2C), 130.1 (d, J = 9.9 Hz, 1C), 129.9 (d, J = 101.5 Hz, 1C), 129.7 (d, J = 11.0 Hz, 1C), 129.6 (d, J = 12.1 Hz, 2C), 129.4 (1C), 129.1 (1C), 128.03 (1C), 127.95 (1C), 127.88 (1C), 127.82 (1C), 127.3 (d, J = 8.8 Hz, 1C), 126.3 (d, J = 10.3 Hz, 1C), 125.6 (1C), 125.2 (1C), 124.4 (d, J = 5.5 Hz, 1C), 124.2 (1C), 123.9 (d, J = 12.0 Hz, 1C); ^{31}P{^{1}H} NMR (<u>243 MHz, DMSO-*d*$_6$, 80℃</u>) δ 32.1.

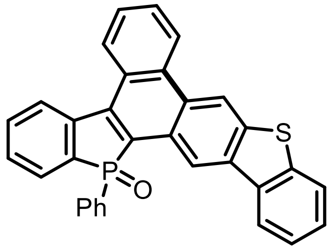

16-Phenylbenzo[*b*]phosphindolo[3',2':9,10]phenanthro[2,3-*d*]thiophene 16-oxide (2l')

Synthesized from **1l** (53 mg, 0.11 mmol, 1.0 equiv), $NaHCO_3$ (9.2 mg, 0.11 mmol, 1.0 equiv), $Bi(OTf)_3$ (73 mg, 0.11 mmol, 1.0 equiv), and MeCN (2.2 mL), with two Kessil KSPR160L lamps, according to **General Procedure A**, purified by silica gel (60 N, spherical neutral) column chromatography with hexane/EtOAc (1/1, v/v) and GPC ($CHCl_3$): 14.7 mg (28%, 0.11 mmol scale); yellow solid; m.p. 273.4-276.7 °C; ^{1}H NMR (400 MHz, $CDCl_3$) δ 9.11 (s, 1H), 9.03-9.00 (m, 2H), 8.88 (d, J = 8.1 Hz, 1H), 8.55 (dd, J = 8.0, 3.4 Hz, 1H), 8.26-8.22 (m, 1H), 7.90-7.79 (m, 6H), 7.72-7.68 (m, 1H), 7.52-7.42 (m, 4H), 7.40-7.35 (m, 2H); ^{13}C{^{1}H} NMR (100 MHz, $CDCl_3$) δ 142.8 (1C), 142.5 (1C), 140.0 (d, J = 6.9 Hz, 1C), 139.0 (1C), 138.8 (1C), 136.0 (1C), 134.9 (1C), 134.8 (1C), 133.3 (d, J = 2.5 Hz, 1C), 133.2 (d, J = 1.8 Hz, 1C), 132.3 (d, J = 2.6 Hz, 1C), 131.05 (d, J = 10.8 Hz, 2C), 131.00 (d, J = 101.5 Hz, 1C), 130.2 (d, J = 9.8 Hz, 1C), 129.7 (d, J = 8.5 Hz, 1C), 129.5 (d, J = 105.6 Hz, 1C), 129.0 (d, J = 12.3 Hz, 2C), 128.7 (d, J = 14.0 Hz, 1C), 128.1 (d, J = 12.3 Hz, 1C), 127.9 (1C), 127.6 (1C), 126.48 (d, J = 105.6 Hz, 1C), 126.43 (d, J = 11.7 Hz, 1C), 125.9 (1C), 125.5 (d, J = 10.9 Hz, 1C), 124.8 (1C), 124.2 (1C), 122.7 (d, J = 7.1 Hz, 1C), 119.2 (d, J = 5.5 Hz, 1C), 116.7 (1C); ^{31}P{^{1}H} NMR (162 MHz, $CDCl_3$) δ 34.1; HRMS (APCI) m/z ($[M+H]^+$) calcd for $C_{32}H_{20}OPS$: 483.0967, found: 483.0969.

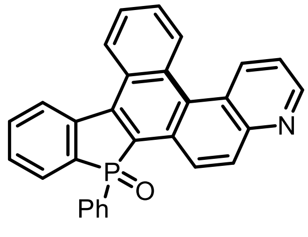

15-Phenyldibenzo[2,3:4,5]phosphindolo[6,7-*f*]quinoline 15-oxide (2m)

Synthesized from **1m** (13 mg, 0.030 mmol, 1.0 equiv), $NaHCO_3$ (2.5 mg, 0.030 mmol, 1.0 equiv), $Bi(OTf)_3$ (20 mg, 0.030 mmol, 1.0 equiv), and MeCN (0.60 mL), with two Kessil KSPR160L lamps, according to **General Procedure A**, purified by silica gel (60 N, spherical neutral) column chromatography with hexane/EtOAc (1/1, v/v) and GPC ($CHCl_3$): 3.2 mg (25%, 0.030 mmol scale);

yellow solid; m.p. 134.5-136.2 °C; ^{1}H NMR (400 MHz, $CDCl_3$) δ 9.24 (d, *J* = 8.4 Hz, 1H), 9.07-9.04 (m, 1H), 9.00-8.99 (m, 1H), 8.95-8.91 (m, 1H), 8.51 (dd, *J* = 8.0, 3.4 Hz, 1H), 8.46 (d, *J* = 9.0 Hz, 1H), 8.11 (d, *J* = 8.9 Hz, 1H), 7.89-7.82 (m, 3H), 7.78-7.70 (m, 3H), 7.59 (dd, *J* = 8.5, 4.2 Hz, 1H), 7.52-7.45 (m, 2H), 7.40-7.35 (m, 2H); ^{13}C{^{1}H} NMR (100 MHz, $CDCl_3$) δ 149.8 (1C), 149.0 (1C), 141.8 (d, *J* = 22.3 Hz, 1C), 140.0 (d, *J* = 20.0 Hz, 1C), 135.8 (1C), 134.8 (d, *J* = 106.4 Hz, 1C), 133.7 (d, *J* = 1.3 Hz, 1C), 133.2 (d, *J* = 1.4 Hz, 1C), 132.4 (d, *J* = 2.8 Hz, 1C), 131.1 (d, *J* = 10.8 Hz, 2C), 130.5 (d, *J* = 102.2 Hz, 1C), 130.4 (1C), 130.3 (1C), 129.5 (d, *J* = 101.7 Hz, 1C), 129.4 (1C), 129.2 (1C), 129.0 (d, *J* = 12.5 Hz, 2C), 128.8 (1C), 128.6 (1C), 128.4 (d, *J* = 8.9 Hz, 1C), 128.2 (d, *J* = 8.5 Hz, 1C), 127.6 (1C), 127.5 (1C), 125.6 (d, *J* = 10.7 Hz, 1C), 125.5 (1C), 125.0 (1C), 120.8 (1C); ^{31}P{^{1}H} NMR (162 MHz, $CDCl_3$) δ 34.0; HRMS (APCI) m/z ($[M+H]^+$) calcd for $C_{29}H_{19}NOP$: 428.1199, found: 428.1224.

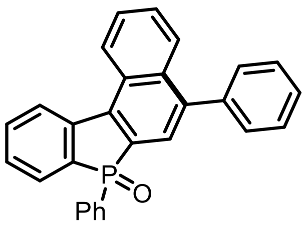

5,7-Diphenyldibenzo[*b,e*]phosphindole 7-oxide (2n)

Synthesized from **1n** (20 mg, 0.050 mmol, 1.0 equiv), $NaHCO_3$ (4.2 mg, 0.050 mmol, 1.0 equiv), $Bi(OTf)_3$ (33 mg, 0.050 mmol, 1.0 equiv), and MeCN (1.0 mL), with two Kessil KSPR160L lamps, according to **General Procedure A**, Purified by silica gel (60 N, spherical neutral) column chromatography with hexane/EtOAc (1/1, v/v) and GPC ($CHCl_3$): 14.4 mg (72%, 0.050 mmol scale); yellow solid; m.p. 199.5-201.2 °C; ^{1}H NMR (400 MHz, $CDCl_3$) δ 8.94 (d, *J* = 8.6 Hz, 1H), 8.54 (dd, *J* = 8.0, 3.5 Hz, 1H), 8.03 (d, *J* = 7.9 Hz, 1H), 7.84-7.80 (m, 1H), 7.74-7.67 (m, 5H), 7.60-7.56 (m, 1H), 7.50-7.35 (m, 9H); ^{13}C{^{1}H} NMR (100 MHz, $CDCl_3$) δ 143.3 (1C), 143.1 (1C), 142.7 (d, *J* = 11.0 Hz, 1C), 139.8 (1C), 138.4 (1C), 138.2 (1C), 135.2 (d, *J* = 1.6 Hz, 1C), 134.2 (d, *J* = 104.4 Hz, 1C), 133.4 (d, *J* = 1.6 Hz, 1C), 132.2 (d, *J* = 2.8 Hz, 1C), 131.8 (d, *J* = 104.2 Hz, 1C), 131.3 (d, *J* = 10.8 Hz, 2C), 130.4 (d, *J* = 9.4 Hz, 1C), 130.3 (d, *J* = 103.2 Hz, 1C), 130.0 (2C), 129.9 (1C), 128.8 (d, *J* = 12.3 Hz, 2C), 128.4 (1C), 128.0 (1C), 127.8 (1C), 127.7 (1C), 127.6 (1C), 125.5 (d, *J* = 10.6 Hz, 1C), 125.2 (d, *J* = 10.0 Hz, 1C), 124.9 (1C); ^{31}P{^{1}H} NMR (162 MHz, $CDCl_3$) δ 32.9; HRMS (APCI) m/z ($[M+H]^+$) calcd for $C_{28}H_{20}OP$: 403.1246, found: 403.1253.

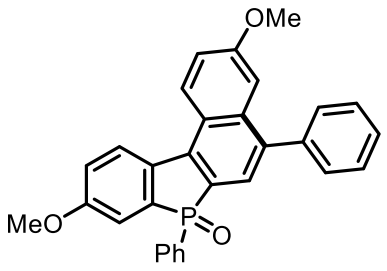

3,9-Dimethoxy-5,7-diphenyldibenzo[*b,e*]phosphindole 7-oxide (2o)

Synthesized from **1o** (23 mg, 0.050 mmol, 1.0 equiv), $NaHCO_3$ (4.2 mg, 0.050 mmol, 1.0 equiv), $Bi(OTf)_3$ (33 mg, 0.050 mmol, 1.0 equiv), and MeCN (1.0 mL), with one Kessil KSPR160L lamp, according to **General Procedure A**, purified by silica gel (60 N, spherical neutral) column chromatography with hexane/EtOAc (1/1, v/v) and GPC ($CHCl_3$): 3.6 mg (16%, 0.050 mmol scale); yellow solid; m.p. 176.3-179.6 °C; ^{1}H NMR (400 MHz, $CDCl_3$) δ 8.78 (d, J = 9.0 Hz, 1H), 8.39 (dd, J = 8.4, 4.1 Hz, 1H), 7.70 (dd, J = 12.8, 8.2 Hz, 2H), 7.60 (d, J = 9.7 Hz, 1H), 7.50-7.45 (m, 5H), 7.42-7.30 (m, 6H), 7.16 (d, J = 8.7 Hz, 1H), 3.86 (s, 3H), 3.78 (s, 3H); ^{13}C{^{1}H} NMR (100 MHz, $CDCl_3$) δ 158.8 (1C), 140.2 (1C), 137.2 (1C), 136.6 (d, J = 100.9 Hz, 1C), 135.7 (1C), 135.3 (1C), 132.1 (d, J = 2.0 Hz, 1C), 131.7 (d, J = 109.4 Hz, 1C), 131.3 (d, J = 10.7 Hz, 2C), 130.0 (d, J = 13.1 Hz, 1C), 129.8 (2C), 129.7 (d, J = 103.7 Hz, 1C), 128.8 (d, J = 12.4 Hz, 2C), 128.5 (1C), 127.8 (1C), 127.6 (1C), 127.4 (1C), 126.6 (1C), 126.5 (d, J = 9.6 Hz, 1C), 126.1 (1C), 125.9 (d, J = 10.3 Hz, 1C), 119.6 (1C), 118.9 (1C), 115.0 (1C), 106.4 (1C), 55.7 (1C), 55.2 (1C); ^{31}P{^{1}H} NMR (162 MHz, $CDCl_3$) δ 32.8; HRMS (APCI) m/z ($[M+H]^+$) calcd for $C_{30}H_{24}O_3P$: 463.1458, found: 463.1475.

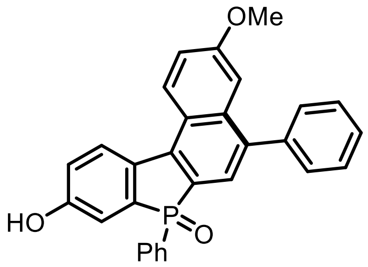

9-Hydroxy-3-methoxy-5,7-diphenyldibenzo[*b,e*]phosphindole 7-oxide (2o-H)

Synthesized from **1o** (23 mg, 0.050 mmol, 1.0 equiv), $NaHCO_3$ (4.2 mg, 0.050 mmol, 1.0 equiv), $Bi(OTf)_3$ (33 mg, 0.050 mmol, 1.0 equiv), and MeCN (1.0 mL), with one Kessil KSPR160L lamp, according to **General Procedure A**, purified by silica gel (60 N, spherical neutral) column chromatography with hexane/EtOAc (1/1, v/v) and GPC ($CHCl_3$): 6.3 mg (28%, 0.05 mmol scale);

yellow gum; ^{1}H NMR (400 MHz, $CDCl_3$) δ 8.37 (d, *J* = 9.3 Hz, 1H), 8.20 (dd, *J* = 8.8, 4.3 Hz, 1H), 7.70-7.64 (m, 2H), 7.52-7.47 (m, 1H), 7.40-7.32 (m, 3H), 7.28-7.24 (m, 1H), 7.20-7.17 (m, 6H), 7.14-7.10 (m, 2H), 3.84 (s, 3H); ^{31}P{^{1}H} NMR (162 MHz, $CDCl_3$) δ 35.2; HRMS (APCI) m/z ($[M+H]^+$) calcd for $C_{29}H_{22}O_3P$: 449.1301, found: 449.1321. (Any clear ^{13}C{^{1}H} NMR spectra were not obtained because of small quantity of product.)

Double Buchwald-Hartwig amination of 2f: General Procedure B

To the Schlenk tube (10 mL), 3,6-dibromo-9-phenyltribenzo[*b,e,g*]phosphindole 9-oxide (**2f**, 0.050 mmol, 1.0 equiv), N-heterocycle (0.15 mmol, 3.0 equiv), $[Pd(\eta^3\text{-}C_3H_5)Cl]_2$ (0.70 mg, 20 μmol, 4.0 mol%), MoPhos (1.4 mg, 40 μmol, 8.0 mol%), NaO*t*Bu (11.5 mg, 0.12 mmol, 2.4 equiv), and *o*-xylene (0.50 mL) were added under nitrogen atmosphere. The resulting mixture was stirred at 130 °C for 16 h. After the reaction completed, EtOAc (20 mL) and water (20 mL) were added to the reaction mixture, and the organic layer was extracted with EtOAc three times, dried with Na_2SO_4, and filtered through a sort pad of activated alumina. The filtrate was concentrated under reduced pressure to give a solid. The resulting solid was dissolved in chloroform and purified by column chromatography on silica gel (). Further purification was conducted with GPC ($CHCl_3$) to form the corresponding aminated product **3**.

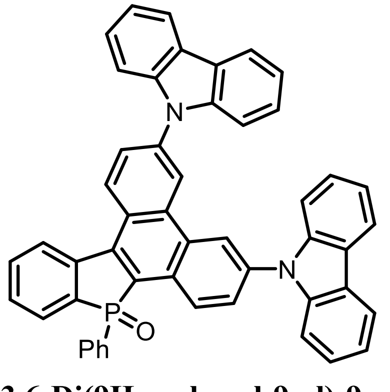

3,6-Di(9H-carbazol-9-yl)-9-phenyltribenzo[*b,e,g*]phosphindole 9-oxide (3fa)

Synthesized from **1f** (27 mg, 0.050 mmol, 1.0 equiv), carbazole (25 mg, 0.15 mmol, 3.0 equiv), $[Pd(\eta^3\text{-}C_3H_5)Cl]_2$ (0.70 mg, 20 μmol, 4.0 mol%), MoPhos (1.4 mg, 40 μmol, 8.0 mol%), NaO*t*Bu (11.5 mg, 0.12 mmol, 2.4 equiv), and *o*-xylene (0.50 mL), according to **General Procedure B**, purified by silica gel (60 N, spherical neutral) column chromatography with $CHCl_3$ and GPC ($CHCl_3$): 29.4 mg (83%, 0.050 mmol scale); yellow solid; m.p. 235.1-236.3 °C; ^{1}H NMR (400 MHz, $CDCl_3$) δ 9.29 (d, *J*

= 8.9 Hz, 1H), 8.93 (d, *J* = 2.0 Hz, 1H), 8.78 (s, 1H), 8.6 (dd, *J* = 8.0, 3.4 Hz, 1H), 8.60 (d, *J* = 8.6 Hz, 1H), 8.16-8.07 (m, 5H), 7.95-7.83 (m, 4H), 7.77 (t, *J* = 7.7 Hz, 1H), 7.59-7.52 (m, 4H), 7.48-7.44 (m, 4H), 7.42-7.27 (m, 8H); $^{13}C\{^{1}H\}$ NMR (100 MHz, $CDCl_3$) δ 142.2 (d, *J* = 23.0 Hz, 1C), 140.7 (2C), 140.6 (2C), 139.5 (d, *J* = 19.8 Hz, 1C), 138.3 (1C), 137.6 (1C), 135.0 (d, *J* = 1.5 Hz, 1C), 134.7 (d, *J* = 106.2 Hz, 1C), 133.4 (d, *J* = 2.1 Hz, 1C), 132.5 (d, *J* = 2.8 Hz, 1C), 131.5 (d, *J* = 8.3 Hz, 1C), 131.2 (d, *J* = 10.8 Hz, 2C), 130.5 (d, *J* = 102.1 Hz, 1C), 130.4 (d, *J* = 9.9 Hz, 1C), 130.3 (d, *J* = 101.7 Hz, 1C), 129.4 (d, *J* = 11.2 Hz, 1C), 129.1 (d, *J* = 12.4 Hz, 2C), 128.9 (d, *J* = 5.5 Hz, 1C), 128.6 (d, *J* = 8.9 Hz, 1C), 127.9 (1C), 127.6 (1C), 127.0 (d, *J* = 11.7 Hz, 1C), 126.6 (1C), 126.4 (2C), 126.2 (2C), 125.6 (d, *J* = 10.6 Hz, 1C), 123.8 (2C), 123.7 (2C), 121.7 (1C), 121.1 (1C), 120.7 (2C), 120.6 (2C), 120.4 (4C), 109.6 (4C); $^{31}P\{^{1}H\}$ NMR (162 MHz, $CDCl_3$) δ 33.8; HRMS (APCI) m/z ($[M+H]^+$) calcd for $C_{50}H_{32}N_2OP$: 707.2247, found: 707.2243.

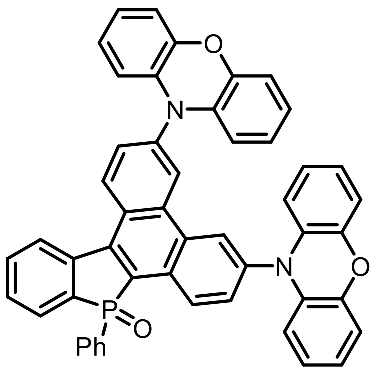

3,6-Di(10H-phenoxazin-10-yl)-9-phenyltribenzo[*b,e,g*]phosphindole 9-oxide (3fb)

Synthesized from **1f** (27 mg, 0.050 mmol, 1.0 equiv), phenoxazine (27 mg, 0.15 mmol, 3.0 equiv), $[Pd(\eta^3\text{-}C_3H_5)Cl]_2$ (0.70 mg, 20 µmol, 4.0 mol%), MoPhos (1.4 mg, 40 µmol, 8.0 mol%), NaO*t*Bu (11.5 mg, 0.12 mmol, 2.4 equiv), and *o*-xylene (0.50 mL), according to **General Procedure B**, purified by silica gel (60 N, spherical neutral) column chromatography with $CHCl_3$ and GPC ($CHCl_3$): 13.5 mg (27%, 0.050 mmol scale); orange solid; m.p. 213.2-215.4 °C; ^{1}H NMR (400 MHz, $CDCl_3$) δ 9.27 (d, *J* = 8.8 Hz, 1H), 8.74 (s, 1H), 8.60-8.57 (m, 3H), 7.93-7.90 (m, 1H), 7.88-7.87 (m, 1H), 7.85-7.80 (m, 2H), 7.79-7.74 (m, 1H), 7.59-7.52 (m, 3H), 7.48-7.43 (m, 2H), 6.75-6.73 (m, 2H), 6.70-6.68 (m, 3H), 6.66-6.56 (m, 5H), 6.53-6.49 (m, 2H), 6.01-5.99 (m, 2H), 5.90-5.88 (m, 2H); $^{13}C\{^{1}H\}$ NMR (100 MHz, $CDCl_3$) δ 144.0 (2C), 143.9 (2C), 141.9 (1C), 140.0 (1C), 139.8 (1C), 139.5 (1C), 138.8 (1C), 135.9 (1C), 134.5 (d, *J* = 107.1 Hz, 1C), 134.0 (d, *J* = 10.0 Hz, 2C), 133.5 (1C), 132.6 (d, *J* = 2.9 Hz, 1C), 132.0 (d, *J* = 103.3 Hz, 1C), 131.9 (d, *J* = 101.0 Hz, 1C), 131.3 (1C), 131.2 (d, *J* = 10.8 Hz, 1C), 130.6 (d, *J* = 13.2 Hz, 1C), 130.43 (d, *J* = 2.0 Hz, 1C), 130.39 (1C), 130.0 (d, *J* = 5.3 Hz, 1C), 129.6 (1C),

129.5 (1C), 129.2 (1C), 129.16 (1C), 129.13 (d, J = 12.3 Hz, 2C), 129.09 (1C), 127.7 (d, J = 12.2 Hz, 1C), 126.9 (1C), 126.0 (1C), 125.7 (d, J = 10.8 Hz, 1C), 123.4 (2C), 123.2 (2C), 121.9 (2C), 121.7 (2C), 115.8 (2C), 115.6 (2C), 113.33 (2C), 113.32 (2C); $^{31}P\{^{1}H\}$ NMR (162 MHz, $CDCl_3$) δ 33.8; HRMS (APCI) m/z ($[M+H]^+$) calcd for $C_{50}H_{32}N_2O_3P$: 739.2145, found: 739.2140.

X-Ray Analysis

The single X-ray quality crystals of **2g** were grown from benzene by slow evaporation at room temperature. The structure was refined by full-matrix least-squares method using SHELXL-2017/1.

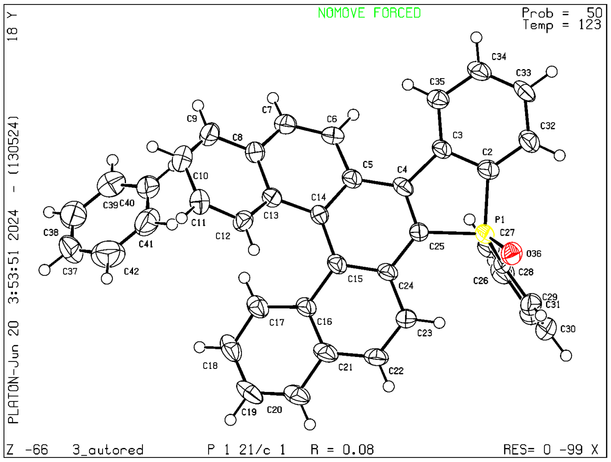

Figure S2. ORTEP drawing of **2g** (CCDC 2377855, 50% thermal probability).

Table S1. Crystal data for **2g**

Crystal system	monoclinic
Space group IT number	14
Space group name H-M alt	P 1 21/c 1
Space group name Hall	-P 2ybc
Cell length a	8.3632(3)
Cell length b	11.8180(5)
Cell length c	28.4133(11)
Cell angle alpha	90
Cell angle beta	97.436(3)
Cell angle gamma	90
Cell volume	2784.65(19)
Cell formula units Z	4
Refine ls R factor all	0.1394
Refine ls R factor gt	0.0782
Refine ls wR factor gt	0.2035
Refine ls wR factor ref	0.2607
Refine ls goodness of fit ref	1.168

The single X-ray quality crystals of **2l’** were grown from $CDCl_3$ by slow evaporation at room temperature. The structure was refined by full-matrix least-squares method using SHELXL-2017/1.

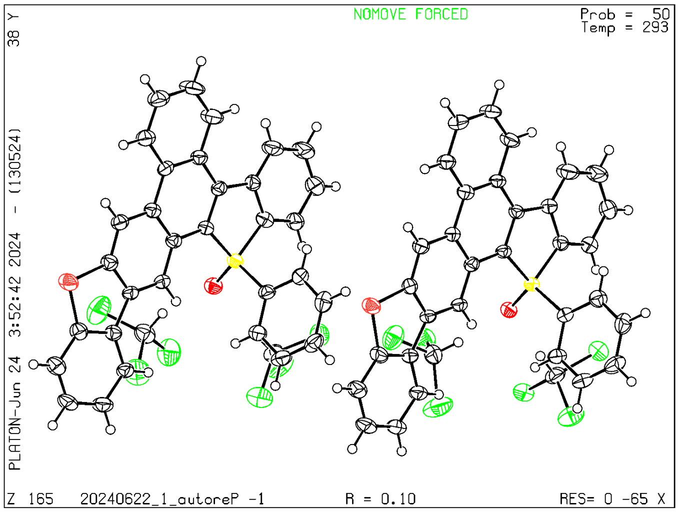

Figure S3. ORTEP drawing of **2l’** (CCDC 2377856, 50% thermal probability).

Table S2. Crystal data for **2l’**

Crystal system	triclinic
Space group IT number	2
Space group name H-M alt	P -1
Space group name Hall	-P 1
Cell length a	14.4990(5)
Cell length b	15.0425(5)
Cell length c	15.7255(4)
Cell angle alpha	82.549(2)
Cell angle beta	69.415(3)
Cell angle gamma	81.710(3)
Cell volume	3165.65(18)
Cell formula units Z	2
Refine ls R factor all	0.1068
Refine ls R factor gt	0.0967
Refine ls wR factor gt	0.2769
Refine ls wR factor ref	0.2838
Refine ls goodness of fit ref	1.110

Detailed Optimization Studies

Table S3. Condition optimization for the dehydrogenative Mallory reaction of **1a** under visible light irradiation: Screening of Lewis and Brønsted acid additives.[a]

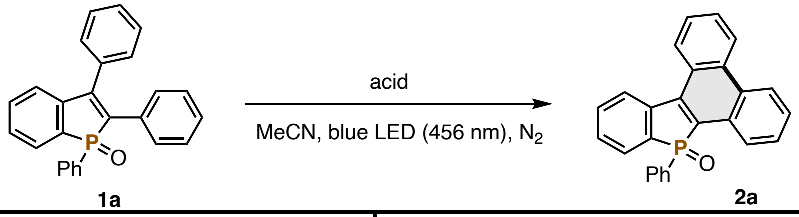

entry	acid	yield of **2a** (%)[b]	entry	acid	yield of **2a** (%)[b]
1	none	0	12	$Cu(OTf)_2$	4
2	***$Bi(OTf)_3$***	***33***	13	AgOTf	6
3	***$Al(OTf)_3$***	***49***	14	LiOTf	7
4	***$In(OTf)_3$***	***68***	15	NaOTf	0
5	***$Sc(OTf)_3$***	***34***	16	KOTf	0
6	$Zn(OTf)_2$	17	17	TfOH	0
7	$La(OTf)_3$	4	18	TFA	40
8	$Y(OTf)_3$	15	19	PTSA	0
9	$Yb(OTf)_3$	5	20	$(PhO)_2P(O)(OH)$	20
10	$Ni(OTf)_2$	1	21	AcOH	0
11	$Fe(OTf)_2$	0	22	HCl	0

[a] Reaction conditions: **1a** (0.050 mmol), acid (0.050 mmol), MeCN (1.0 mL), 22 h, N_2, blue LED irradiation (456 nm, 40 W), ambient temperature. [b] Estimated by $^{31}P\{^{1}H\}$ NMR with $P(O)(OEt)_3$ as the internal standard.

Table S4. Condition optimization for the dehydrogenative Mallory reaction of **1a** under visible light irradiation: Screening of combinations of Lewis acid and Bronsted base additives.[a]

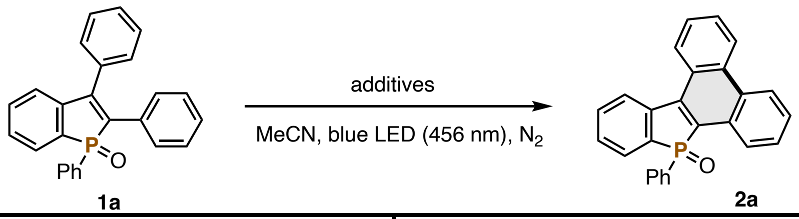

entry	additives	yield of **2a** (%)[b]	entry	additives	yield of **2a** (%)[b]
1	***$Bi(OTf)_3/NaHCO_3$***	***(>99)***	7	$Bi(OTf)_3/Na_2CO_3$	<5
2	$Al(OTf)_3/NaHCO_3$	38	8	$In(OTf)_3/Na_2CO_3$	11

3	$In(OTf)_3/NaHCO_3$	31	9[d]	$Bi(OTf)_3/$ $NaHCO_3$	0
4	$Sc(OTf)_3/NaHCO_3$	8	10[e]	$Bi(OTf)_3/$ $NaHCO_3$	0
5	$InCl_3/NaHCO_3$	5	11[f]	$Bi(OTf)_3/$ $NaHCO_3$	16
6	$BF_3{\bullet}OEt_2/NaHCO_3$[c]	8	12[g]	$Bi(OTf)_3/$ $NaHCO_3$	7

[a] Reaction conditions: **1a** (0.050 mmol), additives (0.050 mmol), MeCN (1.0 mL), 22 h, N_2, blue LED irradiation (456 nm, 40 W), ambient temperature. [b] Estimated by $^{31}P\{^1H\}$ NMR with $P(O)(OEt)_3$ as the internal standard. Isolated yield is in parentheses. [c] With $BF_3{\bullet}OEt_2$ (0.10 mmol) and $NaHCO_3$ (0.050 mmol). [d] In dark at 50 ˚C. [e] With **1c** instead of **1a** under green LED irradiation (525 nm). [f] With **1c** instead of **1a** under sunlight for 2 days. [g] With $Bi(OTf)_3$ (0.010 mmol) and $NaHCO_3$ (0.050 mmol).

Table S5. Condition optimization for the dehydrogenative Mallory reaction of **1a** under visible light irradiation: Screening of solvent.[a]

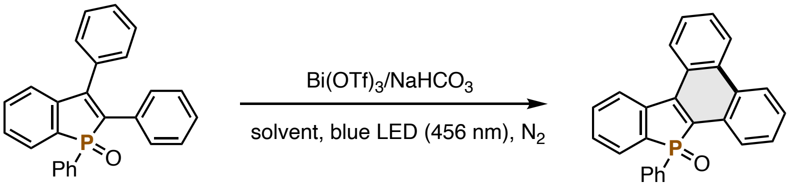

1a **2a**

entry	solvent	yield of **2a** (%)[b]	entry	additives	yield of **2a** (%)[b]
1	***MeCN***	***(>99)***	7	$PhCF_3$	37
2	1,4-dioxane	63	8	DMF	28
3	THF	45	9	DMSO	43
4	Et_2O	44	10	acetone	73
5	DME	79	11	MeOH	64
6	toluene	30			

Reaction conditions: **1a** (0.050 mmol), $Bi(OTf)_3$ (0.050 mmol), $NaHCO_3$ (0.050 mmol), solvent (1.0 mL), 22 h, N_2, blue LED irradiation (456 nm, 40 W), ambient temperature. [b] Estimated by $^{31}P\{^1H\}$ NMR with $P(O)(OEt)_3$ as the internal standard. Isolated yield is in parentheses.

Unsuccessful Substrates

The following substrates gave a complicated mixture or showed much lower conversion (<10%) under the standard conditions (Table S4, entry 1).

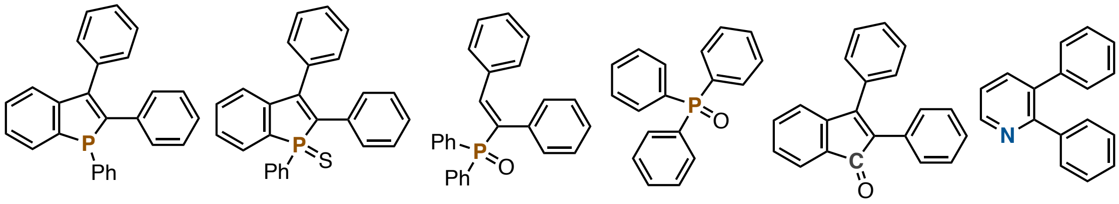

Figure S4. Unsuccessful substrates.

Detection of Evolved H_2

According to **General Procedure A**, to a 2–5 mL microwave vial (Biotage) were added **1c** (25 mg, 0.050 mmol), $NaHCO_3$ (4.2 mg, 0.050 mmol), $Bi(OTf)_3$ (33 mg, 0.050 mmol), and MeCN (1.0 mL) in a nitrogen-filled glove box. The vial was capped with an aluminum cap and taken out from the glove box. The resulting mixture was stirred under blue LED irradiation at ambient temperature (one or two Kessil KSPR160L, 456 nm, 40 W, see Figure S1). After 22 h, the gas component in the tube was sampled with a gas tight syringe and analyzed by GC. The result was shown in Figure S5.

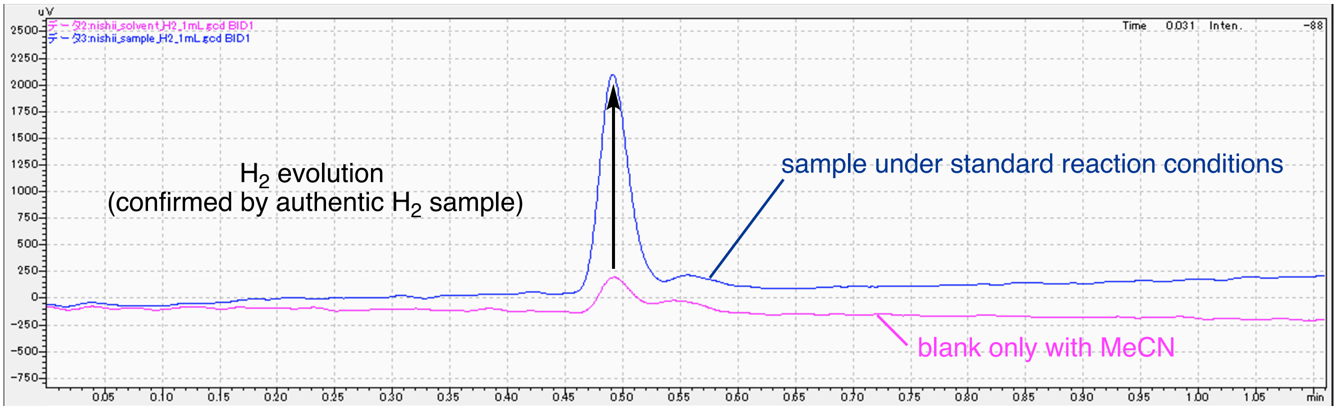

Figure S5. Detection of H_2 by GC.

Deuterium-Labeling Experiments

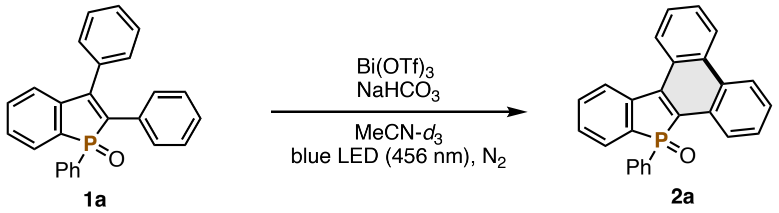

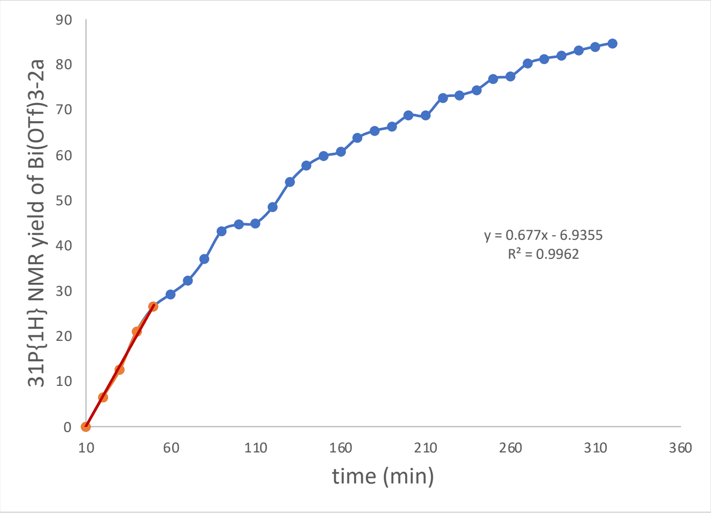

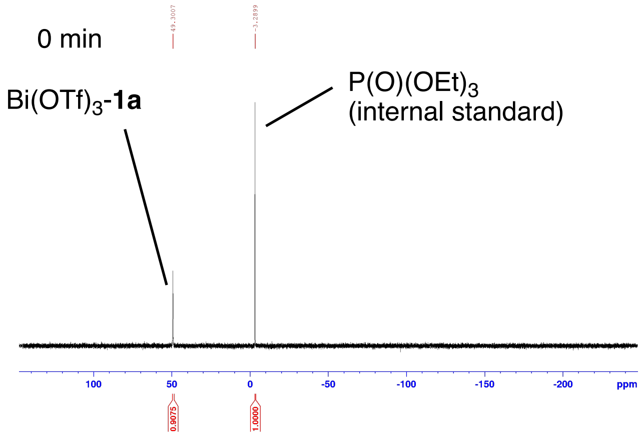

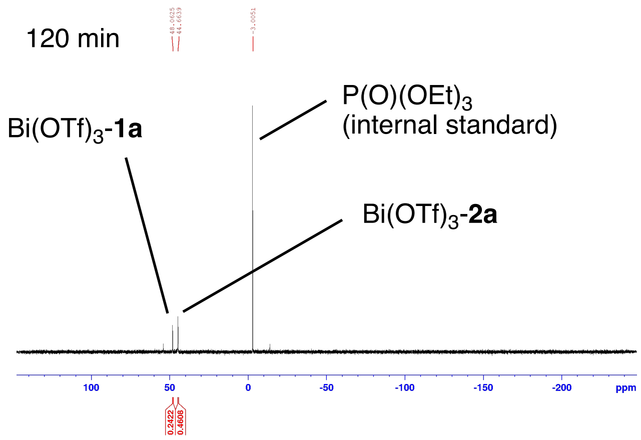

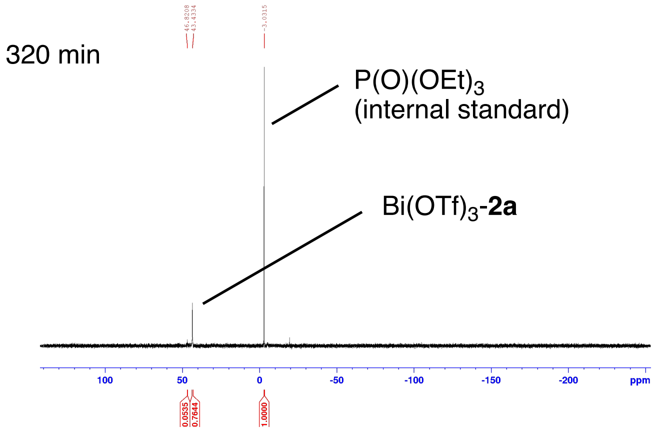

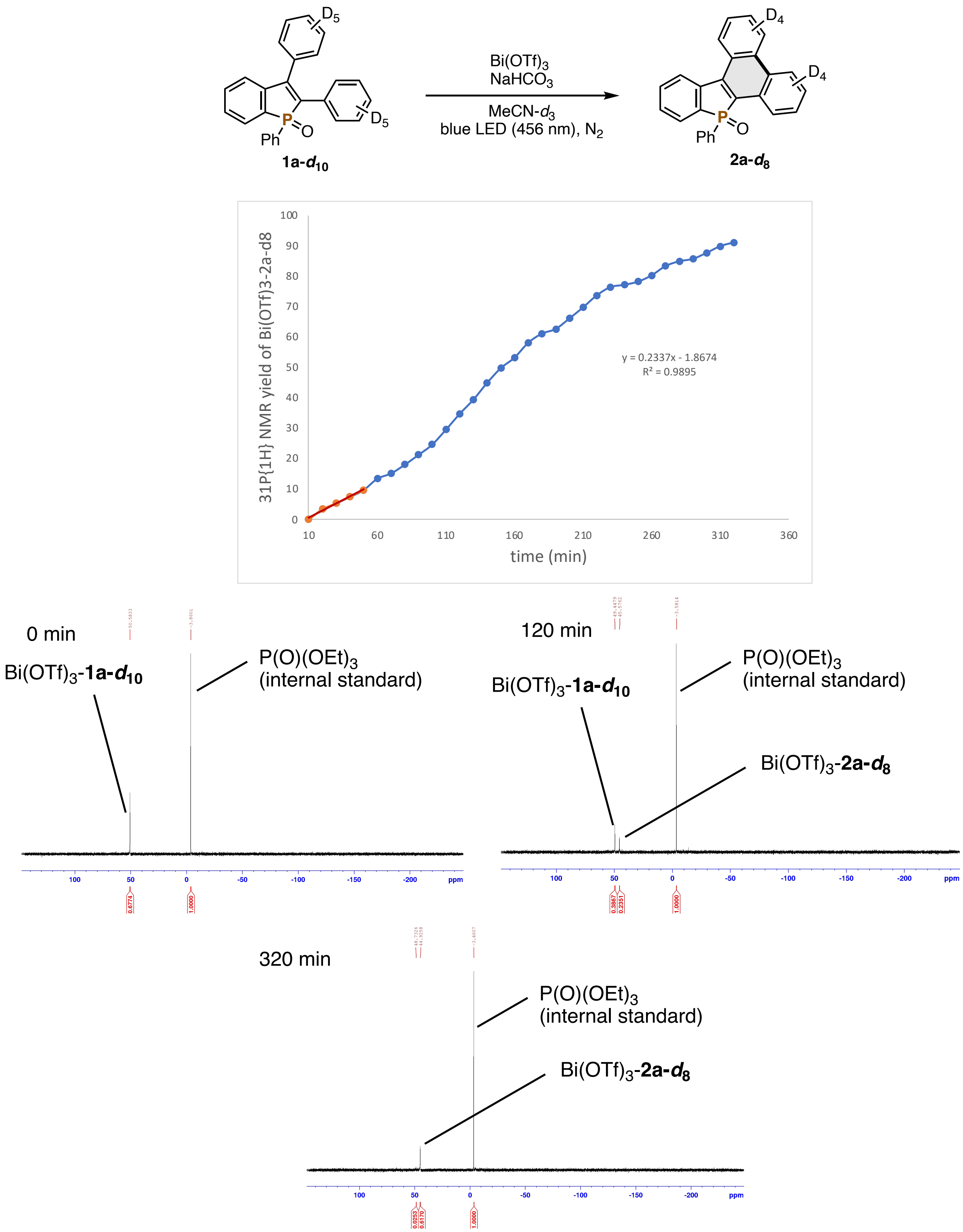

Figure S6. Reaction progresses of **1a** and **1a-*d*10** and their initial reaction rates.

We monitored the reaction progress by tracking the formation of $Bi(OTf)_3$-cooridinated **2a** or **2a**-$\boldsymbol{d_8}$ using $^{31}P\{^1H\}$ NMR in MeCN-d_3 solution. As the internal reference, $P(O)(OEt)_3$ was also used. According to the results in Figure S6, the kinetic isotope effect (KIE) value is calculated as follows.

$$\mathrm{KIE} = k_\mathrm{H}/k_\mathrm{D} = 0.6670/0.2337 = 2.8540\ldots \cong 2.90$$

NMR Studies

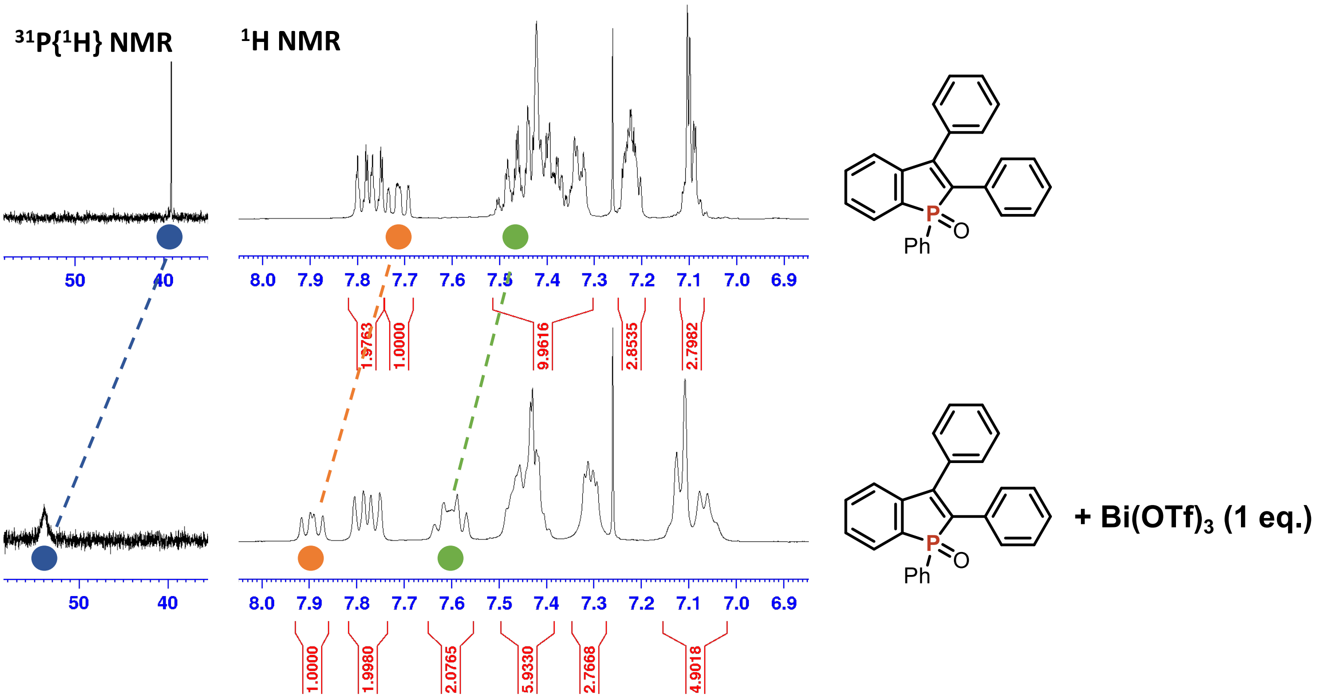

Figure S7. $^{31}P\{^1H\}$ and ^1H NMR spectra of **1a** (upper) and **1a** + 1.0 equiv of $Bi(OTf)_3$ (bottom) in $CDCl_3$.

UV-vis Absorption Spectra Studies

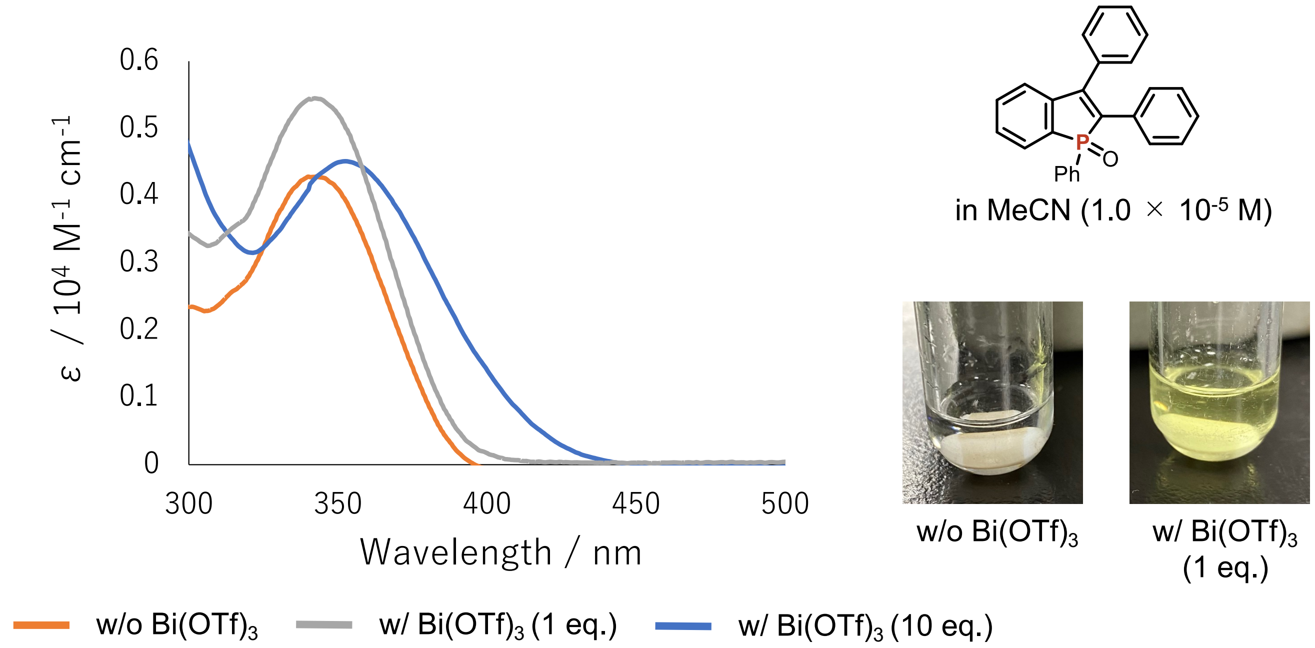

Figure S8. UV-vis Absorption spectra of **1a** (orange line), **1a** + 1 equiv of $Bi(OTf)_3$ (gray line), and **1a** + 10 equiv of $Bi(OTf)_3$ (blue line) in MeCN (1.0 x 10^{-5} M).

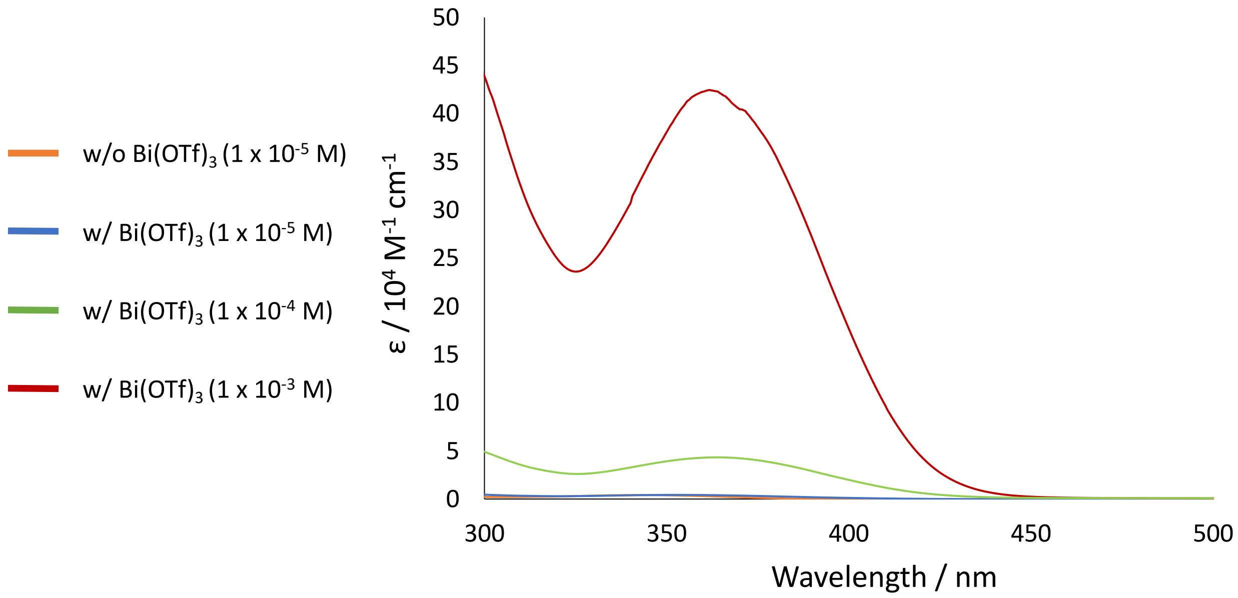

Figure S9. Concentration effects in UV-vis absorption spectra of **1a** + 10 equiv of $Bi(OTf)_3$ in MeCN: orange line (no Bi, 1.0 x 10^{-5} M), blue line (1.0 x 10^{-5} M), green line (1.0 x 10^{-4} M), and red line (1.0 x 10^{-3} M).

Computational Studies

All calculations were carried out using the Gaussian 16 program.[S5] The ground-state (S_0) and exited-state (S_1) geometries were optimized by the density functional theory (DFT) and time-dependent density functional theory (TD-DFT) methods with M06-2X functional and a standard 6-31G(d) basis set (LanL2DZ basis set for Bi). The M06-2X functional is a high-nonlocality functional with double the amount of nonlocal exchange (2X), with reliable performance for the thermochemistry, hydrogen bonding, kinetics, and weak interactions.[S6] The optimized molecular structures were verified by vibrational analysis; equilibrium structures did not have imaginary frequencies and transition state structures had only one imaginary frequency. The intrinsic reaction coordinate (IRC) calculations were carried out to check whether the transition state leading to the reactant and the product. Single-point energies were calculated using the 6-311+G(d,p) basis set (SDD basis set for Bi), and the solvent effect of MeCN was taken account by the integral equation formalism PCM (IEF-PCM). HOMO, LUMO, and UV-Vis spectrum of **1a** and $Bi(OTf)_3$-coordinated **1a** were analyzed by TD-DFT at the M06-2X/6-311+G(d,p)&SDD/PCM(MeCN) level. The calculated structures and molecular orbitals were visualized with Gauss View 6.1.1. Summary of the level of theory: M06-2X/6-311+G(d,p)&SDD/PCM(MeCN)//M06-2X/6-31G(d)&LanL2DZ.

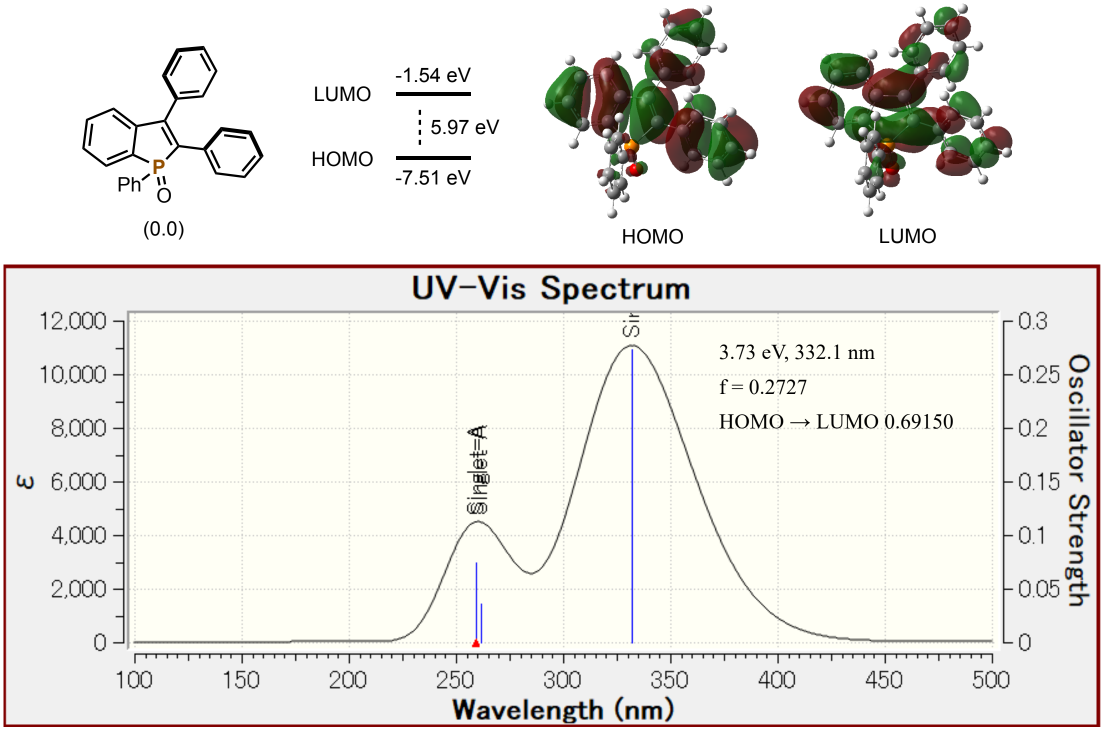

Figure S10. Calculated optimized molecular structure, HOMO/LUMO level, and UV-vis spectrum of **1a** [M06-2X/6-311+G(d,p)&SDD/PCM(MeCN)]

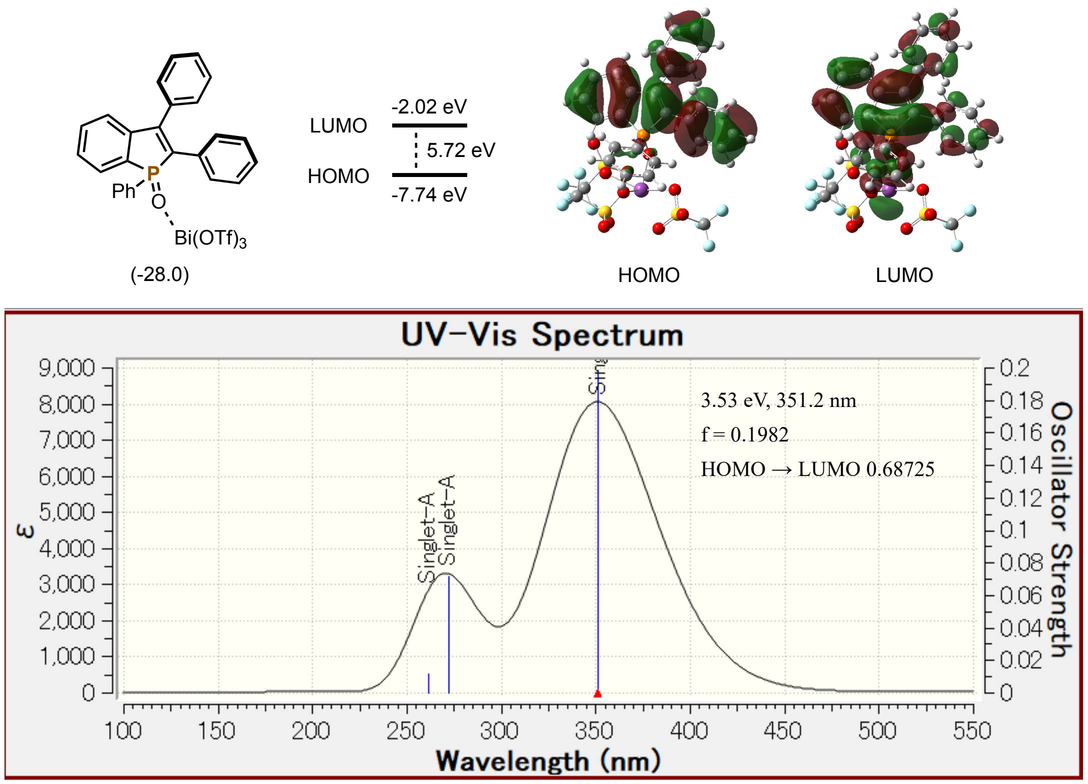

Figure S11. Calculated optimized molecular structure, HOMO/LUMO level, and UV-vis spectrum of Bi(OTf)$_3$-coordinated **1a** [M06-2X/6-311+G(d,p)&SDD/PCM(MeCN)]

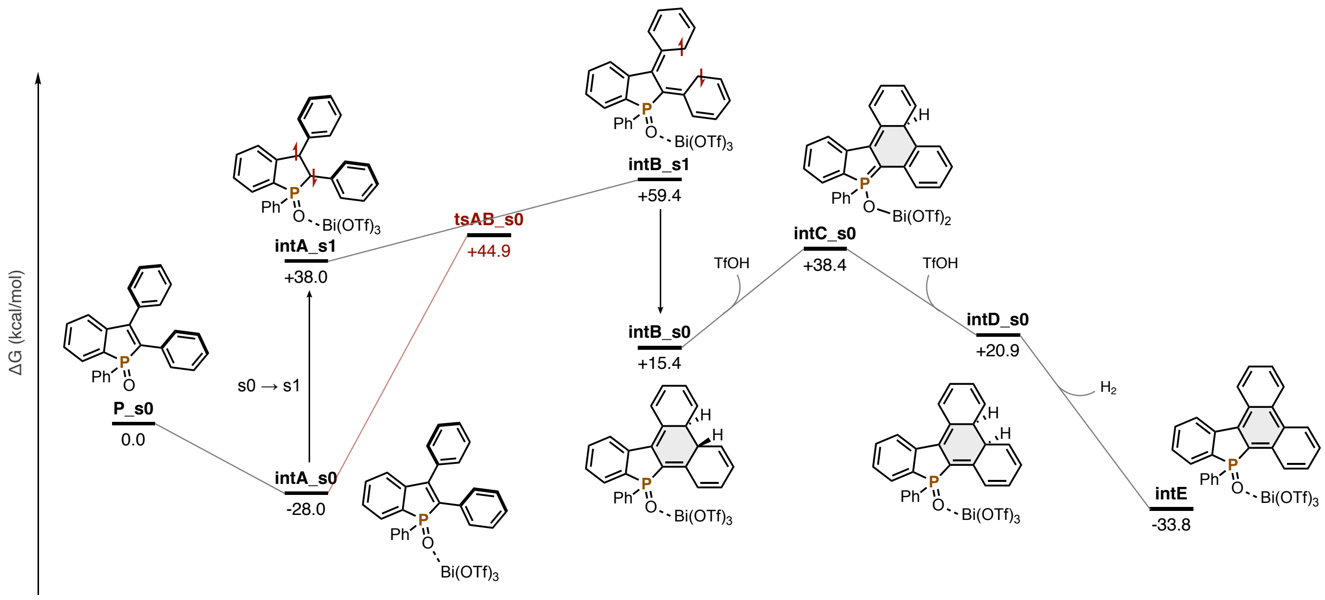

Figure S12. Gibbs energy profile for Bi(OTf)$_3$-assisted dehydrogenative Mallory reaction.

Table S6. Summary of the calculation

	EE + Free Energy Correction [Hartree] 6-31G(d)&LanL2DZ	Number of Imaginary Frequency	EE + Free Energy Correction [Hartree] 6-311+G(d,p)&SDD
**1a (P_s0)**	-1418.0043	0	-1418.3201
$Bi(OTf)_3$	-2889.01336	0	-2889.66803
**intA_s0**	-4307.0566	0	-4308.0327
**intA_s1**	-4306.9475	0	-4307.9275
**tsAB_s0**	-4306.9386	1	-4307.9166
**intB_s1**	-4306.9409	0	-4307.8935
**intB_s0**	-4306.9869	0	-4307.9635
**intC_s0**	-3345.1826	0	-3345.9308
TfOH	-961.76603	0	-961.99608
**intD_s0**	-4306.9777	0	-4307.9549
H_2	-1.164864	0	-1.169701
**intE**	-4305.9029	0	-4306.8722

Cartesian Coordinates for Optimized Structures

1a (P_s0)

C	0.49412300	2.03062100	-0.34239000
C	-0.84409600	2.06744000	-0.76533500
C	-1.51200500	3.26613400	-0.94829000
C	-0.83265300	4.46201000	-0.69837000
C	0.49556600	4.43435700	-0.28331600
C	1.17026400	3.22382800	-0.10592400
C	1.05326000	0.65042400	-0.23689500
C	0.19244400	-0.33360300	-0.58952000
H	-2.54331600	3.27487700	-1.28926800
H	-1.33822100	5.41228000	-0.83659800
H	1.02083400	5.36704100	-0.10173800
H	2.21165800	3.21741700	0.20003400
C	2.45750900	0.44055400	0.19017500
C	3.33662800	-0.29087800	-0.61545800

C	2.91723900	0.95384100	1.40815700
C	4.64894000	-0.50472600	-0.20965200
H	2.97777000	-0.69338600	-1.55817300
C	4.22942700	0.73188500	1.81667000
H	2.23592700	1.51571200	2.04226600
C	5.09808100	0.00418700	1.00754300
H	5.32265500	-1.07171100	-0.84439500
H	4.57224200	1.12712400	2.76791100
H	6.12233800	-0.16606000	1.32419700
C	0.37918100	-1.79461100	-0.54616900
C	-0.21070700	-2.58871300	-1.54012100
C	1.09128100	-2.41789300	0.48868900
C	-0.06490700	-3.97231300	-1.51194500
H	-0.78026700	-2.10978400	-2.33246400
C	1.22913100	-3.79998800	0.51347500
H	1.52801900	-1.81350200	1.27796100
C	0.65672400	-4.58131700	-0.48927900
H	-0.52031900	-4.57520300	-2.29146400
H	1.78010100	-4.27014300	1.32227600
H	0.76688300	-5.66120700	-0.46725200
C	-2.53666800	-0.05609100	0.31702900
C	-2.17699100	0.14081200	1.65331200
C	-3.77662200	-0.60780900	-0.00324300
C	-3.06163700	-0.20981400	2.66587600
H	-1.20562100	0.56607800	1.89771100
C	-4.66062400	-0.95887000	1.01529300
H	-4.03000900	-0.75678800	-1.04906500
C	-4.30361500	-0.75929300	2.34547700
H	-2.78549000	-0.05821600	3.70467600
H	-5.62630500	-1.38942500	0.76941500
H	-4.99271800	-1.03394900	3.13837400
P	-1.42769300	0.37552100	-1.05304200

O	-2.01455000	0.03812200	-2.38668800

Bi(OTf)$_3$

S	-0.02088300	2.55828700	-0.58341400
O	-1.28365100	1.87173500	-0.96455900
O	0.38750800	3.74328800	-1.28452700
O	1.00988700	1.40730300	-0.59028800
C	-0.14174700	2.97925400	1.20483200
F	-0.36834600	1.88578100	1.89893100
F	-1.14121900	3.83284900	1.35291700
F	0.99259600	3.53807400	1.57737800
S	2.23864900	-1.34077800	0.93221100
O	2.44936400	-2.64471600	1.50183800
O	1.70019100	-0.24047400	1.69685600
O	1.45578100	-1.48083500	-0.43652900
C	3.84270400	-0.78339000	0.23401700
F	3.64287900	0.28765300	-0.52248300
F	4.37115700	-1.74858800	-0.50025400
F	4.65820300	-0.47949700	1.22933100
O	-2.06848900	-2.50941900	1.80084800
O	-1.77275500	-2.24253600	-0.70909700
O	-1.08893600	-0.46152600	0.71071400
C	-3.66907800	-0.83125700	0.48129900
F	-3.59416100	-0.00084800	-0.55199800
F	-3.92249400	-0.15855300	1.58454600
F	-4.60789700	-1.73284900	0.26578800
Bi	-0.11448200	-0.35102300	-1.18127400
S	-2.04757300	-1.68072800	0.63341600

intA_s0

C	3.27478700	2.42922200	0.18086900
C	1.90319000	2.25961600	0.44505900

C	1.04416900	3.33396900	0.60617600
C	1.57631500	4.62430600	0.51693500
C	2.93122200	4.80591300	0.26367600
C	3.79060900	3.71552500	0.08958300
C	4.01659800	1.15295200	-0.03629100
C	3.25142600	0.03283600	-0.00554400
H	-0.01676200	3.17814600	0.77572200
H	0.92099800	5.48158400	0.62329400
H	3.33043800	5.81184800	0.18232600
H	4.84031000	3.87437200	-0.13384300
C	5.47356500	1.16411100	-0.29788600
C	5.99070600	0.51804900	-1.42600800
C	6.34826800	1.80388100	0.58783800
C	7.36020400	0.51463300	-1.66396600
H	5.31089000	0.02010900	-2.11084500
C	7.72002900	1.78889200	0.35241600
H	5.95108800	2.29658900	1.47123600
C	8.22725800	1.14723100	-0.77471100
H	7.75185200	0.01592500	-2.54453000
H	8.39185200	2.27826500	1.05023700
H	9.29648200	1.14005400	-0.96043600
C	3.64709300	-1.38519000	-0.10185400
C	2.81199100	-2.28867900	-0.77233200
C	4.80777900	-1.86326700	0.52203400
C	3.12744800	-3.64310100	-0.81594700
H	1.90344700	-1.93261000	-1.24686300
C	5.12528100	-3.21482300	0.46461600
H	5.44926200	-1.17453600	1.06286200
C	4.28680700	-4.10826000	-0.20147600
H	2.45603600	-4.32996200	-1.32052200
H	6.02364500	-3.57518700	0.95550800
H	4.53314000	-5.16479300	-0.23164700

C	1.06175700	-0.13021400	1.99252400
C	0.64460700	0.75312400	2.99300000
C	0.93109000	-1.51587800	2.16496500
C	0.06507900	0.25009700	4.15200400
H	0.74557700	1.82501900	2.85736500
C	0.34517600	-2.00664700	3.32442300
H	1.23878400	-2.20589500	1.38588700
C	-0.09721200	-1.12455700	4.30904800
H	-0.27751900	0.93297800	4.92256600
H	0.20368000	-3.07577300	3.43523400
H	-0.57813400	-1.51160900	5.20161500
P	1.55630400	0.50575500	0.39752800
O	0.55106400	0.09675200	-0.72289500
S	-1.63821700	2.54090600	-1.76632800
O	-1.44199900	1.52697500	-2.81467800
O	-0.83896400	3.74307600	-1.76050600
O	-1.66359500	1.79748800	-0.43322000
C	-3.38659000	3.06564600	-1.92295500
F	-4.16794500	2.00562300	-1.76647400
F	-3.58277800	3.58866500	-3.12427300
F	-3.67050600	3.96803500	-0.99649300
S	-3.18155300	-0.04167000	1.59411100
O	-3.67683700	-0.79908100	2.71073400
O	-3.87566300	-0.02403000	0.29999700
O	-1.69825800	-0.34118500	1.26144700
C	-3.12439500	1.73713400	2.16603500
F	-1.87003700	2.14590400	2.31553900
F	-3.74217000	1.81984900	3.33324200
F	-3.74121000	2.50666200	1.28809900
O	-0.99231000	-4.14066300	1.23219500
O	-0.37520100	-2.40428200	-0.45379700
O	-2.70991800	-2.77611500	-0.03332900

C	-1.37127100	-4.63406500	-1.32973800
F	-1.66424800	-4.00368900	-2.46493300
F	-2.26398200	-5.58419500	-1.11130400
F	-0.16443700	-5.17815200	-1.43714300
Bi	-1.50066700	-0.44127700	-0.87475400
S	-1.39166200	-3.42357200	0.04456600

intA_s1

C	-3.37580300	-2.28592100	0.31841700
C	-1.99153800	-2.10056700	0.68449600
C	-1.16200800	-3.19354100	0.94658000
C	-1.68284900	-4.47189600	0.83170900
C	-3.02386500	-4.67414100	0.42837700
C	-3.85572000	-3.61009100	0.15981500
C	-4.07563900	-1.07281500	0.07533300
C	-3.23349200	0.09430800	0.22368300
H	-0.11453400	-3.04153900	1.18924900
H	-1.03916700	-5.32854500	0.99922400
H	-3.39174300	-5.68665900	0.30073200
H	-4.86610000	-3.78126300	-0.19452500
C	-5.45037200	-1.00432400	-0.39800300
C	-5.80284900	-0.12436600	-1.44325400
C	-6.46314400	-1.78700500	0.19178900
C	-7.11482900	-0.05307900	-1.89354800
H	-5.02912900	0.46934200	-1.91931500
C	-7.77363500	-1.69800300	-0.25402100
H	-6.21691300	-2.42515200	1.03487200
C	-8.10400100	-0.83540800	-1.30177600
H	-7.36507500	0.61560600	-2.71071800
H	-8.54533000	-2.29274700	0.22399400
H	-9.12990600	-0.77136100	-1.64951500
C	-3.64254900	1.46925300	0.22102800

C	-2.71508400	2.47229000	-0.17103500
C	-4.93842800	1.88133700	0.63982300
C	-3.06354200	3.80879700	-0.14625300
H	-1.73375200	2.18304100	-0.53622200
C	-5.27266700	3.22511300	0.66248500
H	-5.64488700	1.14012700	0.99528100
C	-4.34452400	4.19204800	0.26921300
H	-2.33728800	4.55273900	-0.45796400
H	-6.25796500	3.52633300	1.00261300
H	-4.61557300	5.24278300	0.29062900
C	-0.92556500	0.26165000	2.12314400
C	-0.81711800	-0.58817300	3.23042100
C	-0.50461400	1.59443300	2.22448700
C	-0.26116200	-0.11547300	4.41415500
H	-1.15023100	-1.61937600	3.16412000
C	0.04148000	2.06154400	3.41260700
H	-0.55012800	2.26044800	1.37173000
C	0.17137300	1.20559300	4.50444800
H	-0.15919900	-0.78330700	5.26331900
H	0.39793100	3.08481300	3.46080100
H	0.61814100	1.56652900	5.42535200
P	-1.56077000	-0.39230100	0.57387800
O	-0.57382800	0.01285000	-0.59197000
S	1.34978400	-2.71206800	-1.63593600
O	1.08934700	-1.77082200	-2.73646800
O	0.52127400	-3.88344900	-1.47257000
O	1.52000300	-1.87907600	-0.36917300
C	3.05710600	-3.31265200	-1.91721300
F	3.87650500	-2.27141100	-1.97655700
F	3.10372300	-3.97723600	-3.06335100
F	3.42448400	-4.11309300	-0.92879000
S	3.33569300	-0.03094000	1.40703500

O	3.98036000	0.77084300	2.41113100
O	3.94115000	-0.25287000	0.09108500
O	1.87045100	0.39860500	1.13698800
C	3.10545400	-1.72031200	2.17112700
F	1.82145900	-1.95186400	2.41838400
F	3.77458800	-1.75960400	3.31246100
F	3.57579900	-2.64865200	1.35746600
O	1.40978200	4.09228200	1.04997700
O	0.58165800	2.43252000	-0.61144800
O	2.96638900	2.69206900	-0.37142100
C	1.59289800	4.63665400	-1.51609300
F	1.76412400	4.04093500	-2.69183400
F	2.52887200	5.55609800	-1.34801000
F	0.39652500	5.21791100	-1.49645000
Bi	1.46322100	0.32997500	-0.96379900
S	1.69001000	3.38750400	-0.18091600

tsAB_s0

C	-3.03141700	-2.80507000	0.21487500
C	-1.69030700	-2.46781000	0.48947300
C	-0.68983200	-3.42550600	0.56843700
C	-1.03743100	-4.76461900	0.38650100
C	-2.35546000	-5.11446200	0.09836500
C	-3.35136700	-4.14501500	-0.01197600
C	-3.90514100	-1.62614800	0.03596100
C	-3.18166400	-0.38692100	0.10929900
H	0.34615000	-3.13769300	0.72403900
H	-0.26602400	-5.52607700	0.42227800
H	-2.60685700	-6.15496100	-0.07943100
H	-4.35070900	-4.43315300	-0.31528200
C	-0.92780200	-0.03529000	2.02235400
C	-0.35059000	-0.90472700	2.95271600

C	-0.93998800	1.34433400	2.27025300
C	0.24941400	-0.39355700	4.09871500
H	-0.34478300	-1.97511200	2.77605600
C	-0.33520500	1.84722700	3.41432100
H	-1.37256700	2.03641000	1.55771400
C	0.27191900	0.98026200	4.32117500
H	0.71718800	-1.07060900	4.80624600
H	-0.30837500	2.91992700	3.57024600
H	0.76862100	1.37844700	5.20000400
P	-1.49508700	-0.68540700	0.44637900
O	-0.48528000	-0.26910100	-0.68557200
S	2.02870100	-2.40530800	-1.72490700
O	1.63306100	-1.47643000	-2.79471600
O	1.43387900	-3.71918300	-1.64627100
O	1.98462100	-1.61964000	-0.41700400
C	3.83096500	-2.65360100	-1.94365600
F	4.44104900	-1.48072400	-1.84335600
F	4.05914500	-3.17161800	-3.14215300
F	4.29082700	-3.47597500	-1.01359400
S	3.29400800	0.48686500	1.49891900
O	3.70304400	1.33429100	2.58611800
O	3.94190400	0.54673900	0.18443400
O	1.77127700	0.55518900	1.22141400
C	3.52689000	-1.26748900	2.10238400
F	2.35920900	-1.86383700	2.30260600
F	4.18901600	-1.22864300	3.24832100
F	4.22912200	-1.95051200	1.21574300
O	0.41168000	4.16726800	1.23519500
O	0.07099500	2.32550600	-0.40892500
O	2.30989600	3.17091500	-0.11352800
C	0.52847500	4.68475500	-1.33832000
F	0.89285500	4.13046400	-2.49116100

F	1.17885100	5.82354100	-1.17216700
F	-0.78118000	4.92783800	-1.37492100
Bi	1.43645100	0.54077700	-0.89606700
S	0.89732300	3.52871700	0.03304200
C	-3.84724500	0.87931100	0.05542300
C	-3.20370700	2.05540500	-0.36455500
C	-5.97571900	2.17208700	0.09605500
C	-3.93285900	3.21346800	-0.58158200
H	-2.13830900	2.04459300	-0.58052300
C	-5.33406000	3.27944000	-0.39203300
H	-7.04045400	2.20004000	0.31540900
H	-3.40273500	4.09337000	-0.93614300
H	-5.86929000	4.19832000	-0.60017500
C	-5.26941300	-1.63648800	-0.23991300
C	-6.16456900	-2.62380400	0.28228800
C	-5.83783100	-0.39012600	-0.83532400
C	-7.51700800	-2.51158700	0.16573300
H	-5.74364700	-3.43893900	0.86192400
C	-7.29149000	-0.38255600	-1.01799100
C	-8.06653800	-1.38651000	-0.54074800
H	-8.17677400	-3.25608300	0.59430600
H	-7.73228000	0.44257300	-1.56866000
H	-9.13876200	-1.35180800	-0.71546800
C	-5.28444100	0.93237400	0.31495200
H	-5.62115200	0.38533500	1.19627700
H	-5.28900700	-0.04585900	-1.71587800

intB_s1

C	-3.06321500	-2.72761600	0.15754100
C	-1.70370100	-2.44608300	0.40567800
C	-0.74052900	-3.43952200	0.48543100
C	-1.14319200	-4.76797300	0.32812800

C	-2.47854700	-5.06301900	0.07328700
C	-3.44214000	-4.05547700	-0.02389500
C	-3.90605300	-1.51706200	0.00542300
C	-3.19812700	-0.32205300	0.03561900
H	0.30516600	-3.18894500	0.63780300
H	-0.40508600	-5.56098000	0.37034200
H	-2.77890800	-6.09511700	-0.07722400
H	-4.46464500	-4.30995100	-0.27777300
C	-1.00307400	-0.05833600	2.01801100
C	-0.46284200	-0.94277600	2.95663800
C	-1.00832000	1.32170000	2.26429400
C	0.10860300	-0.44242100	4.12100400
H	-0.46353600	-2.01141900	2.76887200
C	-0.42970600	1.81181500	3.42727300
H	-1.41866200	2.01564600	1.53887500
C	0.13962400	0.93155500	4.34640400
H	0.54716500	-1.12537500	4.84123900
H	-0.39392100	2.88273400	3.59178800
H	0.61459300	1.32065100	5.24121500
P	-1.47782900	-0.67202000	0.40638800
O	-0.50299200	-0.17648800	-0.70652000
S	1.91711000	-2.36000600	-1.83130700
O	1.59876900	-1.34880400	-2.85183500
O	1.24945600	-3.63978500	-1.84271800
O	1.88386700	-1.64716900	-0.48082400
C	3.70845500	-2.69439100	-2.02382700
F	4.37746300	-1.56234200	-1.85227600
F	3.93803400	-3.16592800	-3.24042900
F	4.09985500	-3.58341600	-1.12421700
S	3.23927700	0.28674100	1.57156000
O	3.67074400	1.06365600	2.70124900
O	3.91104600	0.37300200	0.26867500

O	1.72848600	0.44305000	1.26626200
C	3.37340400	-1.50218200	2.09816300
F	2.17096900	-2.04240200	2.25248900
F	4.01435300	-1.54777400	3.25499000
F	4.05360300	-2.18179800	1.19315200
O	0.63980600	4.12866100	1.39593400
O	0.17258800	2.37366400	-0.31732100
O	2.45971700	3.02862100	0.01977900
C	0.82672000	4.72781700	-1.16312100
F	1.15499300	4.17374000	-2.32789100
F	1.58167900	5.79252000	-0.95409600
F	-0.45064700	5.09177400	-1.20324400
Bi	1.47935000	0.56602600	-0.86205800
S	1.07691300	3.49961600	0.17214700
C	-3.80697700	0.97016800	-0.10068200
C	-3.09554200	2.07388300	-0.57922700
C	-5.88294300	2.32845900	-0.25560100
C	-3.73751200	3.26222600	-0.88847300
H	-2.02432200	1.98778200	-0.74390100
C	-5.14659300	3.37166000	-0.73146500
H	-6.95224800	2.43897900	-0.09975700
H	-3.16116700	4.10335300	-1.25692600
H	-5.63486100	4.31140900	-0.97097900
C	-5.33197500	-1.49393200	-0.19525800
C	-6.19690300	-2.40051800	0.42621800
C	-5.88700700	-0.29463100	-0.87331000
C	-7.57259100	-2.24517000	0.36985100
H	-5.77786400	-3.20938600	1.01638400
C	-7.34717600	-0.25372000	-0.98403700
C	-8.14245000	-1.16511200	-0.35972800
H	-8.21714300	-2.95021600	0.88285600
H	-7.78573800	0.55209300	-1.56548500

H	-9.22258100	-1.08458000	-0.43308600
C	-5.27611600	1.03901800	0.08322000
H	-5.60317700	0.67910500	1.06639800
H	-5.39051300	-0.07175500	-1.82321000

intB_s0

C	-3.24480300	-2.48669800	0.51776100
C	-1.85205800	-2.26289900	0.56723100
C	-0.93626300	-3.29689700	0.74115900
C	-1.41633300	-4.59110500	0.91606300
C	-2.79132800	-4.81679000	0.96679900
C	-3.70422500	-3.78153100	0.78062300
C	-4.04558100	-1.27667400	0.21031700
C	-3.22062900	-0.06287200	0.21296600
H	0.13023900	-3.09235200	0.74241900
H	-0.72110000	-5.41455400	1.03545100
H	-3.16307200	-5.81850800	1.15834400
H	-4.76328800	-3.98504300	0.87233500
C	-0.88565100	0.03503600	2.10398700
C	-0.40404000	-0.90755400	3.01867000
C	-0.74290600	1.40348700	2.37535100
C	0.25609600	-0.48434500	4.16722600
H	-0.52299200	-1.96871000	2.83253800
C	-0.07910000	1.81759800	3.52217500
H	-1.10618000	2.15328700	1.68402500
C	0.43285100	0.87433600	4.41090300
H	0.64518200	-1.22078700	4.86277400
H	0.06464500	2.87795200	3.69624500
H	0.97243100	1.20066100	5.29409600
P	-1.51220800	-0.50990200	0.50704500
O	-0.57040700	-0.03746200	-0.64488400
S	1.52288700	-2.42561700	-1.90630700

O	1.28293700	-1.37296600	-2.90625700
O	0.68794500	-3.60263600	-1.87722300
O	1.66449300	-1.72442600	-0.55631000
C	3.23855300	-2.99786400	-2.20221500
F	4.06559300	-1.97113200	-2.06764300
F	3.33111000	-3.48551400	-3.43066500
F	3.55400100	-3.94122800	-1.32785700
S	3.35916200	0.04284100	1.38137600
O	3.95829800	0.76345100	2.47094300
O	3.92900900	0.07383600	0.02765400
O	1.85160000	0.34813500	1.19984600
C	3.35787800	-1.75449700	1.89445500
F	2.12376800	-2.17339500	2.14607500
F	4.08261600	-1.87413800	2.99513300
F	3.89135800	-2.49132900	0.93697200
O	1.11604200	4.07959600	1.48672500
O	0.38127800	2.43286100	-0.23768000
O	2.73723400	2.87791600	-0.04468600
C	1.17976200	4.74943700	-1.05855500
F	1.38452000	4.21616400	-2.26018300
F	2.03016600	5.74329800	-0.86724100
F	-0.06420900	5.21517500	-0.99930200
Bi	1.45721800	0.51983400	-0.90695600
S	1.41514700	3.45780600	0.21818600
C	-3.71800000	1.17798600	-0.02987800
C	-2.89481900	2.34140000	-0.26258300
C	-5.70706100	2.62994800	-0.59643400
C	-3.44522200	3.51726300	-0.63672000
H	-1.81429300	2.24092700	-0.23536900
C	-4.87795700	3.65203000	-0.84811900
H	-6.77026300	2.73273500	-0.79334400
H	-2.79770300	4.36237800	-0.84883600

H	-5.26006500	4.58563000	-1.24810100
C	-5.33185100	-1.21551000	-0.23032800
C	-6.15907900	-2.36741000	-0.56717600
C	-5.95051400	0.12072300	-0.60220100
C	-7.50014100	-2.24837100	-0.65513500
H	-5.68377700	-3.31922700	-0.77295200
C	-7.43067900	0.13951300	-0.27811600
C	-8.15593700	-0.98256000	-0.34106200
H	-8.10772800	-3.10730700	-0.92238200
H	-7.89963100	1.09001900	-0.04072100
H	-9.22573500	-0.97010400	-0.15852100
C	-5.22707000	1.33490700	-0.00045800
H	-5.49144800	1.35389400	1.07657100
H	-5.86741300	0.19288500	-1.70781800

intC_s0

C	1.27274200	0.41376300	2.02994600
C	0.50834700	-0.71983100	1.67987600
C	-0.62229100	-1.12072500	2.38210300
C	-1.00963000	-0.37823200	3.49333300
C	-0.24784900	0.72197500	3.88492600
C	0.88610500	1.11473300	3.17528400
C	2.42131200	0.63833800	1.11087600
C	2.58361200	-0.47015700	0.16973800
H	-1.18691300	-1.99608900	2.07208700
H	-1.89057300	-0.66301300	4.05879100
H	-0.53315200	1.28232600	4.76988800
H	1.47399100	1.94242700	3.54619200
C	1.45999000	-3.26051300	0.38218500
C	2.69994900	-3.74470800	0.80932600
C	0.39721100	-4.14285700	0.16108500
C	2.87803100	-5.11103600	1.00236600

H	3.51605900	-3.04648700	0.97578700
C	0.57789500	-5.50603000	0.37259500
H	-0.56181500	-3.76084600	-0.17585100
C	1.81735500	-5.98899800	0.78860900
H	3.84332000	-5.48939400	1.32234600
H	-0.24661400	-6.19209800	0.20755800
H	1.95665800	-7.05397500	0.94621400
P	1.23578100	-1.47648800	0.24133100
O	0.13146600	-1.46932600	-0.99613300
S	-0.50821900	2.37327000	-0.43787600
O	0.06876600	1.89024300	-1.71187200
O	0.27912500	3.06656200	0.53926800
O	-1.29873200	1.15047400	0.08551900
C	-1.89235800	3.47277800	-0.93196600
F	-2.64990100	2.80180000	-1.79732200
F	-1.41028000	4.55659700	-1.51093100
F	-2.60597700	3.79919900	0.12672200
S	-4.02776800	-0.89094300	-0.14876700
O	-5.02126600	-1.91644000	-0.33219500
O	-4.02257700	0.30854100	-0.99517600
O	-2.57665400	-1.43336500	-0.18605500
C	-4.17373100	-0.33416100	1.59662000
F	-3.85010800	-1.34327200	2.39474300
F	-5.43000600	0.01888300	1.81526500
F	-3.37532700	0.68982700	1.82164600
Bi	-1.35664000	-0.26079800	-1.54200700
C	3.69283900	-0.50558300	-0.76776900
C	3.88514400	-1.56587500	-1.67176400
C	5.68201100	0.53074300	-1.67262600
C	4.95805500	-1.58163000	-2.55035400
H	3.17627200	-2.39139800	-1.68521300
C	5.86855100	-0.52648300	-2.55659200

H	6.37279500	1.37096800	-1.69307500
H	5.07907800	-2.41445900	-3.23663500
H	6.70538000	-0.52201900	-3.24733300
C	3.27407300	1.69696200	1.09879400
C	3.18812200	2.88008600	1.95257700
C	4.42463900	1.77478900	0.11154900
C	4.30601500	3.56267800	2.27808700
H	2.21794100	3.22426500	2.29159300
C	5.69159300	2.18982700	0.84672700
C	5.61492800	3.07233900	1.85014500
H	4.24682700	4.44984000	2.90140100
H	6.64378200	1.78910100	0.51115400
H	6.50745900	3.42360600	2.35949800
C	4.61512600	0.55714700	-0.77437800
H	4.18635500	2.62669000	-0.55723400

TfOH

S	-0.84178400	0.14245300	0.07926100
O	-1.24205200	-0.24155200	1.41134400
O	-1.20033500	1.38644900	-0.53879300
C	0.98957800	-0.00675500	-0.00099100
F	1.33427600	-1.23808300	0.35050000
F	1.52069100	0.86347500	0.83936700
F	1.40478400	0.23912700	-1.22951600
O	-1.22753400	-1.04243800	-0.93379800
H	-1.44731300	-1.83905600	-0.41540500

intD_s0

C	-3.27074200	-2.46303800	0.54311600
C	-1.87492600	-2.25967700	0.59833500
C	-0.96486900	-3.31165700	0.65886200
C	-1.45196600	-4.61363400	0.70081700

C	-2.82908500	-4.83259900	0.73115600
C	-3.73517400	-3.77793600	0.66563900
C	-4.07214300	-1.22398100	0.43727900
C	-3.22592700	-0.01574100	0.46232000
H	0.10320200	-3.11557300	0.66617700
H	-0.76217000	-5.44968900	0.72587000
H	-3.20802300	-5.84662400	0.81211100
H	-4.79403400	-3.98788700	0.74221000
C	-0.80696000	-0.04040900	2.22528200
C	-0.24396900	-1.00524000	3.06487600
C	-0.67582400	1.32145600	2.52960300
C	0.48956200	-0.60521300	4.17672000
H	-0.35555000	-2.06107600	2.84316700
C	0.06381900	1.71275300	3.63705700
H	-1.10915200	2.08025200	1.88706200
C	0.65803800	0.74919900	4.45092600
H	0.94433300	-1.35373200	4.81738500
H	0.20148600	2.76897900	3.83910900
H	1.25685800	1.05765300	5.30172300
P	-1.51172900	-0.51532200	0.64588900
O	-0.61420700	-0.00755100	-0.52589200
S	1.43655600	-2.35457900	-1.98037300
O	1.12808800	-1.26510300	-2.91987900
O	0.62832600	-3.55016200	-1.96318200
O	1.63066200	-1.70956200	-0.60869600
C	3.14610400	-2.87498400	-2.38655600
F	3.95713400	-1.83520800	-2.25148500
F	3.18432600	-3.30855200	-3.63807500
F	3.52739000	-3.84601200	-1.57109900
S	3.40510400	-0.01947900	1.33328900
O	4.04303000	0.65551900	2.43039100
O	3.92392900	0.06837800	-0.03758700

O	1.89182000	0.29190900	1.22341000
C	3.42563400	-1.83696500	1.76932000
F	2.20340000	-2.27076900	2.05205900
F	4.19398500	-2.00123800	2.83421900
F	3.92084200	-2.53051700	0.76058200
O	1.18347600	4.08195900	1.62907300
O	0.38014200	2.43142400	-0.05940000
O	2.74225500	2.88204700	0.03218200
C	1.12928800	4.73757800	-0.92491000
F	1.33090700	4.20287000	-2.12788900
F	1.94628300	5.76273300	-0.75680000
F	-0.12999300	5.15581400	-0.84755500
Bi	1.40399900	0.54570700	-0.85392900
S	1.43168400	3.45827100	0.35069500
C	-3.66148900	1.20454000	0.05104800
C	-2.81120500	2.27653900	-0.44329600
C	-5.49110000	2.06532800	-1.40066600
C	-3.28381300	3.08109200	-1.42188300
H	-1.77081100	2.34441300	-0.14394000
C	-4.60874300	2.87889000	-1.99920700
H	-6.48493200	1.89971000	-1.80542800
H	-2.62841100	3.83328800	-1.85035300
H	-4.85865700	3.38791900	-2.92448000
C	-5.41071800	-1.08511000	0.20490000
C	-6.29184600	-2.10982500	-0.30428000
C	-7.59991300	-1.86375000	-0.54461500
H	-5.87057000	-3.06071400	-0.60496100
C	-7.44524200	0.45036800	0.17012900
C	-8.19511100	-0.56247000	-0.28368200
H	-8.21800900	-2.63777000	-0.98829000
H	-7.87961900	1.43347000	0.33989100
H	-9.25133500	-0.41986800	-0.48889200

C	-5.99131700	0.28963000	0.49484800
H	-5.89919900	0.39596300	1.58758500
C	-5.14073600	1.47096900	-0.05858400
H	-5.33124600	2.29042400	0.65771600

H_2

H	0.00000000	0.00000000	0.36847500
H	0.00000000	0.00000000	-0.36847500

intE

C	-3.22197000	-2.52510400	0.42056600
C	-1.82683200	-2.31039300	0.46399400
C	-0.91379100	-3.32931400	0.68797000
C	-1.39744300	-4.61968100	0.91049500
C	-2.76857400	-4.83514600	0.96989200
C	-3.68167000	-3.80150400	0.74134500
C	-4.00022600	-1.28864800	0.08471800
C	-3.23490900	-0.14988300	0.03696000
H	0.15169600	-3.12128100	0.69535600
H	-0.70442800	-5.43965400	1.06194100
H	-3.14765200	-5.82576600	1.20005100
H	-4.73597300	-4.01125100	0.85311300
C	-0.99439600	0.00848800	2.02780900
C	-0.54916700	-0.91896800	2.97660100
C	-0.88336100	1.38204000	2.28861200
C	0.03835400	-0.47391800	4.15538800
H	-0.63985800	-1.98347300	2.79433800
C	-0.28970100	1.81695200	3.46592200
H	-1.21737500	2.11782100	1.56797400
C	0.18222100	0.89011300	4.39289400
H	0.39781600	-1.19690900	4.88028000
H	-0.16887300	2.88084200	3.63535200

H	0.66628400	1.23311500	5.30158200
P	-1.52186200	-0.55049300	0.40228600
O	-0.56570300	-0.04297100	-0.71641700
S	1.57297400	-2.40985100	-1.91128500
O	1.36759600	-1.35299200	-2.91424400
O	0.73856800	-3.58759100	-1.91586400
O	1.66530900	-1.71453400	-0.55371000
C	3.29934000	-2.97711700	-2.14736500
F	4.11678100	-1.94669400	-1.98391500
F	3.43694400	-3.46462400	-3.37132300
F	3.58706000	-3.91855600	-1.26152500
S	3.27089800	0.05372600	1.45751400
O	3.82393800	0.77651700	2.56946400
O	3.89064900	0.09086200	0.12523200
O	1.76950900	0.34915300	1.21640100
C	3.26166400	-1.74432600	1.96710100
F	2.02082300	-2.16769700	2.17630000
F	3.94910900	-1.86246800	3.09121000
F	3.82943000	-2.47752400	1.02712600
O	1.02458800	4.05990500	1.49200500
O	0.35254000	2.43222300	-0.27738400
O	2.69745400	2.86883200	0.00836700
C	1.18672100	4.75918600	-1.04228500
F	1.43660100	4.23718900	-2.24011800
F	2.03450000	5.74552500	-0.80591300
F	-0.05570300	5.22964600	-1.02535700
Bi	1.47408300	0.53303500	-0.90647100
S	1.36755400	3.45251900	0.22803200
C	-3.76578300	1.15521700	-0.22396000
C	-5.17149200	1.29876800	-0.30294300
C	-2.92844300	2.27985600	-0.39080600
C	-5.69345300	2.60146600	-0.45744400

C	-3.46734100	3.53487600	-0.55970500
H	-1.85074800	2.15282800	-0.41580000
C	-4.86298300	3.69640900	-0.57121000
H	-6.76423700	2.75911800	-0.50052400
H	-2.81087700	4.38918600	-0.68820400
H	-5.29292800	4.68546700	-0.69102700
C	-5.41649000	-1.19017000	-0.20191500
C	-6.22897300	-2.32340600	-0.43627900
C	-6.00525300	0.10272900	-0.30339500
C	-7.58412400	-2.20582400	-0.64874600
H	-5.77379000	-3.29983100	-0.51204800
C	-7.40204500	0.18749100	-0.48857900
C	-8.18224900	-0.93757400	-0.63796100
H	-8.18177200	-3.09154100	-0.83657900
H	-7.87975000	1.15786200	-0.54111500
H	-9.25261600	-0.83737300	-0.78525200

Photoluminescence Properties

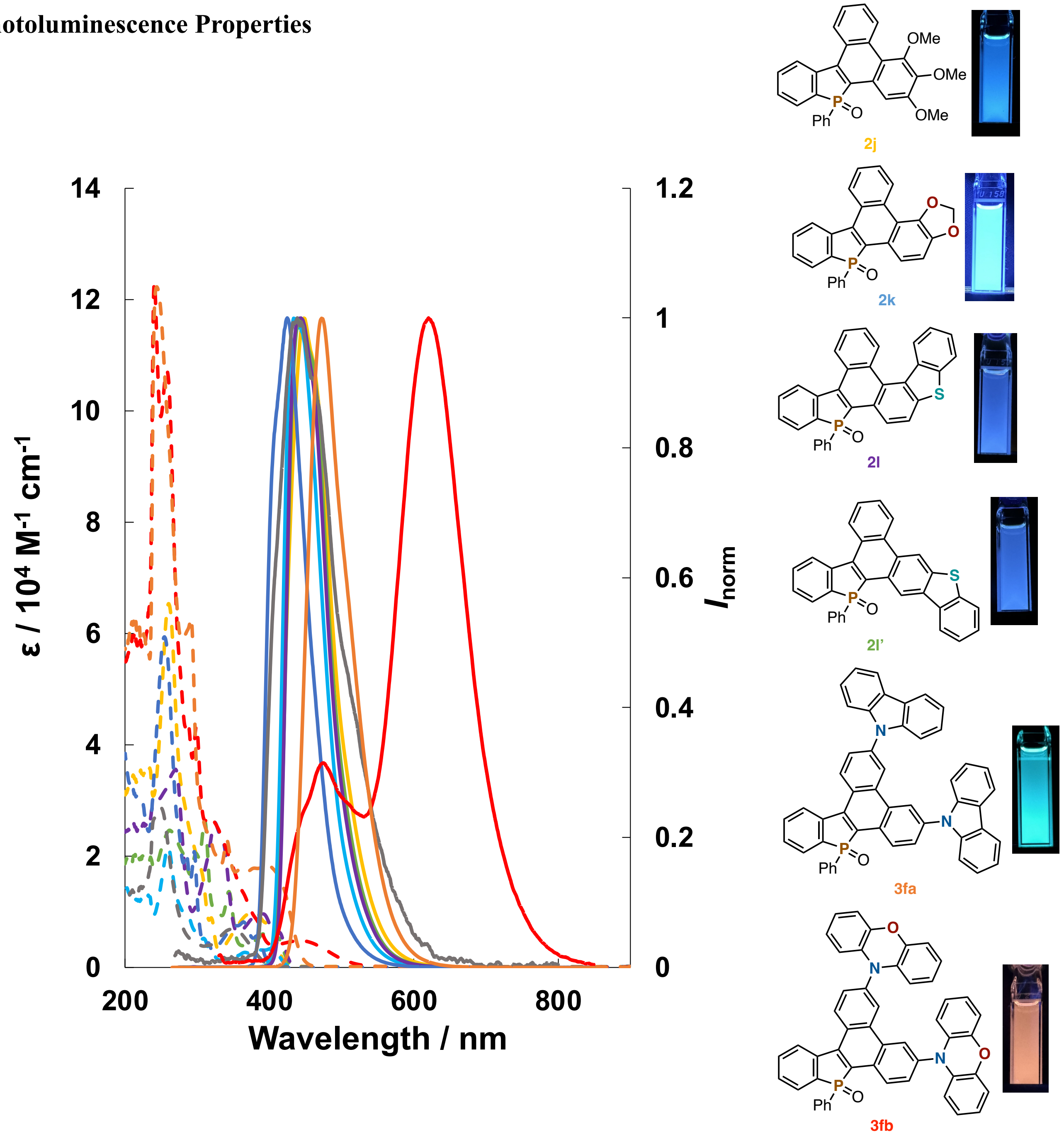

Figure 13. UV-vis absorption spectra (dotted lines) and emission (solid lines) spectra of **1a**, **2a**, **2j**, **2k**, **2l**, **2l'**, **3fa**, and **3fb** in $CHCl_3$ (1.0×10^{-5} M), and fluorescence images of **2j**, **2k**, **2l**, **2l'**, **3fa**, and **3fb.**

Table S7. Optical properties of **1a**, **2a**, **2j**, **2k**, **2l**, **2l'**, **3fa**, and **3fb**.[a]

compd	λ_{abs} (nm)	λ_{em} (nm)[b]	Φ_F[c]	$\Delta\nu$ /cm^{-1}[d]
1a	249, 342	438	0.11	6409
2a[e]	254, 315, 364, 382	425	0.56	2649
2j	260, 379	448	0.58	4064
2k	260, 403	434	0.75	1772
2l	212, 270, 324, 392	443	0.16	2937
2l'	262, 310, 344, 391, 412	432	0.16	1124
3fa	244, 291, 341, 396	473	>0.99	4111
3fb	240, 258, 298, 317, 440	620	0.04	6598

[a] Measured in 1.0 × 10^{-5} M solution of $CHCl_3$. [b] Excited at **1a** (249 nm), **2a** (254 nm), **2j** (260 nm), **2k** (260 nm), **2l** (270 nm), **2l'** (262 nm), **3fa** (244 nm), and **3fb** (298 nm). [c]Absolute fluorescence quantum yields. [d]Stokes shifts. [e]The optical data of **2a** was taken from ref. S2.

Electrochemical Properties

The IUPAC convention was used to report the CV and DPV data. The CV and DPV were recorded in MeCN (0.01 M, degassed by N_2 gas bubbling) containing 0.1 M n-Bu_4NPF_6 with a Pt working electrode, a Pt counter electrode, and a Ag/Ag+ reference electrode. The measurements were performed at room temperature.

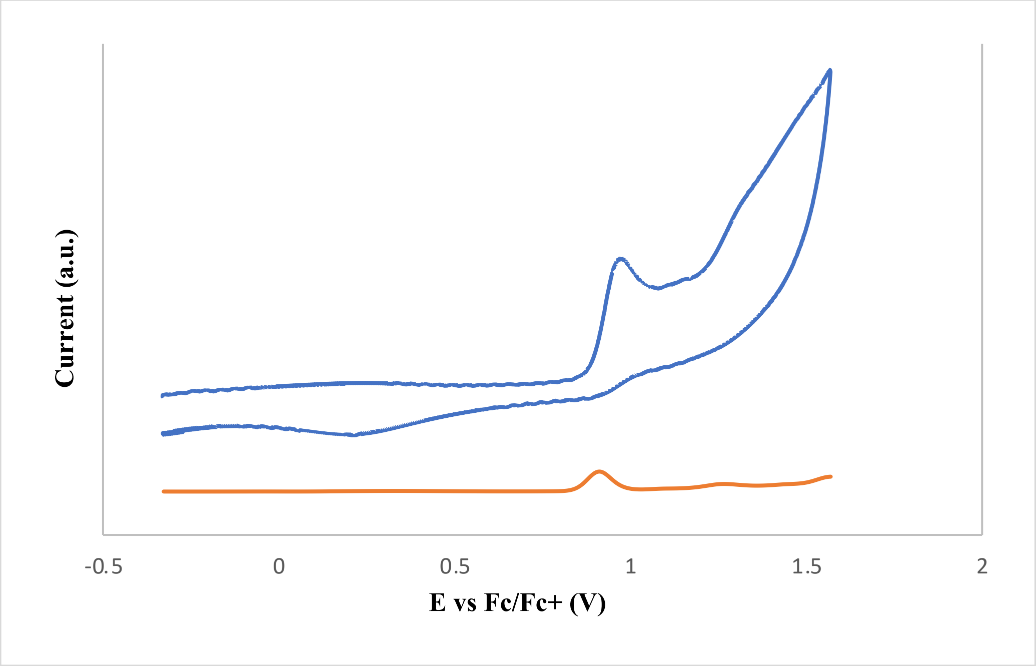

Figure S14. Cyclic voltammograms (CV; blue line, from 0 V to 1.5 V then back to 0 V) and differential pulse voltammograms (DPV; orange line) of **2j** in MeCN containing 0.1 M n-Bu_4NPF_6 at a scan rate of 0.10 V/s.

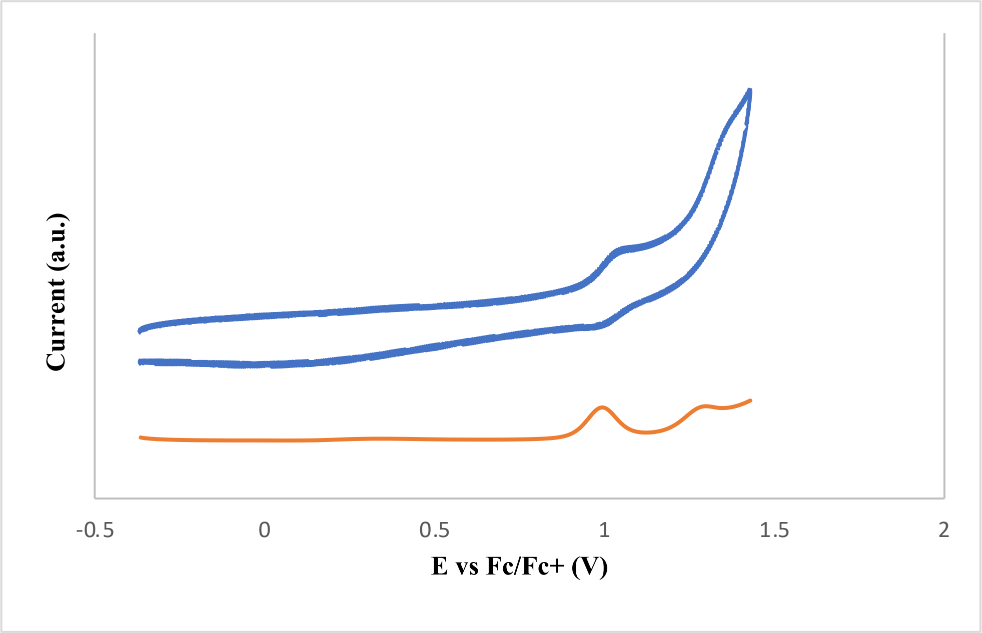

Figure S15. Cyclic voltammograms (CV; blue line, from 0 V to 1.4 V then back to 0 V) and differential pulse voltammograms (DPV; orange line) of **2k** in MeCN containing 0.1 M n-Bu_4NPF_6 at a scan rate of 0.10 V/s.

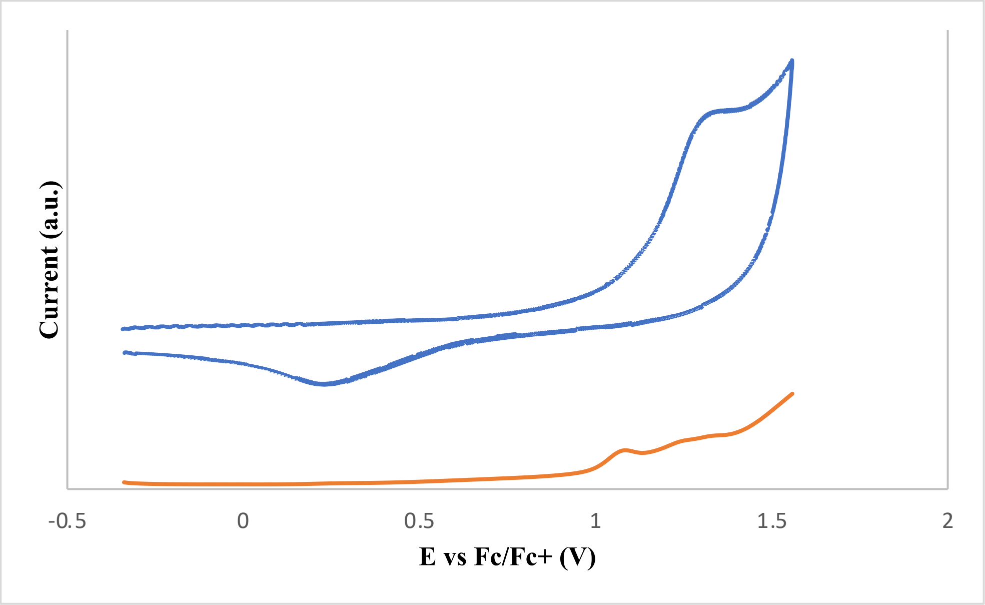

Figure S16. Cyclic voltammograms (CV; blue line, from 0 V to 1.5 V then back to 0 V) and differential pulse voltammograms (DPV; orange line) of **2l** in MeCN containing 0.1 M n-Bu_4NPF_6 at a scan rate of 0.10 V/s.

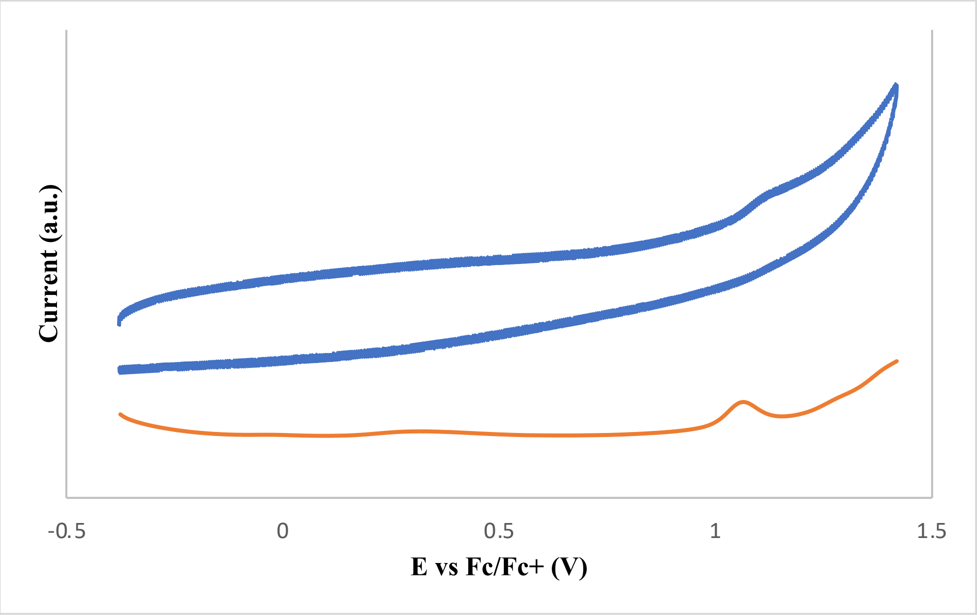

Figure S17. Cyclic voltammograms (CV; blue line, from 0 V to 1.4 V then back to 0 V) and differential pulse voltammograms (DPV; orange line) of **2l’** in MeCN containing 0.1 M n-Bu_4NPF_6 at a scan rate of 0.10 V/s.

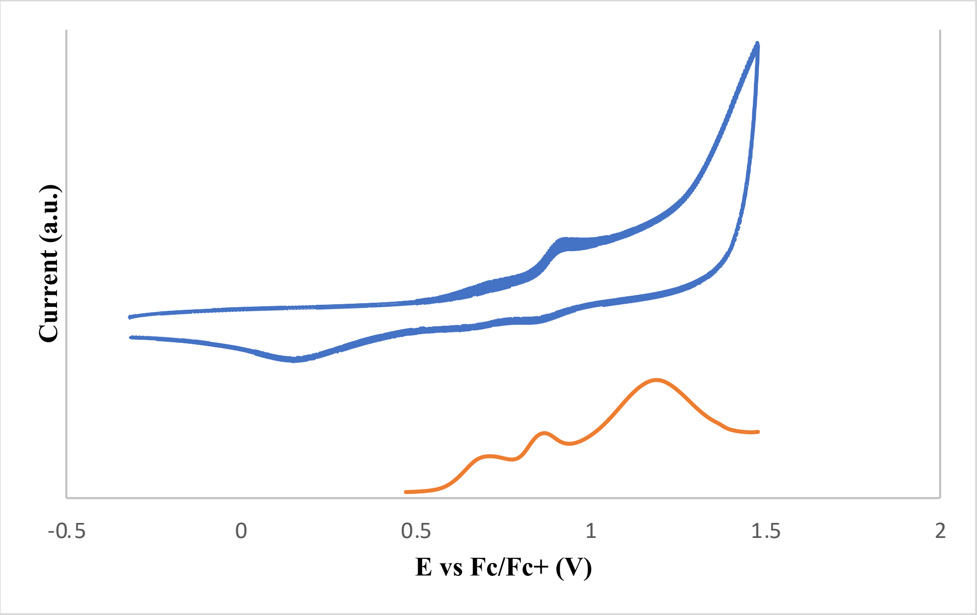

Figure S18. Cyclic voltammograms (CV; blue line, from 0 V to 1.5 V then back to 0 V) and differential pulse voltammograms (DPV; orange line) of **3fa** in MeCN containing 0.1 M n-Bu_4NPF_6 at a scan rate of 0.10 V/s.

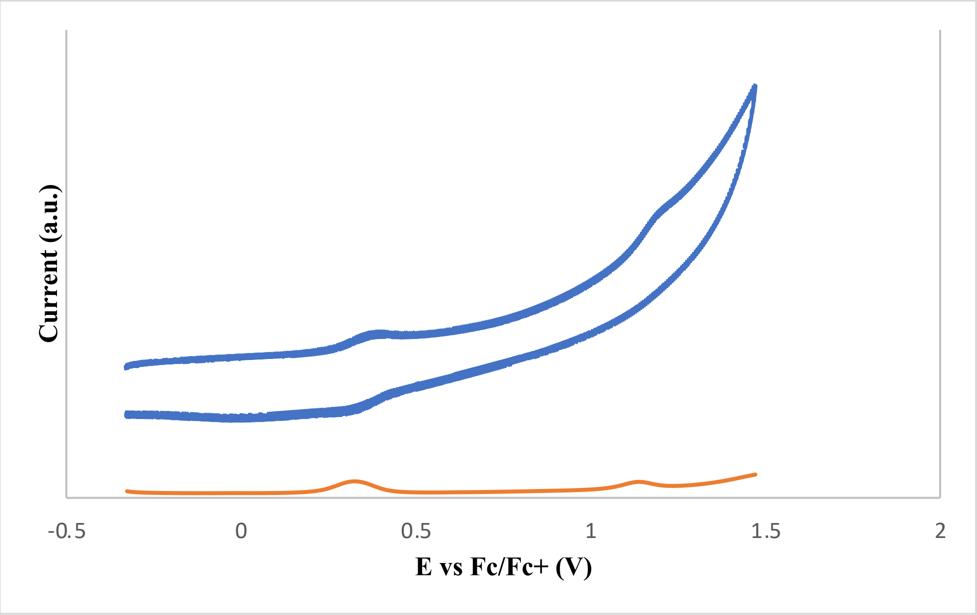

Figure S19. Cyclic voltammograms (CV; blue line, from 0 V to 1.5 V then back to 0 V) and differential pulse voltammograms (DPV; orange line) of **3fb** in MeCN containing 0.1 M n-Bu_4NPF_6 at a scan rate of 0.10 V/s.

Table S8. Absorption wavelengths, HOMO-LUMO energy gaps and differential pulse voltammetry data of compounds **2a**, **2j**, **2k**, **2l**, **2l'**, **3fa**, and **3fb.**

compd	λ_{onset}^{abs} (nm)^{a}	E_g^{opt} (eV)^{b}	E_{ox} (V)^{c}	E_{HOMO} (eV)^{d}	E_{LUMO} (eV)^{e}
**2a**^{f}	397	3.12	1.32	–6.12	–3.00
**2j**	422	2.94	0.91	–5.71	–2.77
**2k**	424	2.92	0.99	–5.79	–2.87
**2l**	429	2.89	1.08	–5.88	–2.99
**2l'**	428	2.90	1.07	–5.87	–2.97
**3fa**	445	2.79	0.72	–5.52	–2.73
**3fb**	521	2.38	0.32	–5.12	–2.74

^{a} Measured in $CHCl_3$. ^{b} Determined from the onset of the absorption spectra. ^{c} Performed in MeCN in the presence of Bu_4NPF_6. v = 0.10 V/s. Values determined by DPV, versus Fc/Fc^+. ^{d} The approximation for Fc/Fc^+ level is -4.8 eV versus vacuum: E_{HOMO} = -4.8 - E_{ox}. ^{e} Estimated from E_{HOMO} and E_g^{opt}: $E_{LUMO} = E_{HOMO} + E_g^{opt}$. ^{f} The data of **2a** was taken from ref. S2.

Copies of NMR Spectra

[^{1}H, ^{13}C{^{1}H}, and ^{31}P{^{1}H} NMR Spectra of **2a**]

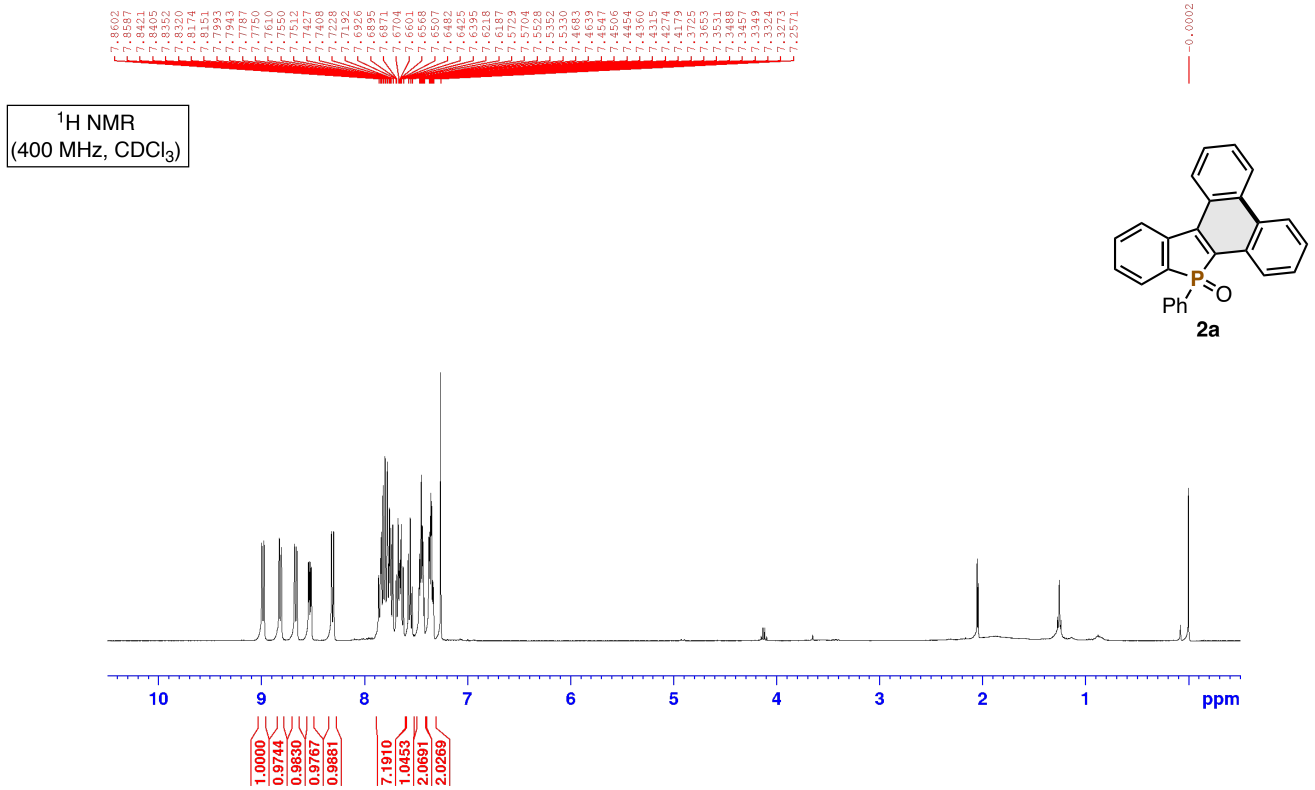

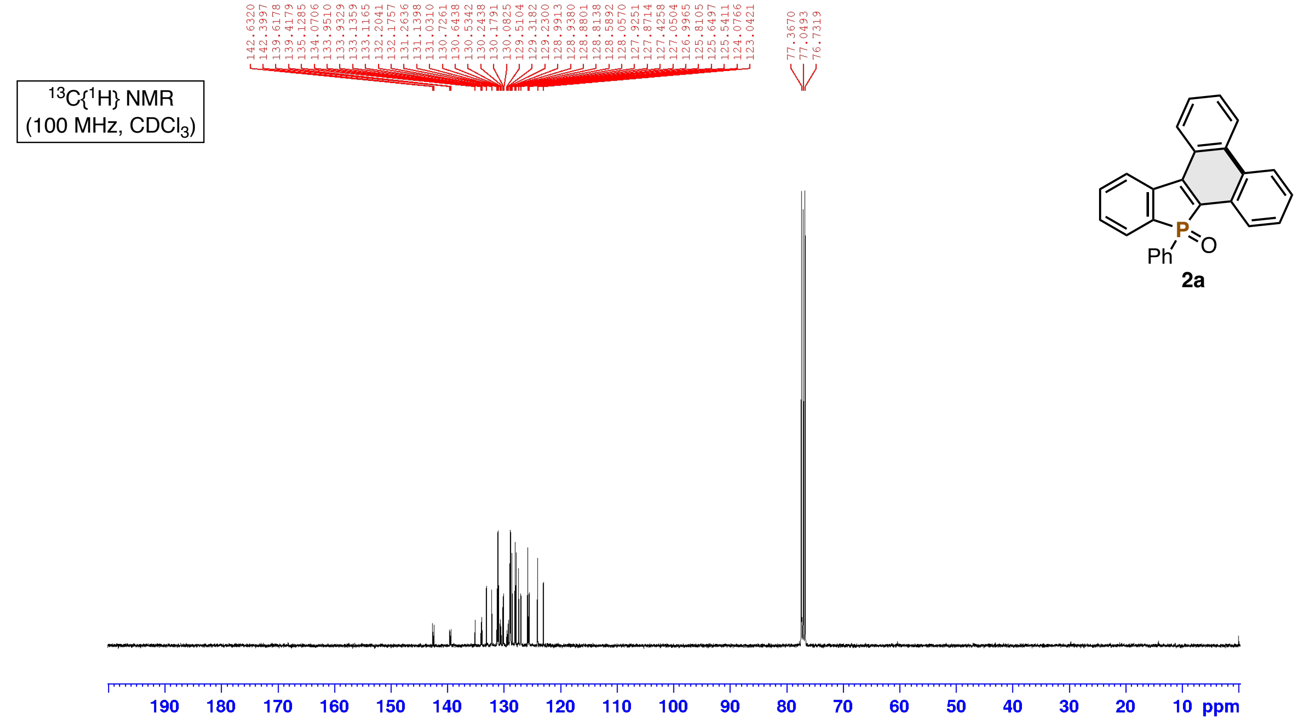

$^{31}P\{^{1}H\}$ NMR
(162 MHz, $CDCl_3$)

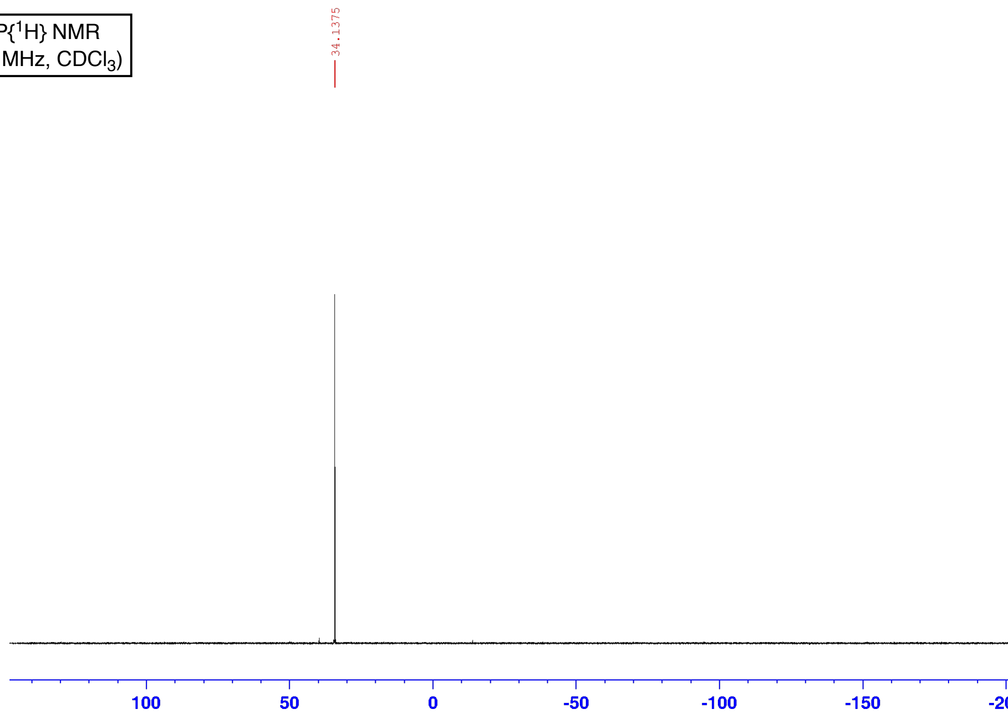

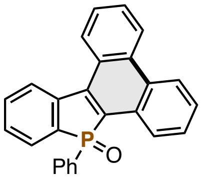

2a

[^{1}H, ^{13}C{^{1}H}, and ^{31}P{^{1}H} NMR Spectra of **2b**]

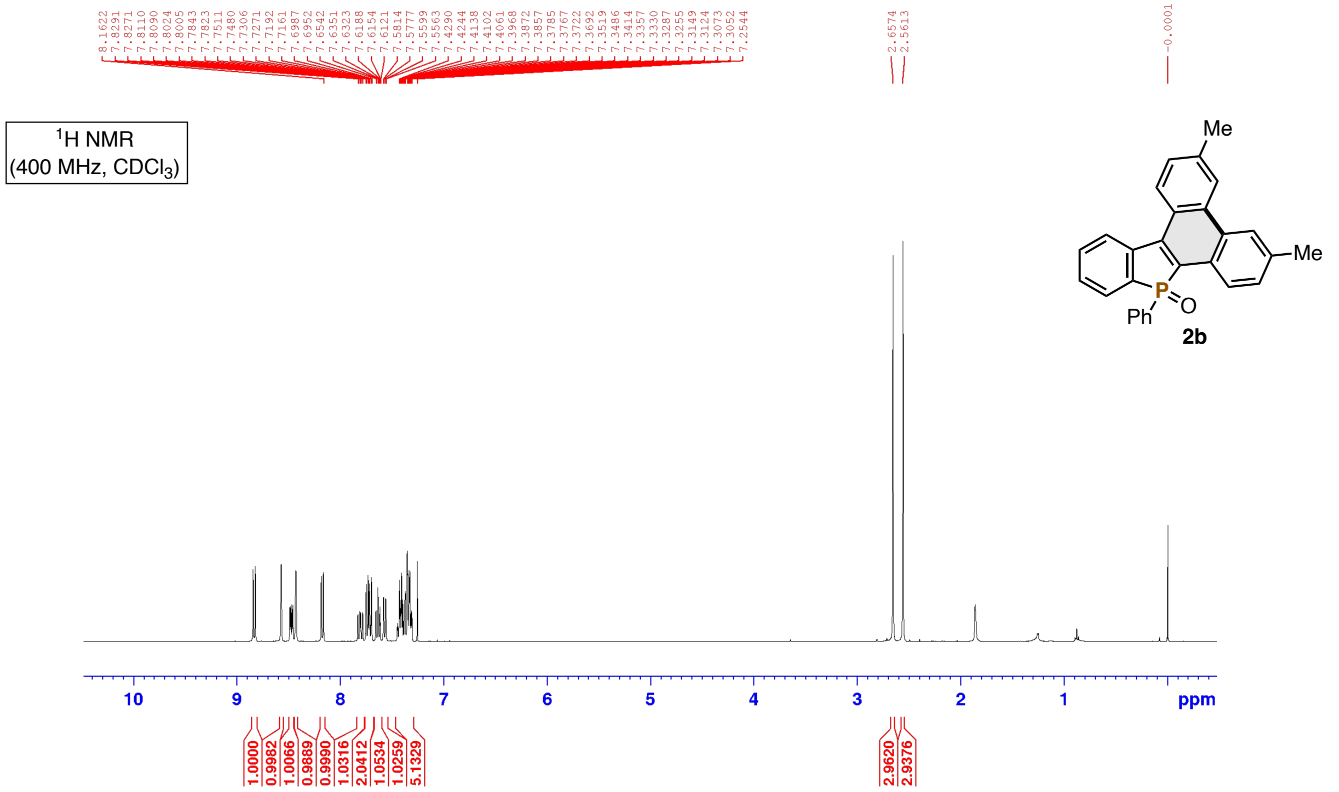

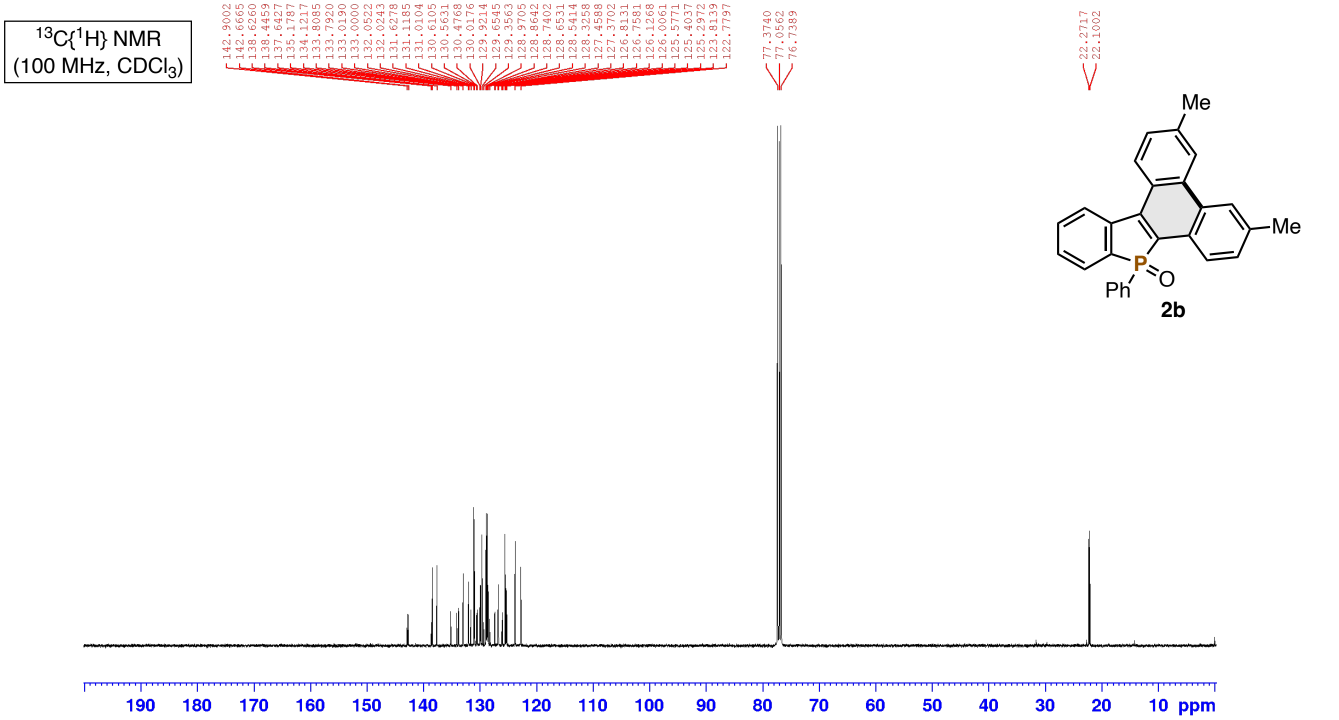

$^{31}P\{^1H\}$ NMR
(162 MHz, $CDCl_3$)

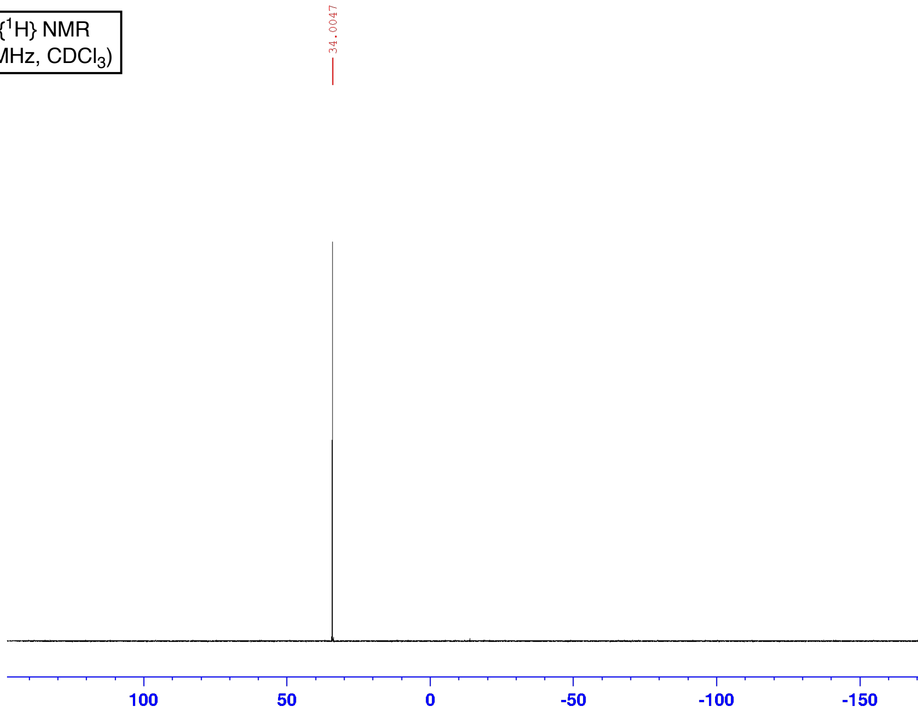

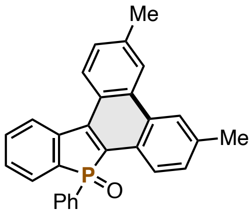

2b

[^{1}H, ^{13}C{^{1}H}, and ^{31}P{^{1}H} NMR Spectra of **2c**]

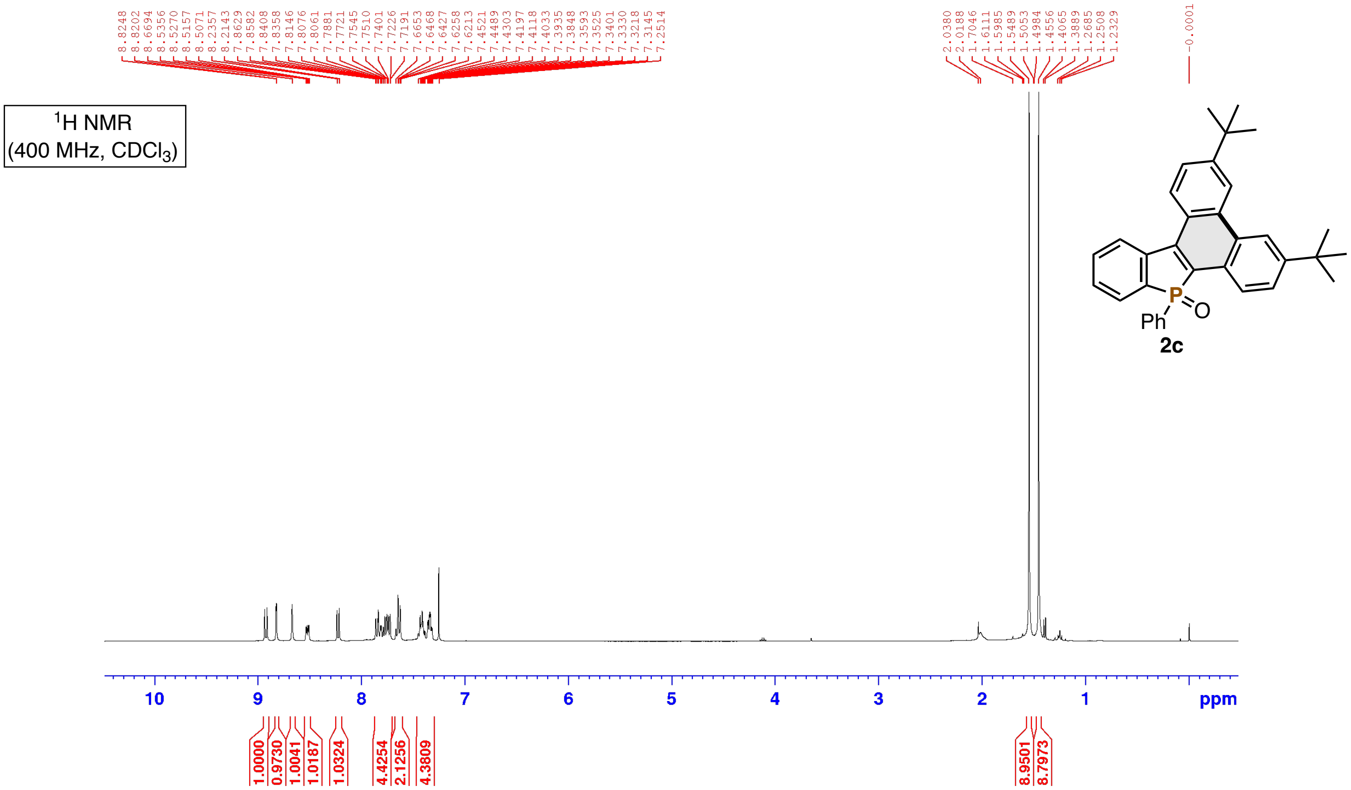

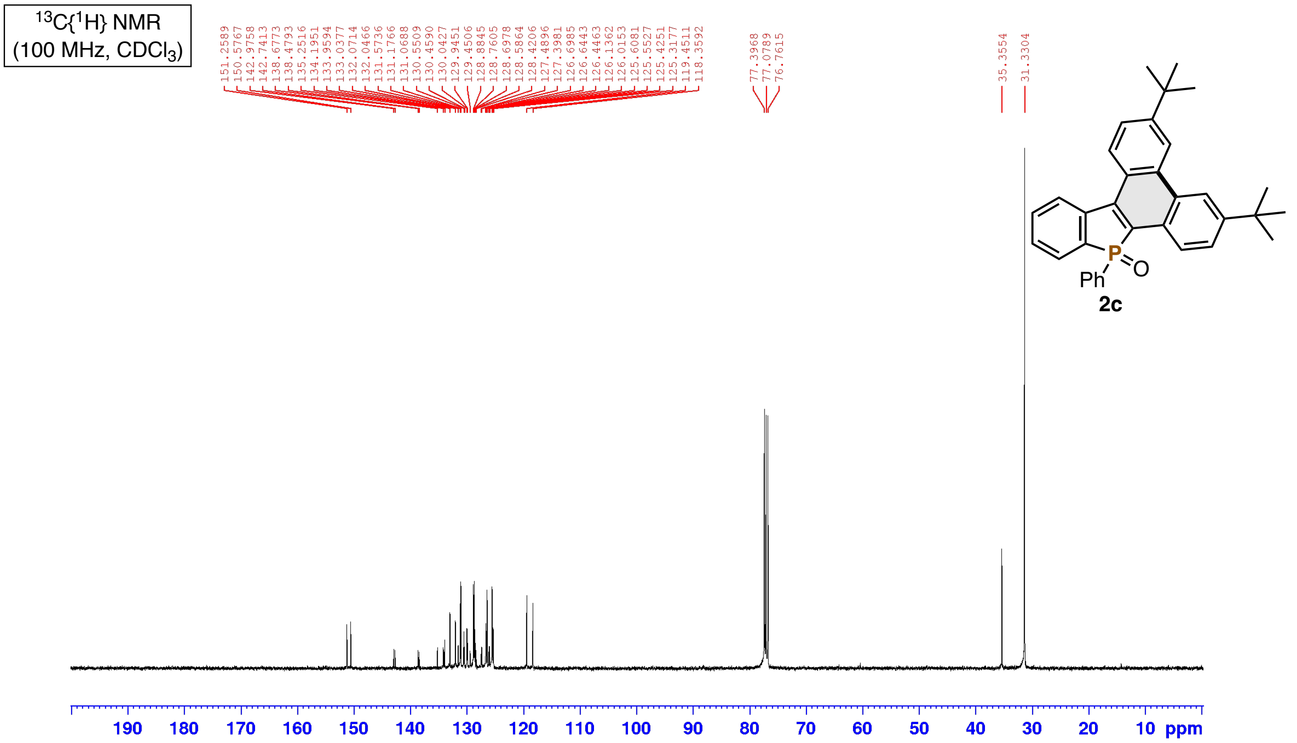

$^{31}P\{^{1}H\}$ NMR
(162 MHz, $CDCl_3$)

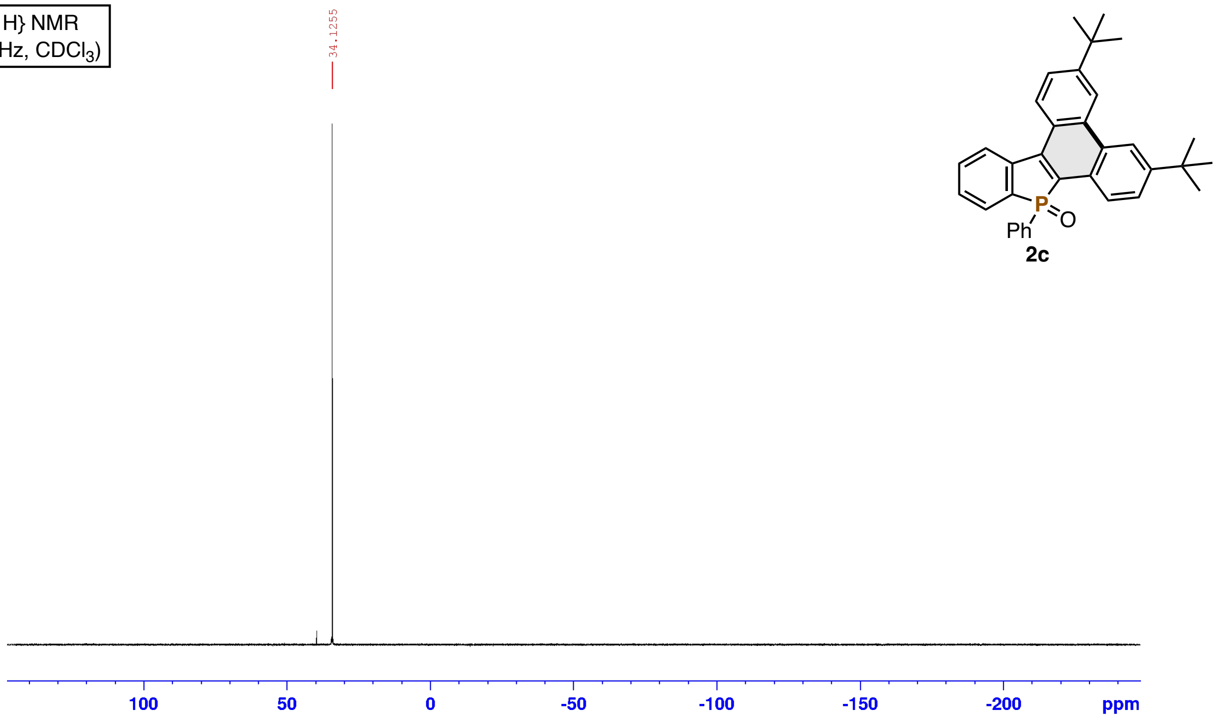

[^{1}H, $^{13}C\{^{1}H\}$, and $^{31}P\{^{1}H\}$ NMR Spectra of **2d**]

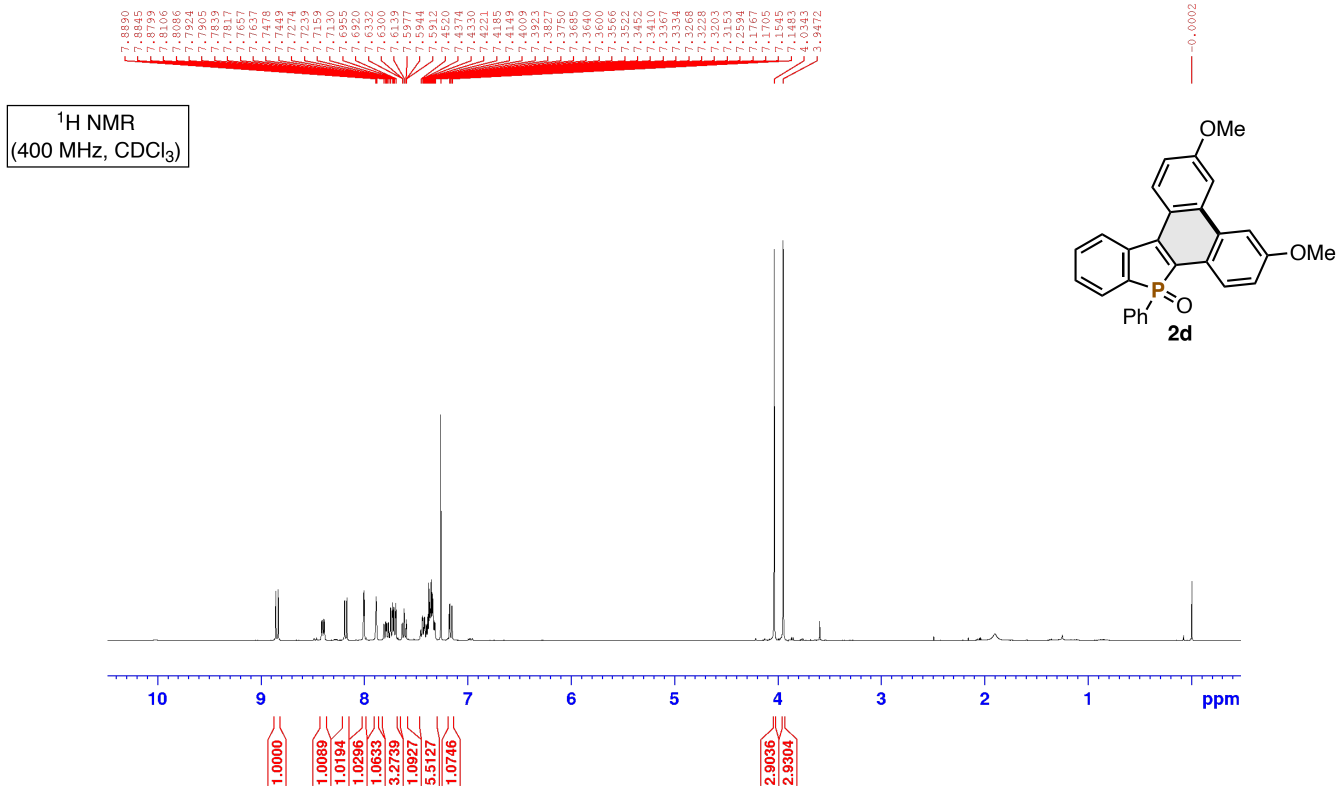

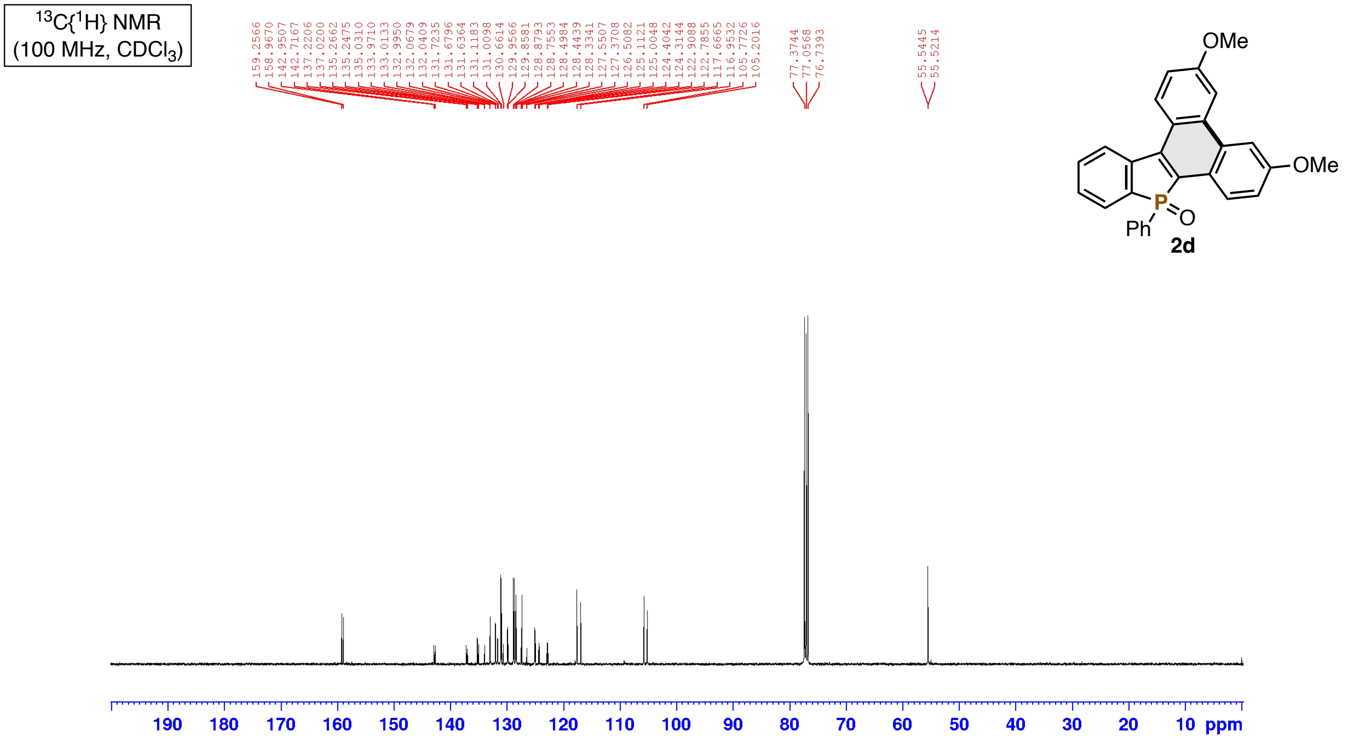

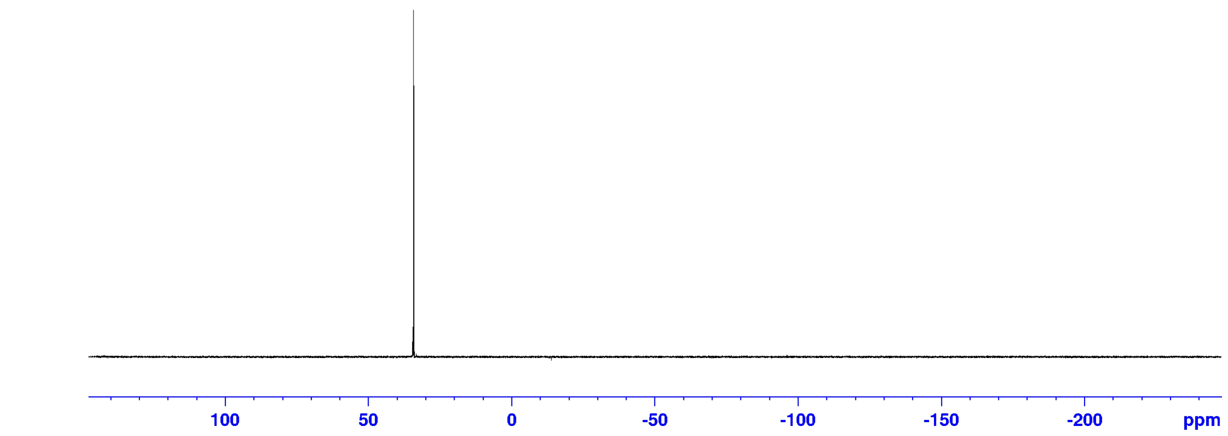
$^{31}P\{^1H\}$ NMR
(162 MHz, $CDCl_3$)
34.0752
100
50
0
-50
-100
-150
-200
ppm

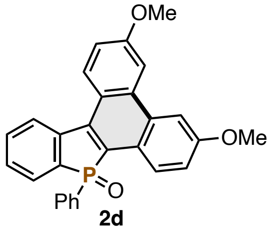
OMe
OMe
P
O
Ph
2d

[^{1}H, $^{13}C\{^{1}H\}$, $^{19}F\{^{1}H\}$, and $^{31}P\{^{1}H\}$ NMR Spectra of **2e**]

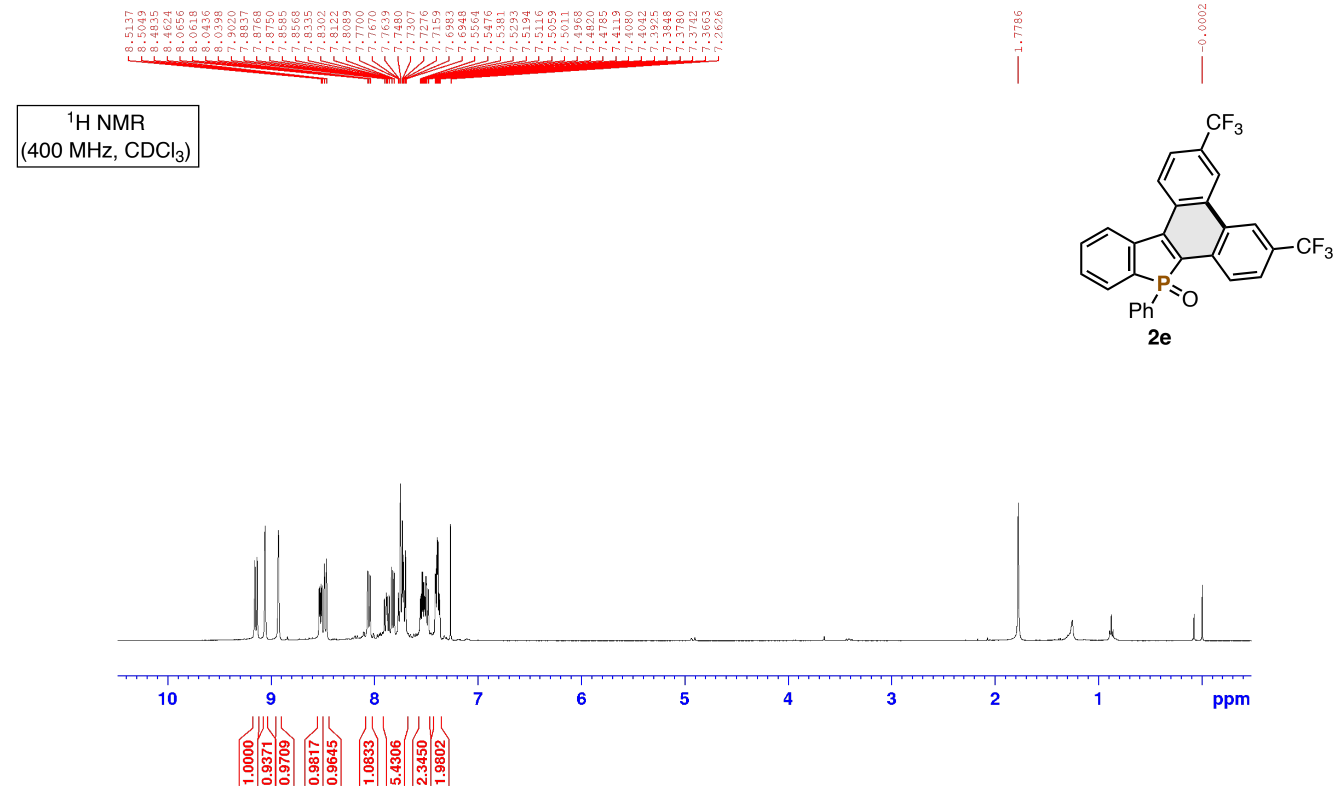

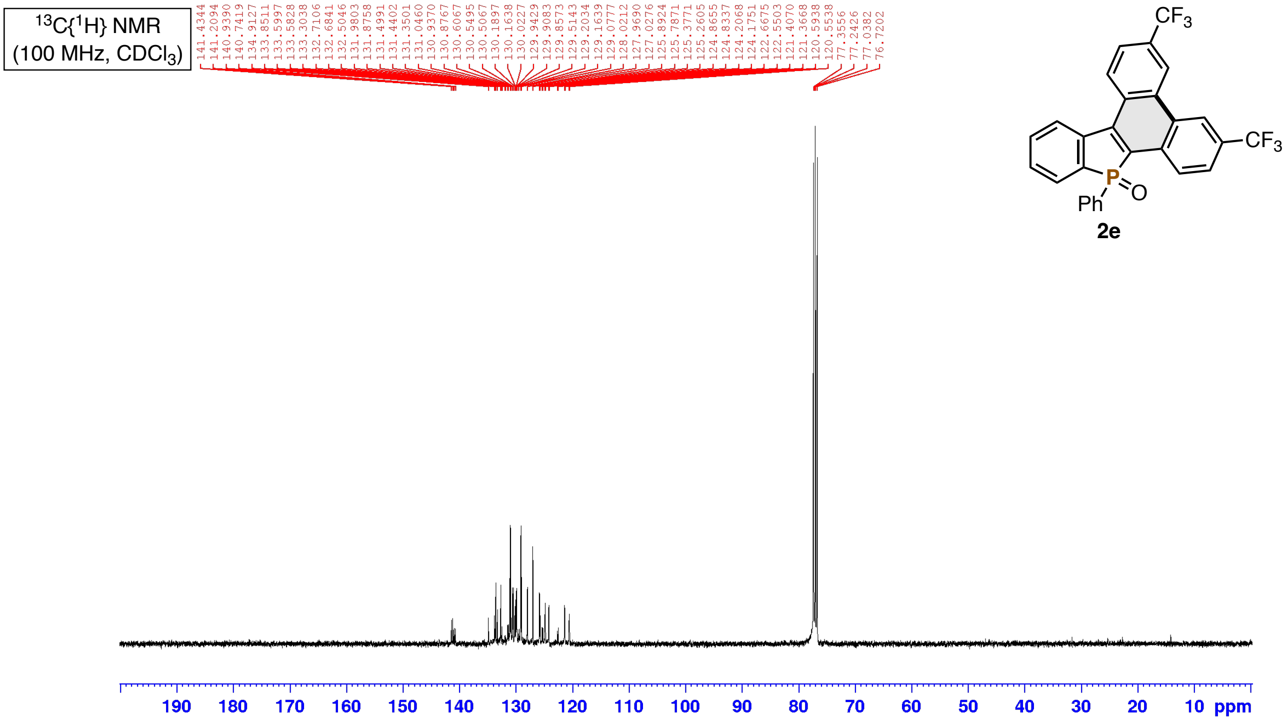

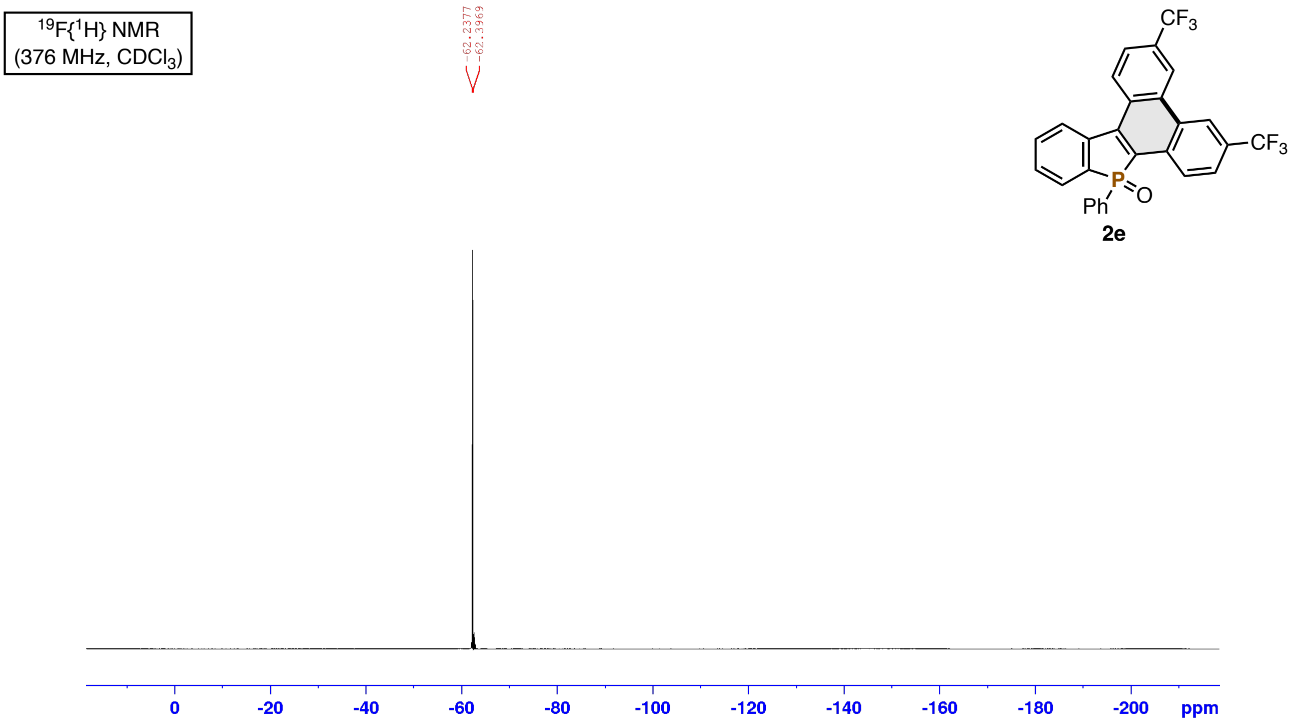

$^{19}F\{^1H\}$ NMR
(376 MHz, $CDCl_3$)
-62.2377
-62.3969
CF_3
CF_3
P
O
Ph
2e
0 -20 -40 -60 -80 -100 -120 -140 -160 -180 -200 ppm

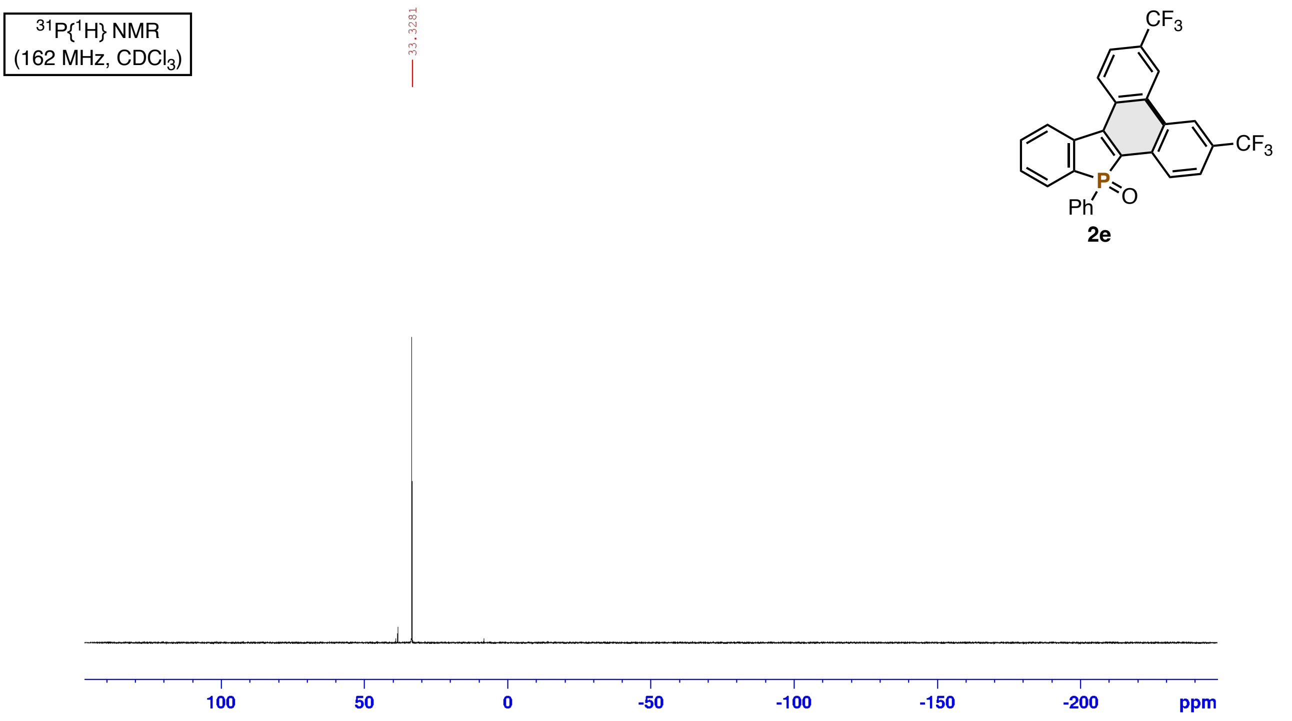

$^{31}P\{^1H\}$ NMR
(162 MHz, $CDCl_3$)
33.3281
CF_3
CF_3
P
O
Ph
2e
100 50 0 -50 -100 -150 -200 ppm

[^{1}H, ^{13}C{^{1}H}, and ^{31}P{^{1}H} NMR Spectra of **2f**]

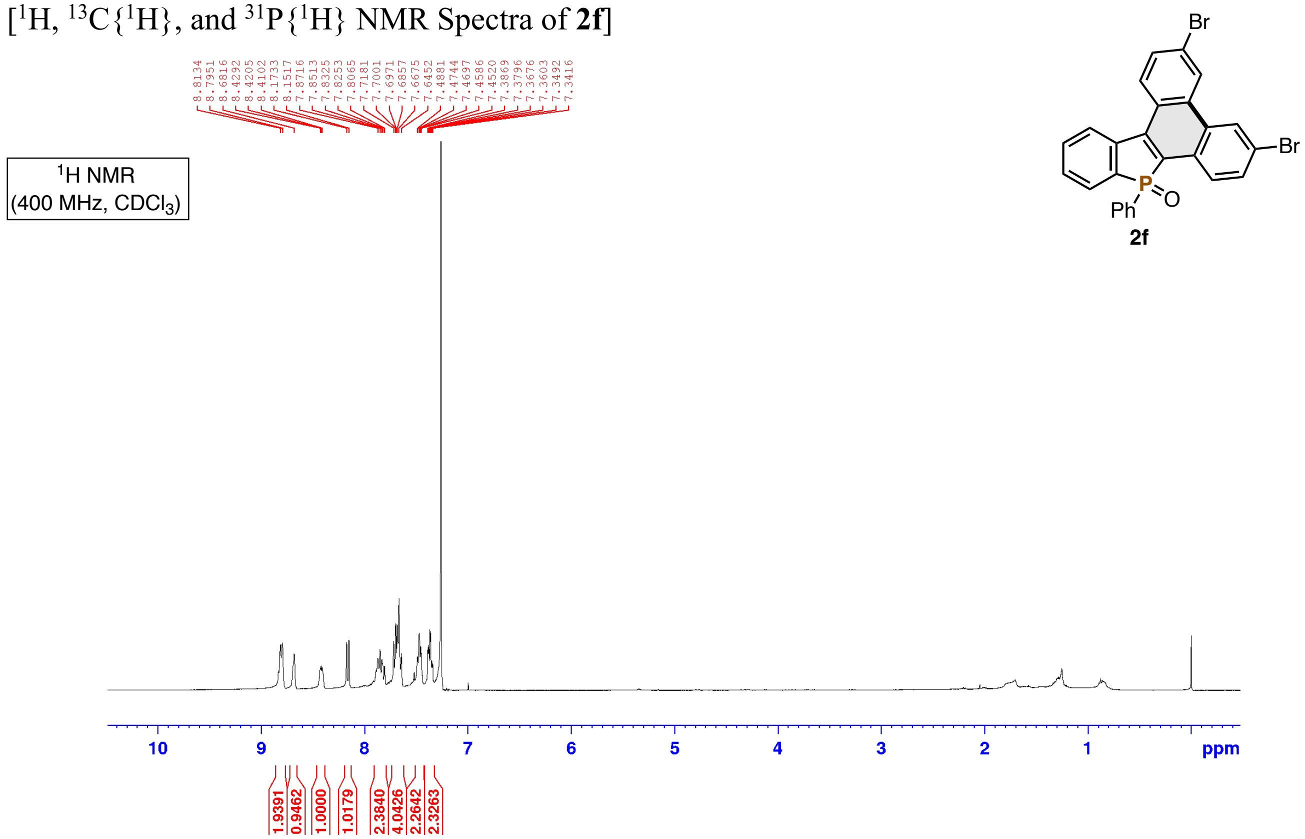

2f

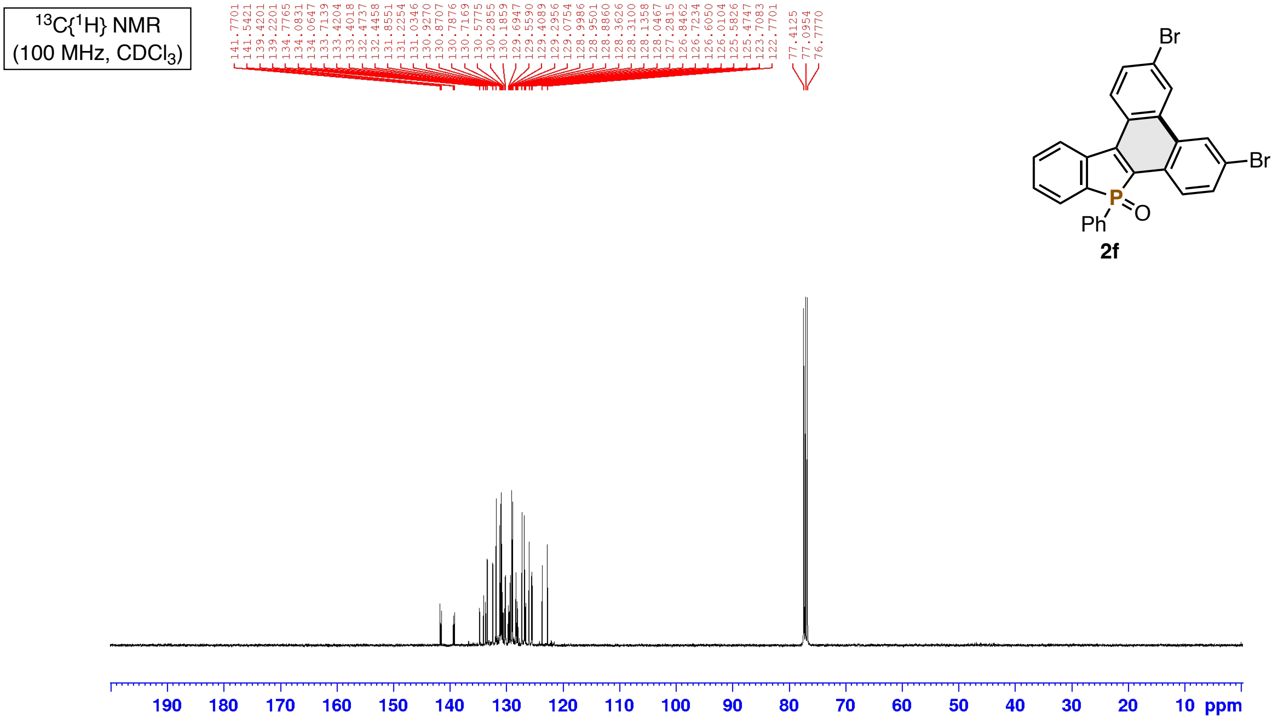

2f

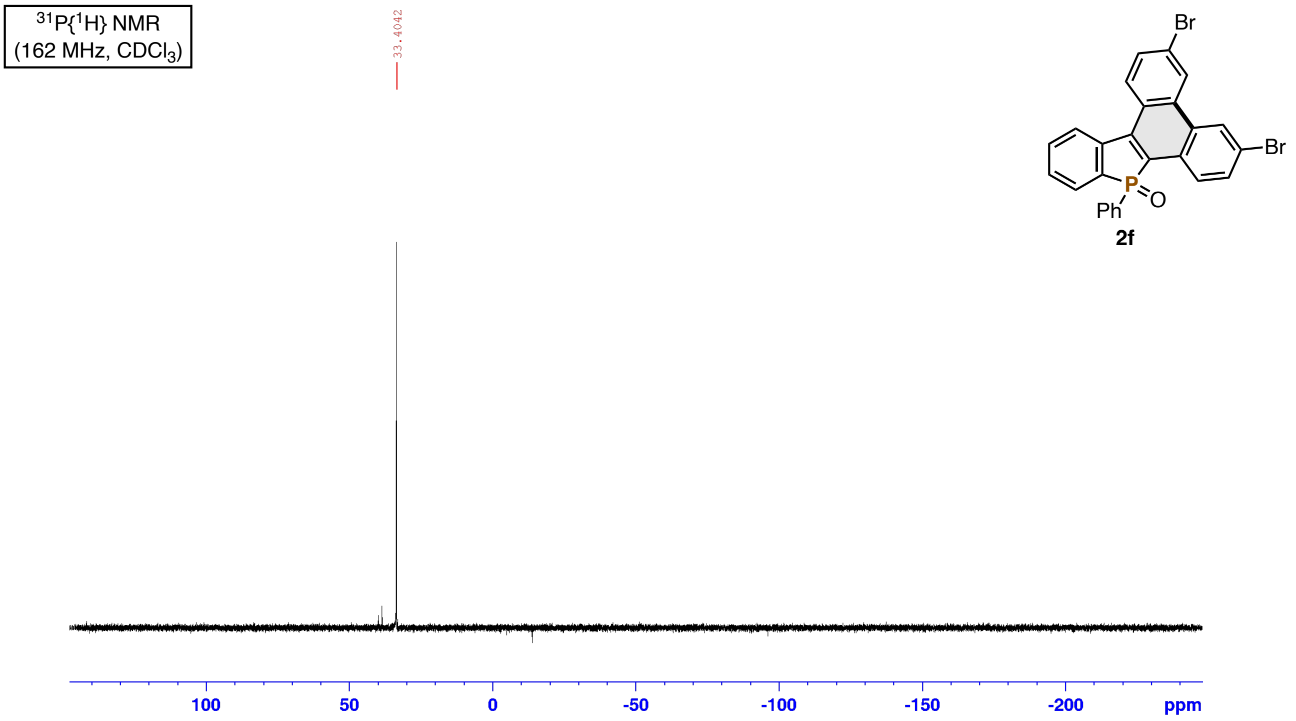

$^{31}P\{^{1}H\}$ NMR
(162 MHz, $CDCl_3$)
33.4042
100
50
0
-50
-100
-150
-200
ppm
Br
Br
P
O
Ph

2f

[^{1}H, $^{13}C\{^{1}H\}$, and $^{31}P\{^{1}H\}$ NMR Spectra of **2g**]

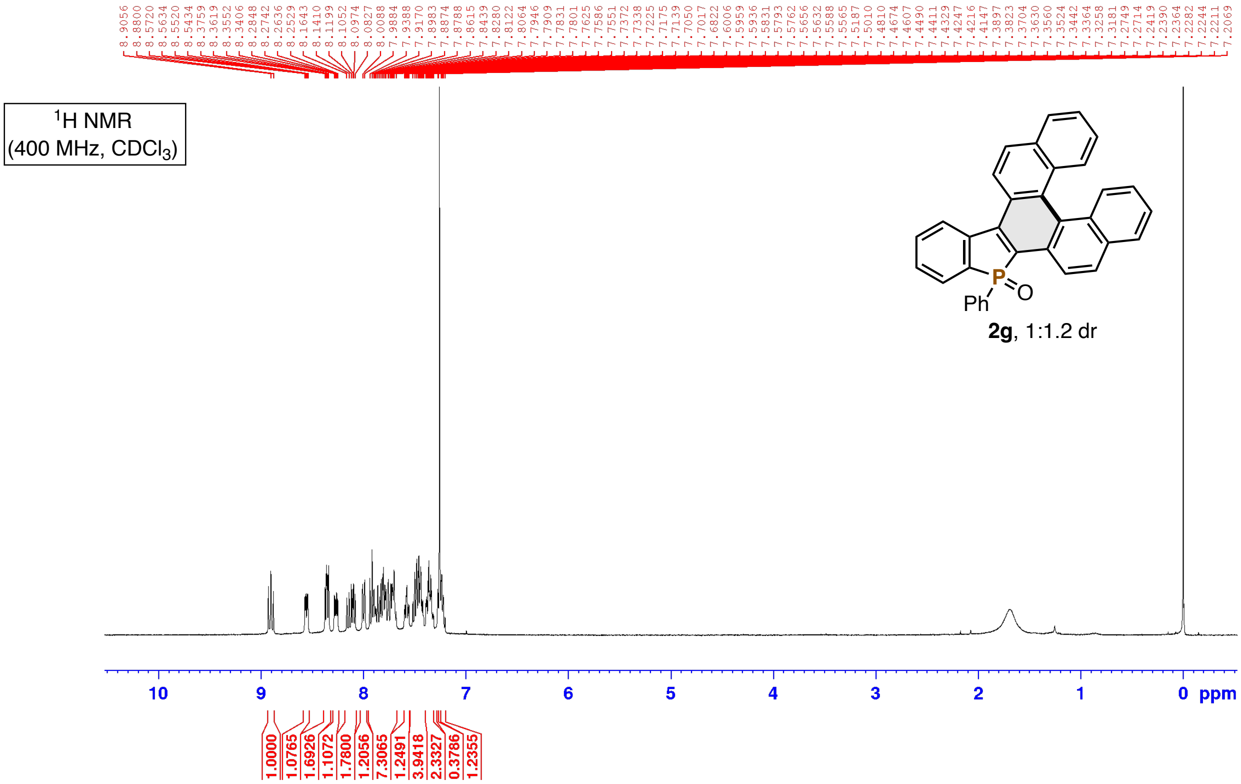

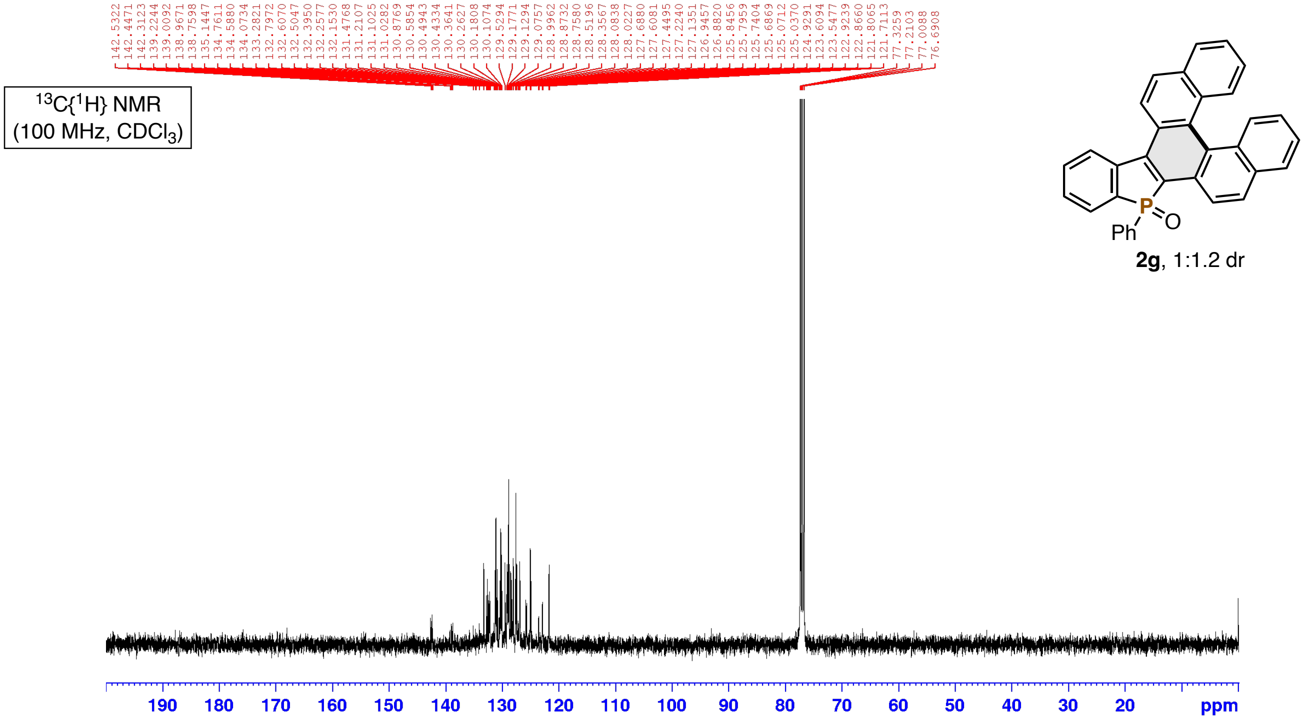

$^{31}P\{^1H\}$ NMR
(162 MHz, $CDCl_3$)

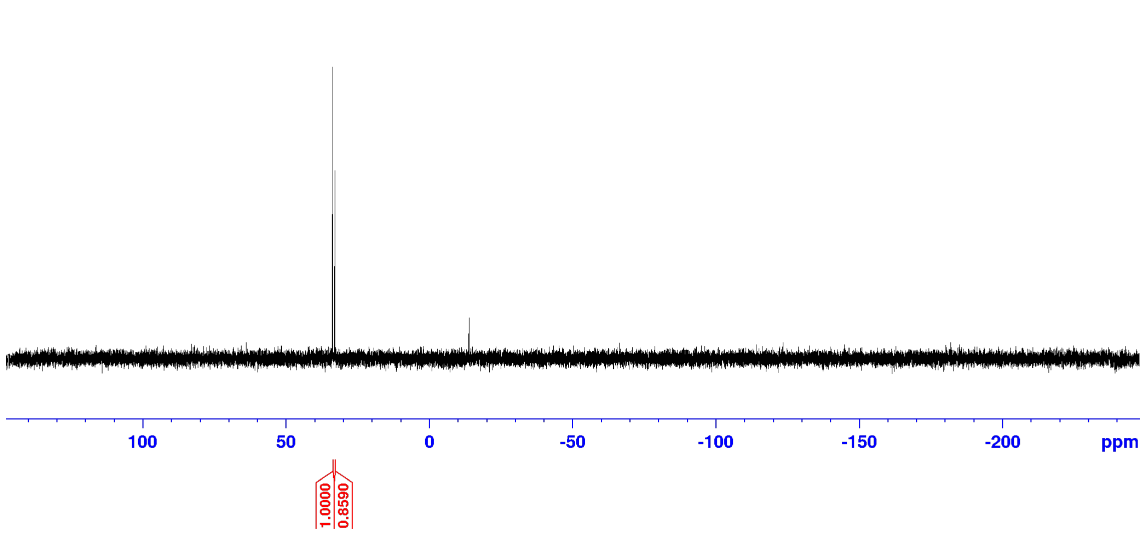

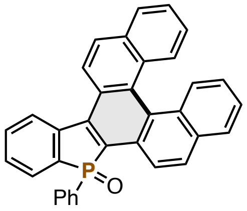

2g, 1:1.2 dr

[^{1}H, ^{13}C{^{1}H}, and ^{31}P{^{1}H} NMR Spectra of **2h**]

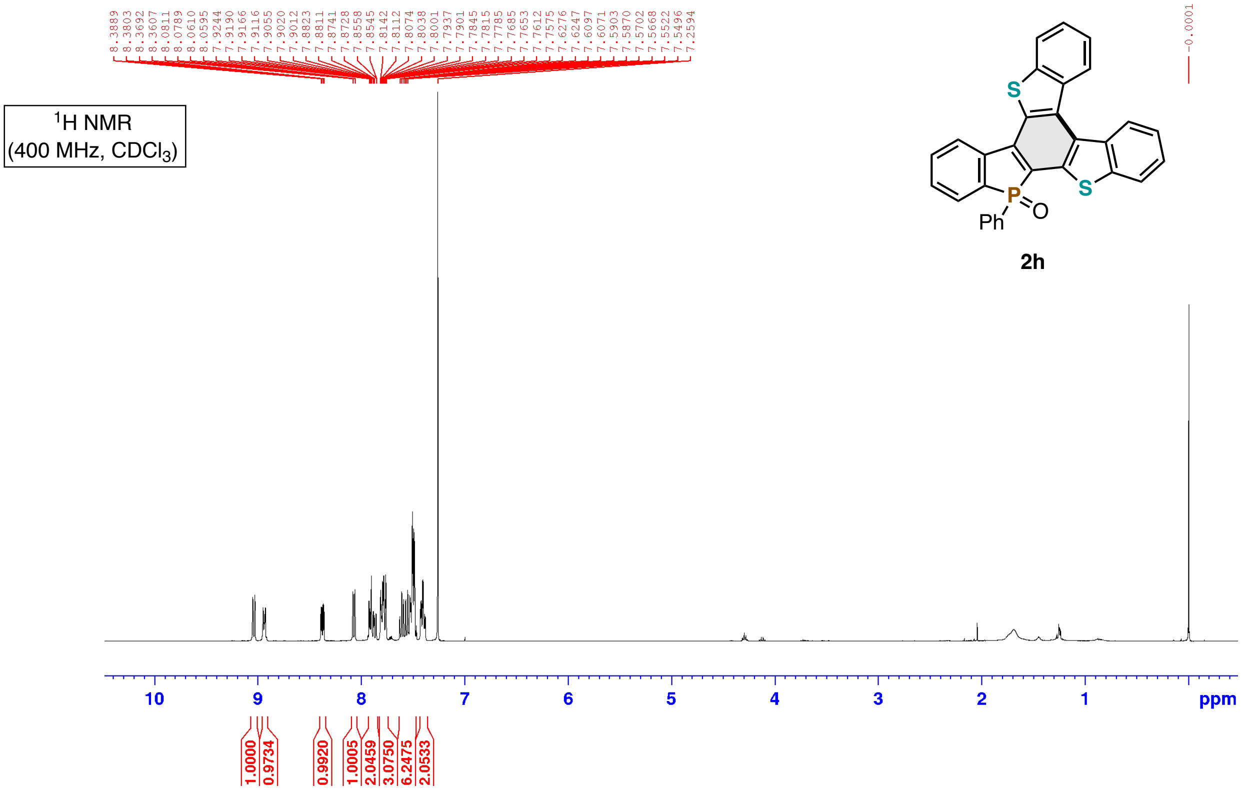

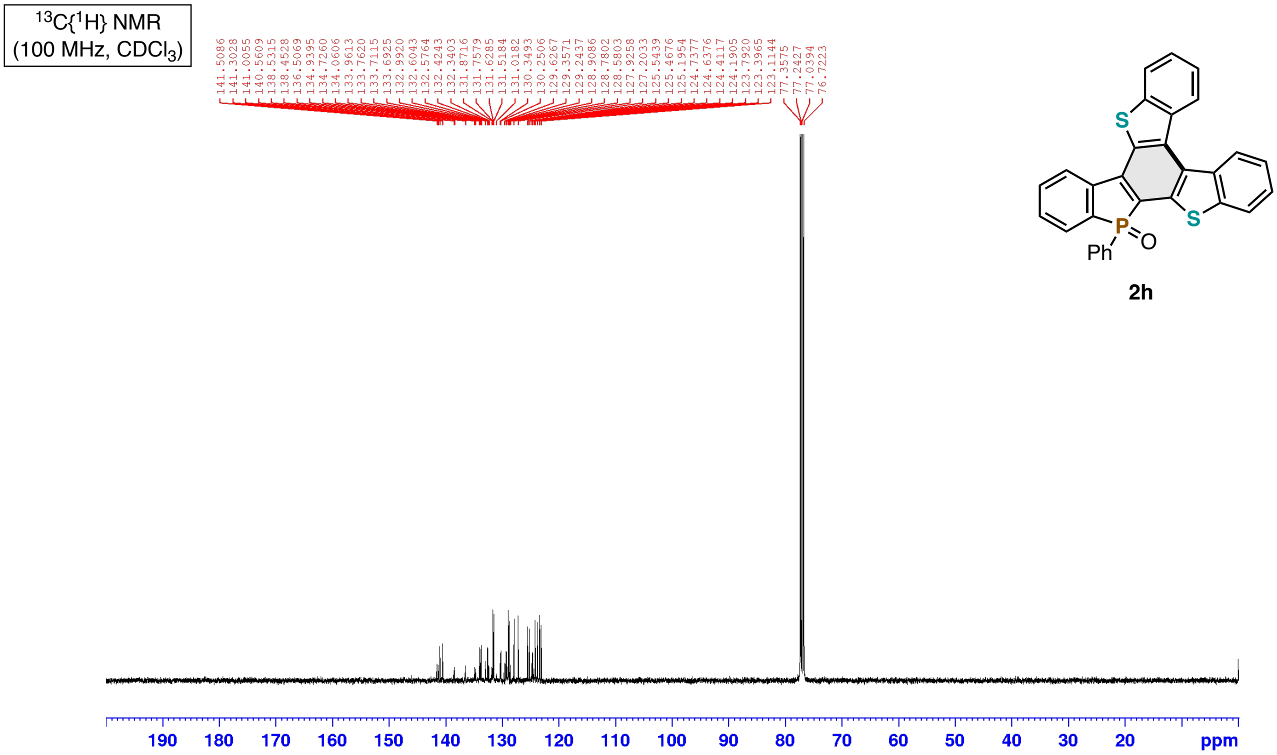

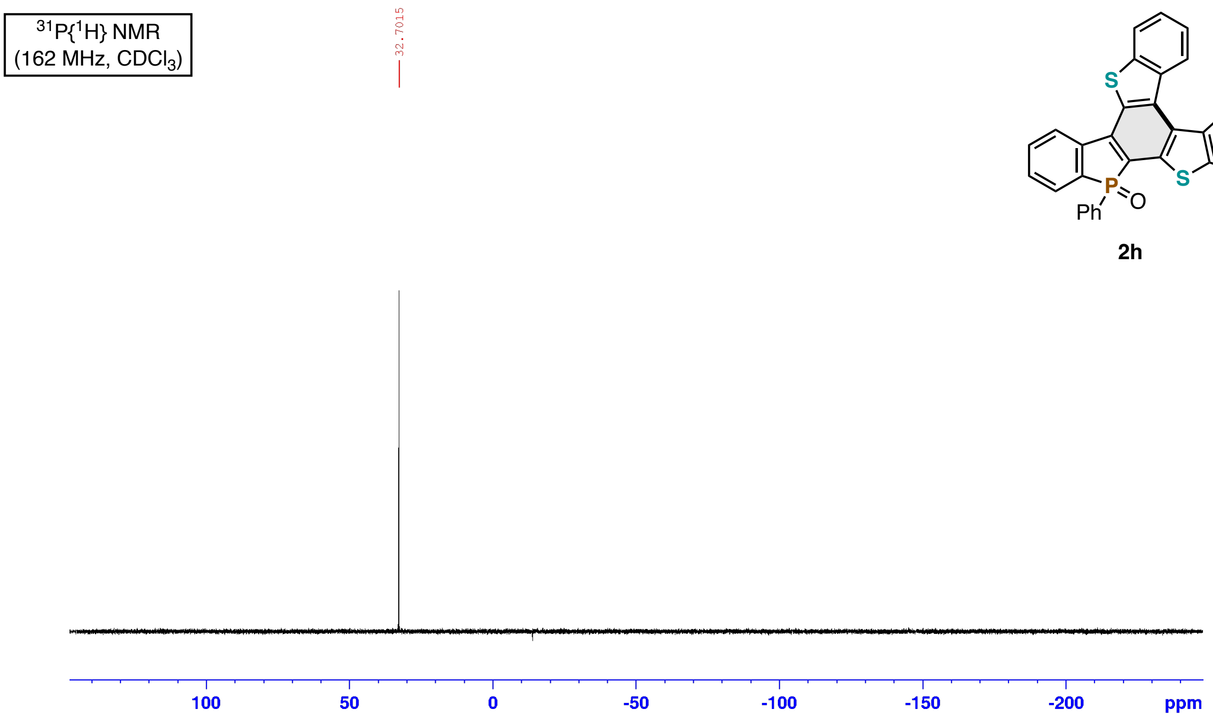

^{31}P{^{1}H} NMR
(162 MHz, CDCl$_3$)
32.7015
S
S
P
O
Ph
2h
100
50
0
-50
-100
-150
-200
ppm

[^{1}H, ^{13}C{^{1}H}, and ^{31}P{^{1}H} NMR Spectra of **2i**]

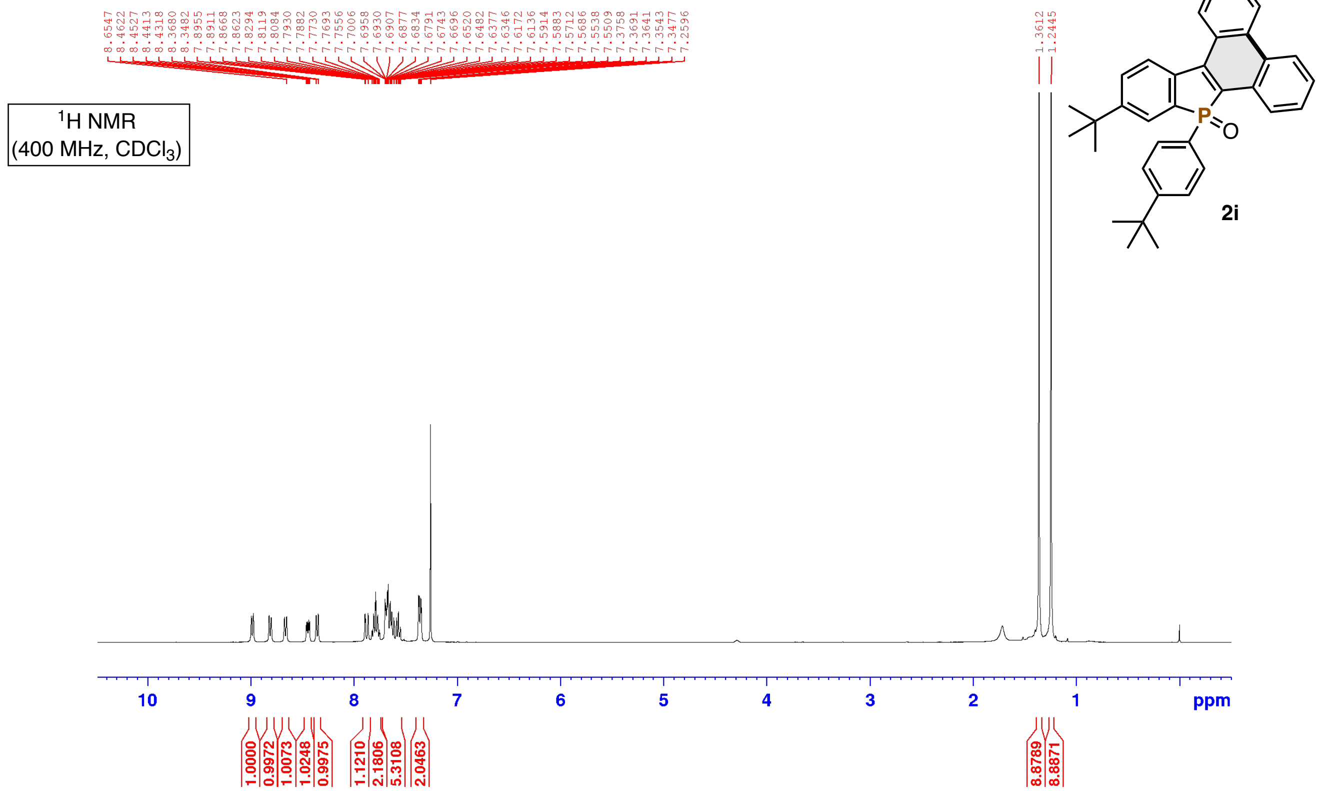

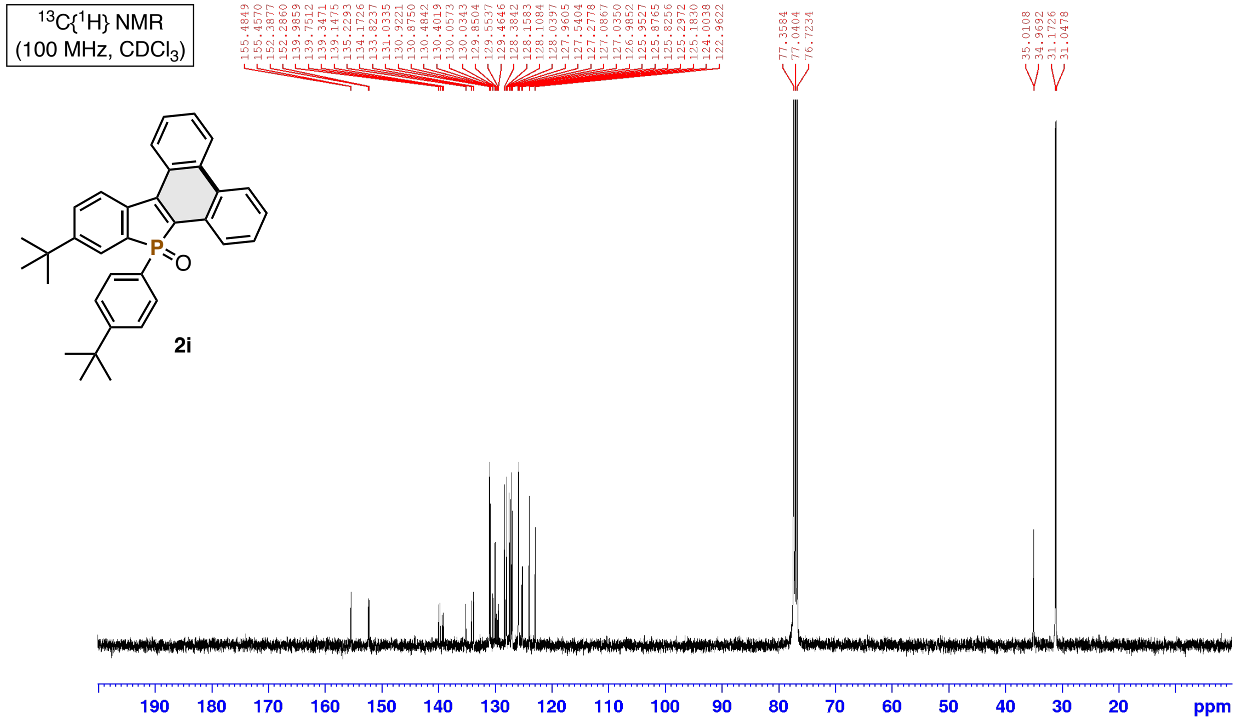

$^{31}P\{^{1}H\}$ NMR
(162 MHz, $CDCl_3$)

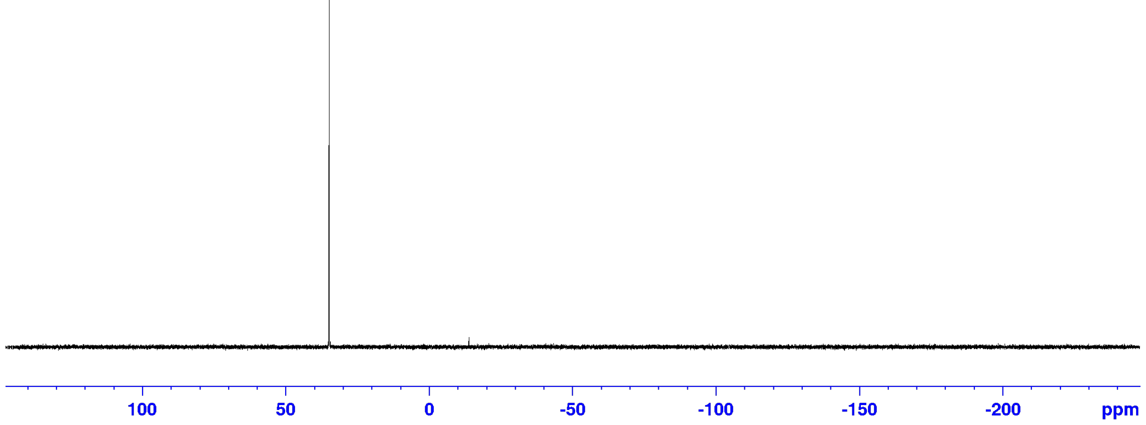

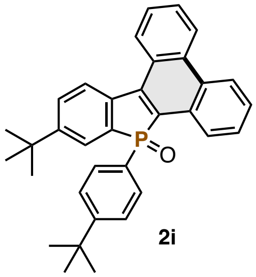

2i

[^{1}H, ^{13}C{^{1}H}, and ^{31}P{^{1}H} NMR Spectra of **2j**]

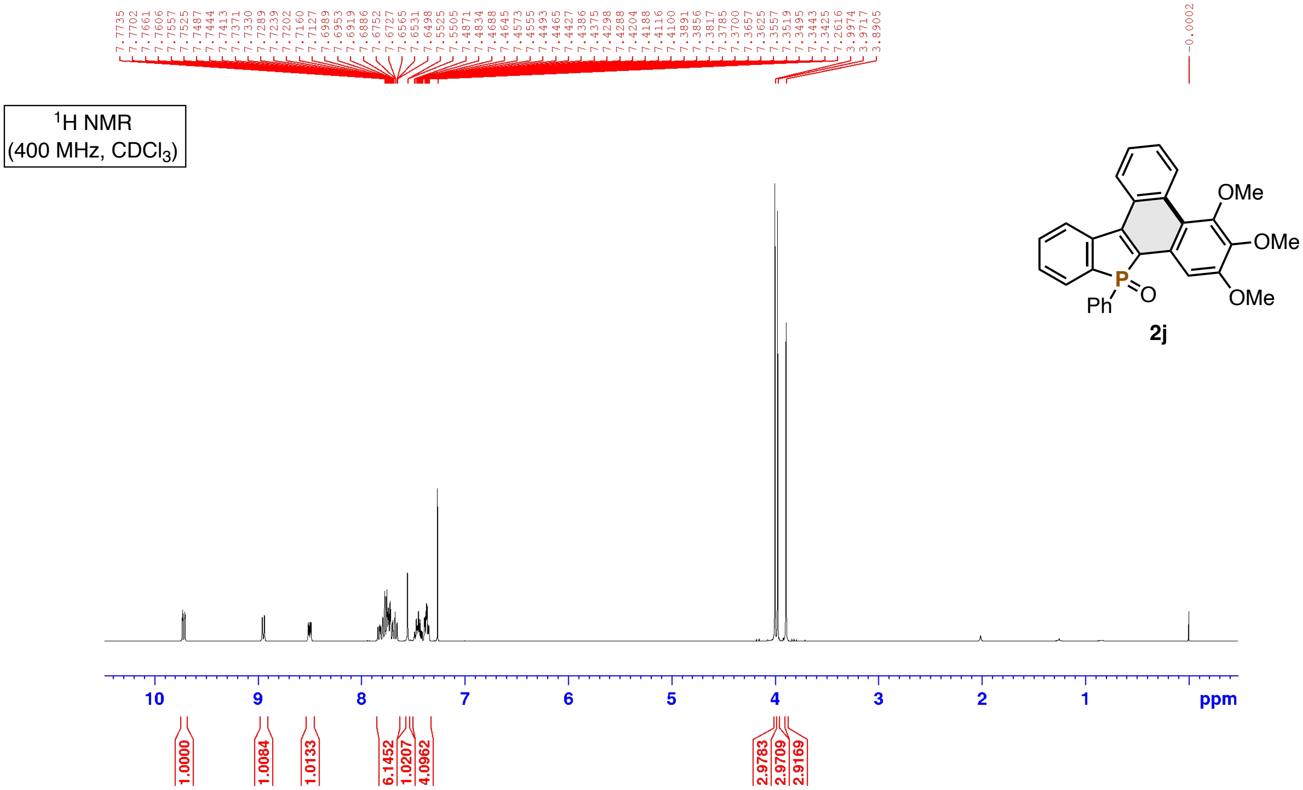

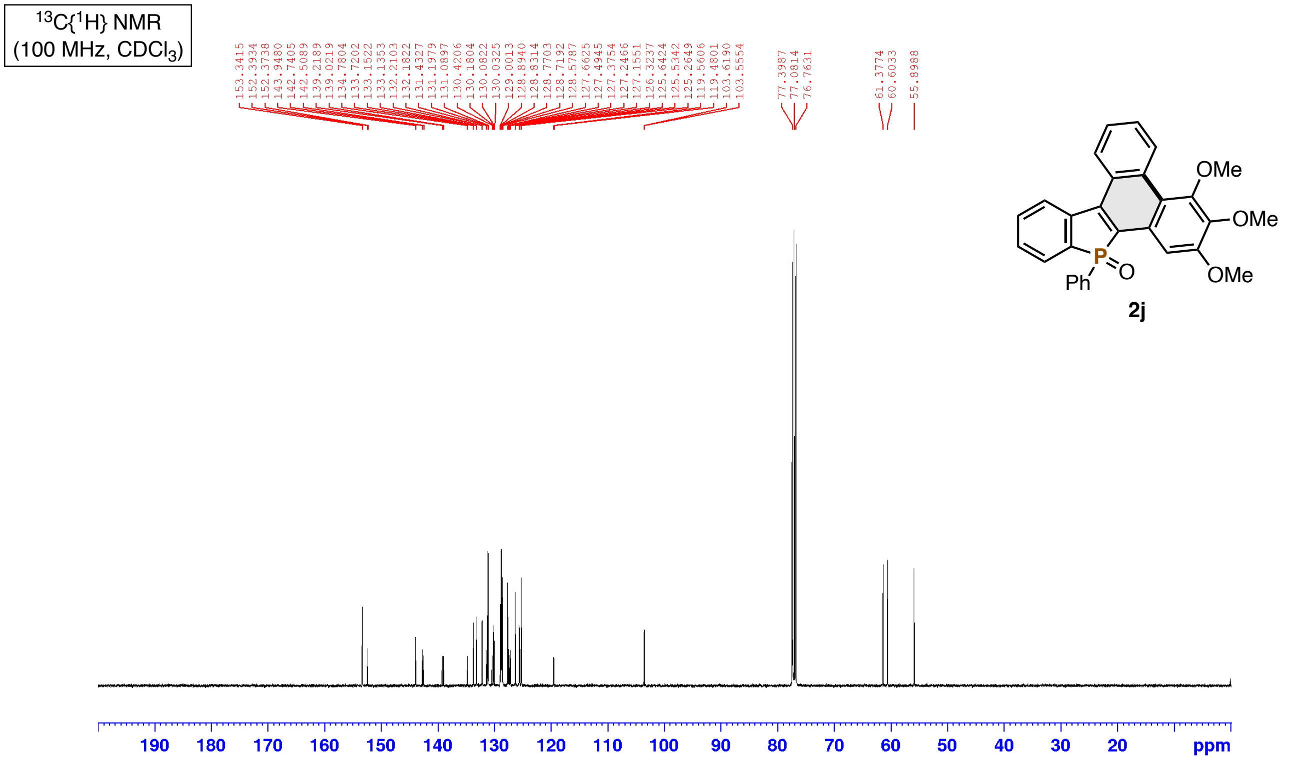

$^{31}P\{^{1}H\}$ NMR
(162 MHz, $CDCl_3$)

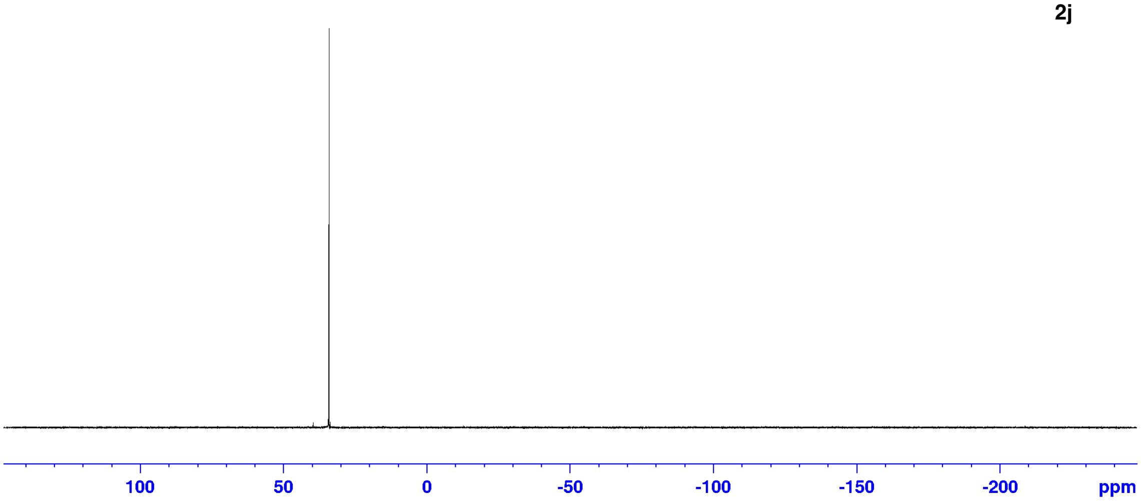

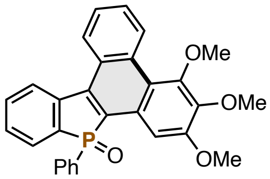

2j

[^{1}H, $^{13}C\{^{1}H\}$, and $^{31}P\{^{1}H\}$ NMR Spectra of **2k**]

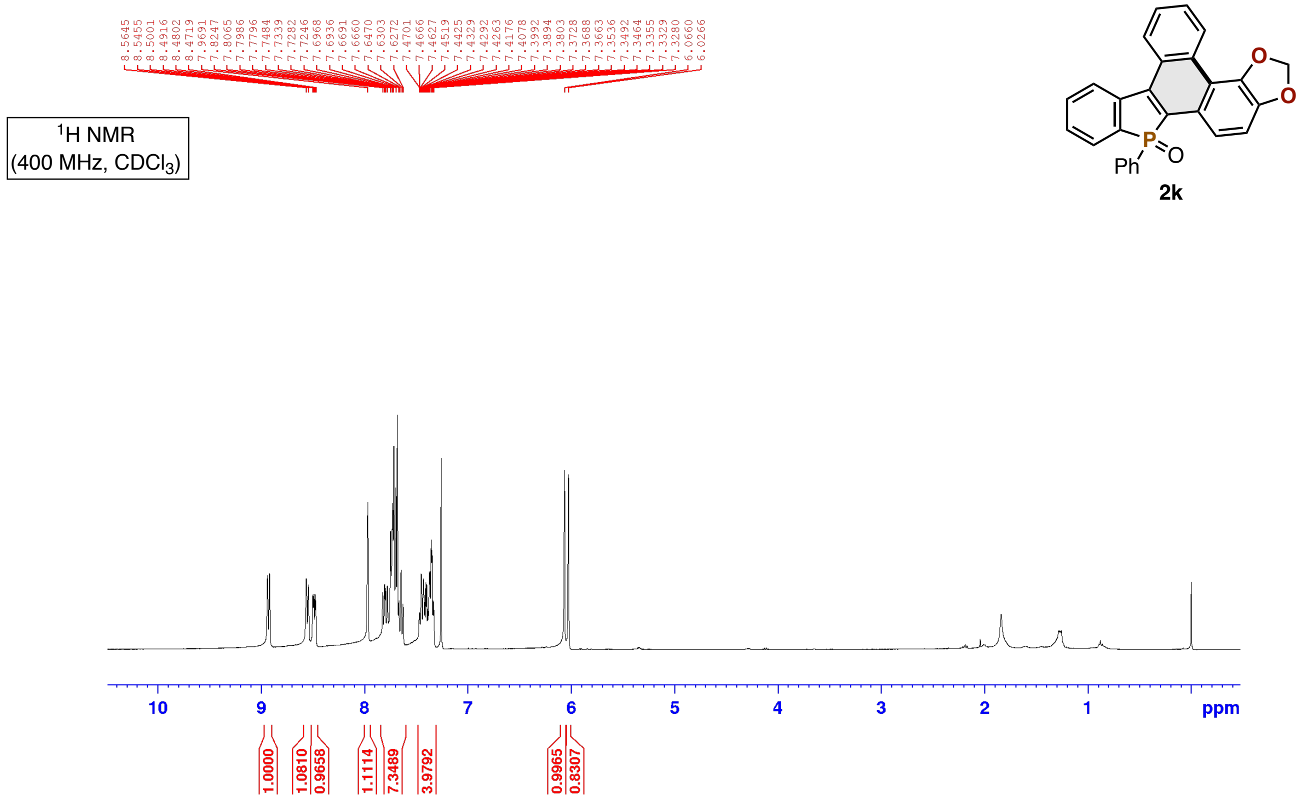

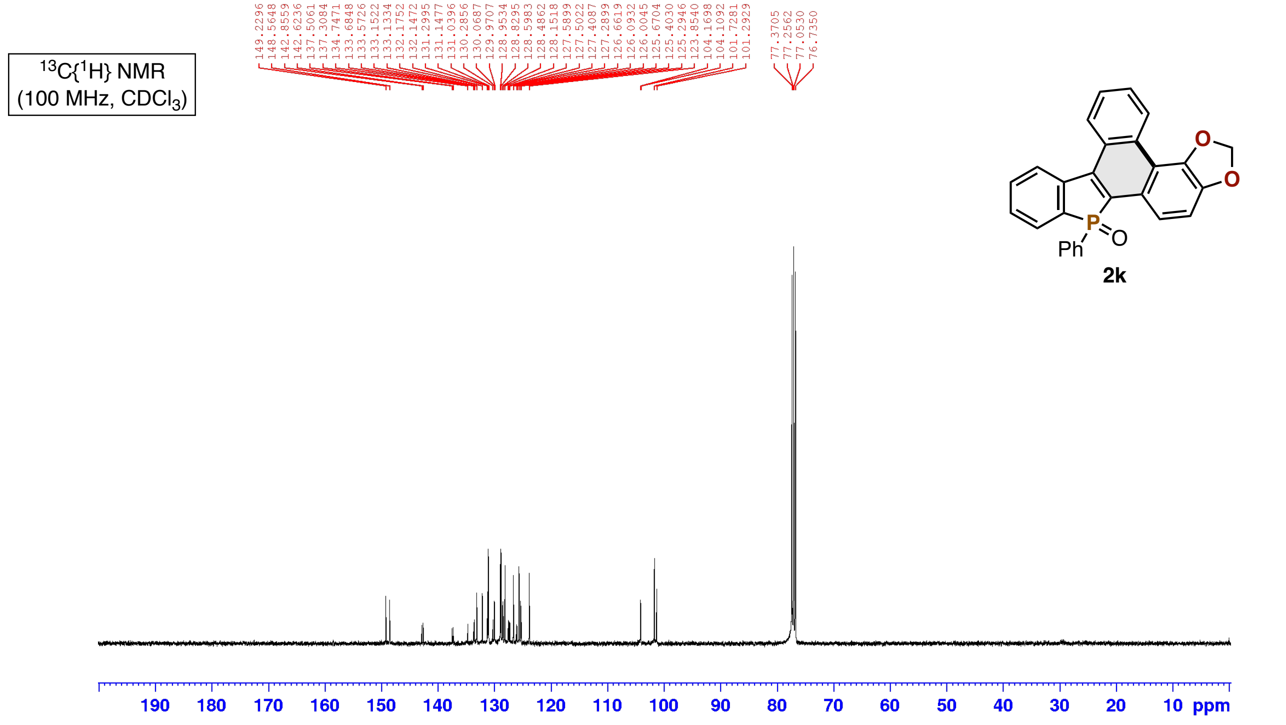

$^{31}P\{^1H\}$ NMR
(162 MHz, $CDCl_3$)

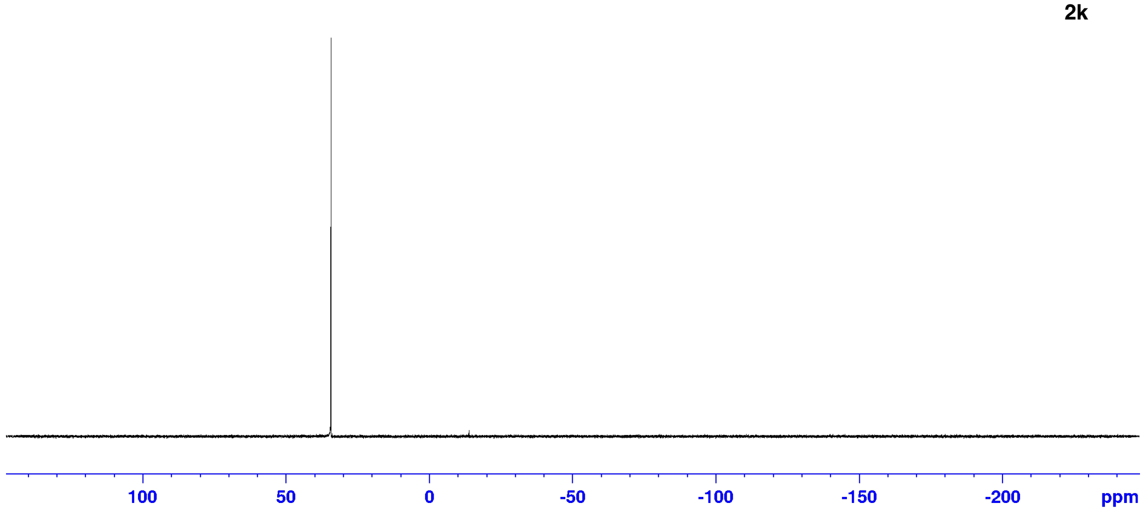

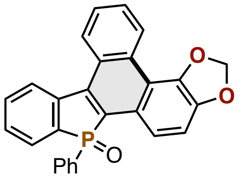

2k

[^{1}H, ^{13}C{^{1}H}, and ^{31}P{^{1}H} NMR Spectra of **2l**]

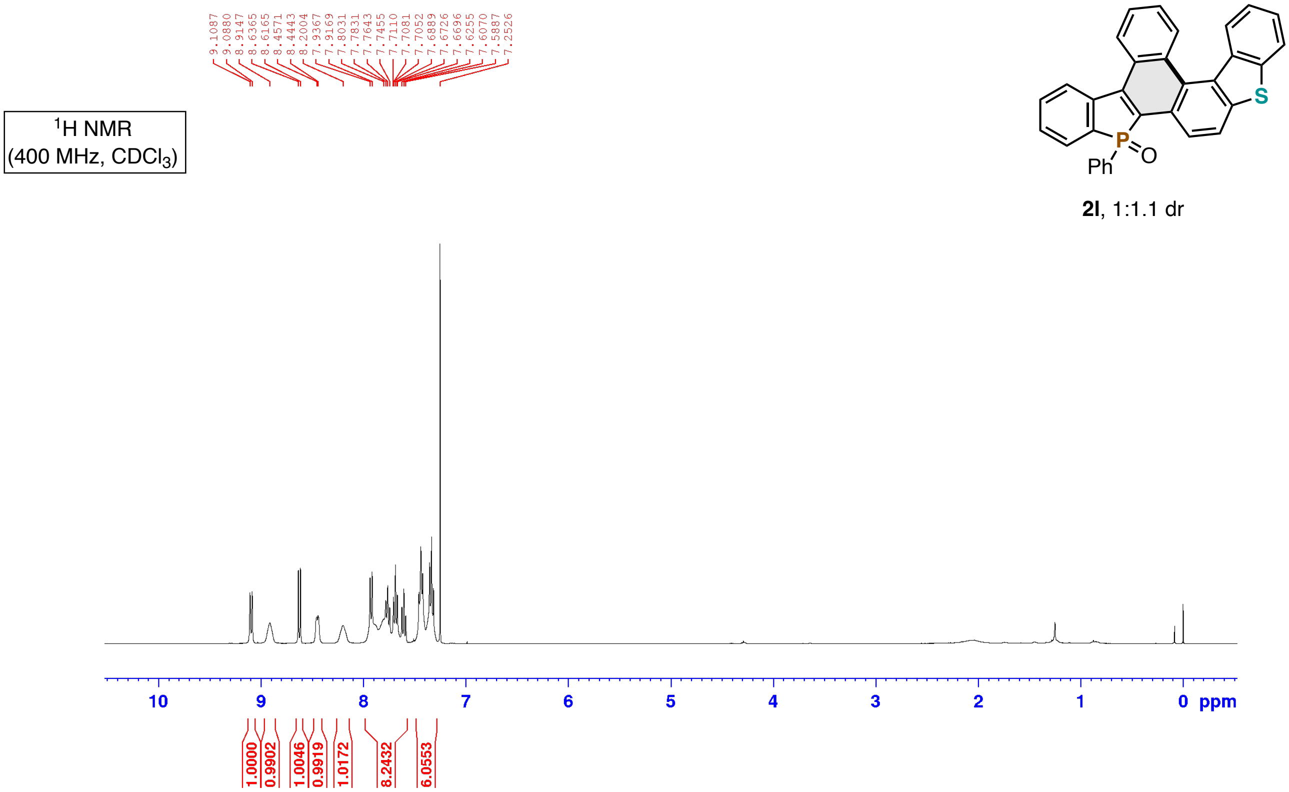

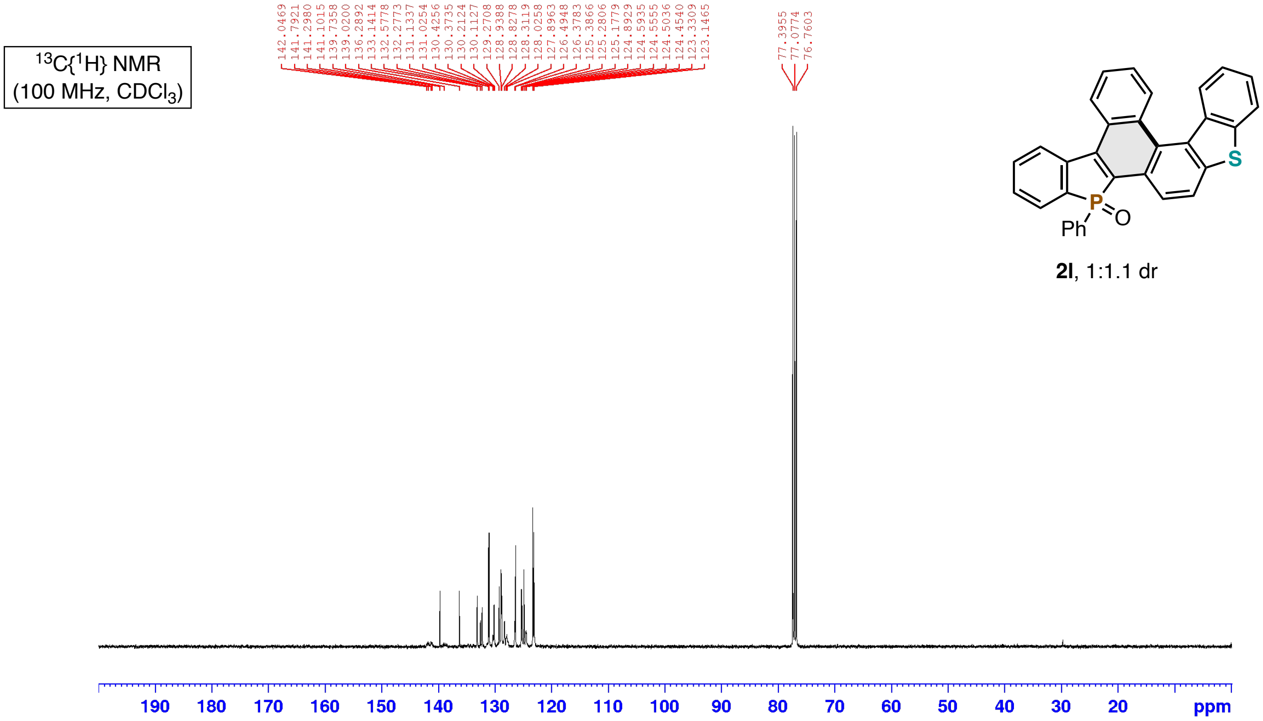

2l, 1:1.1 dr

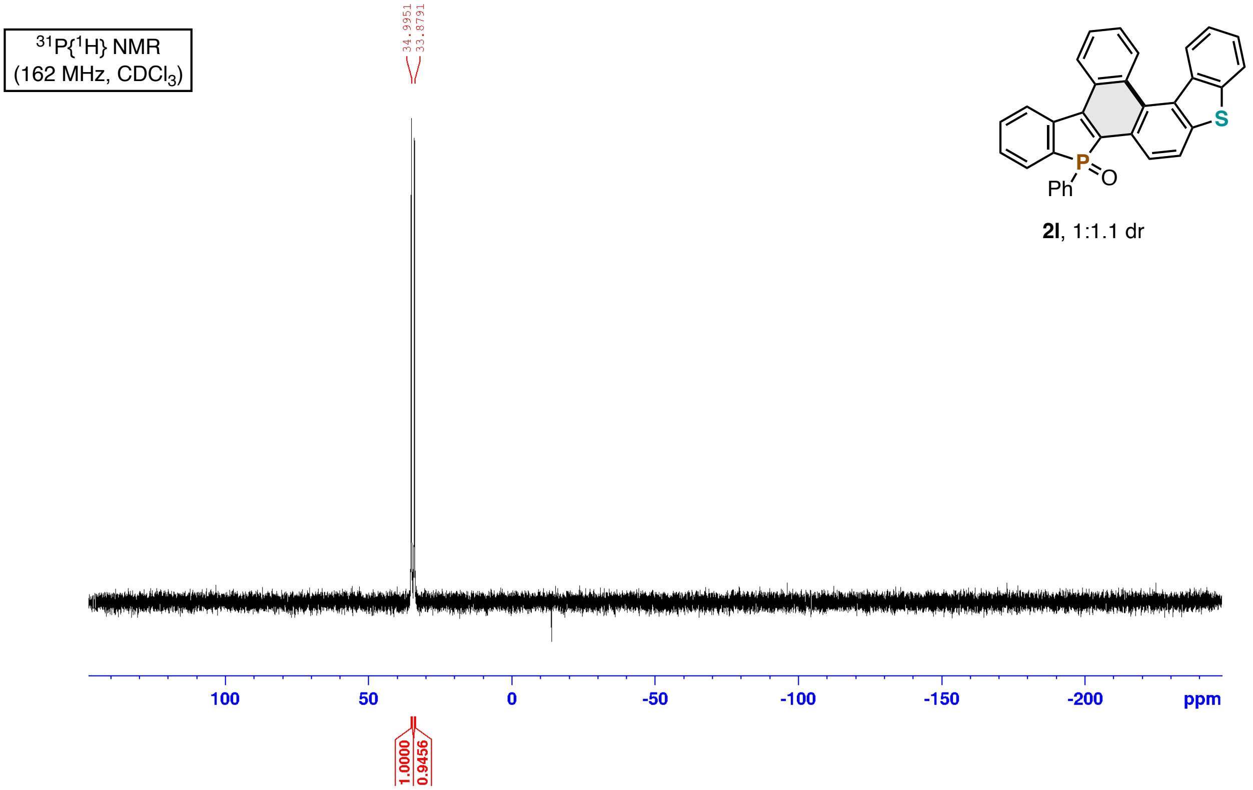

2l, 1:1.1 dr

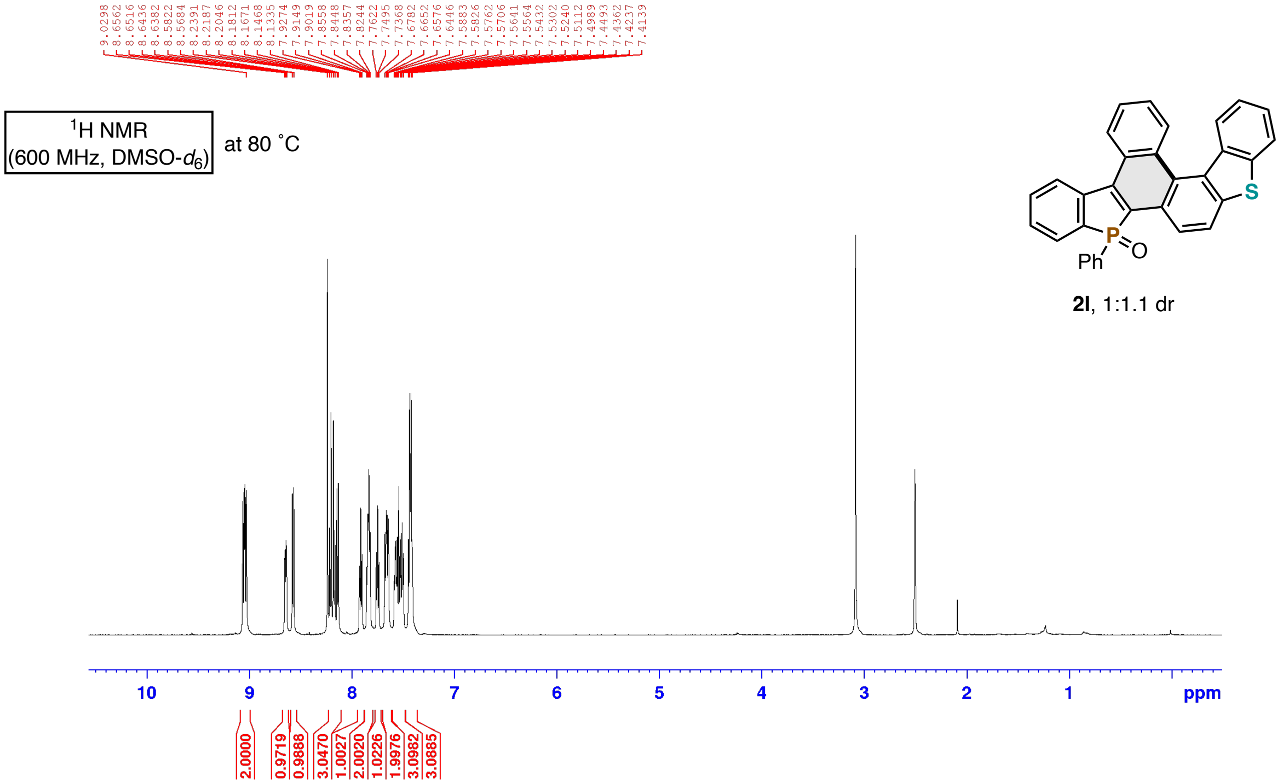

2l, 1:1.1 dr

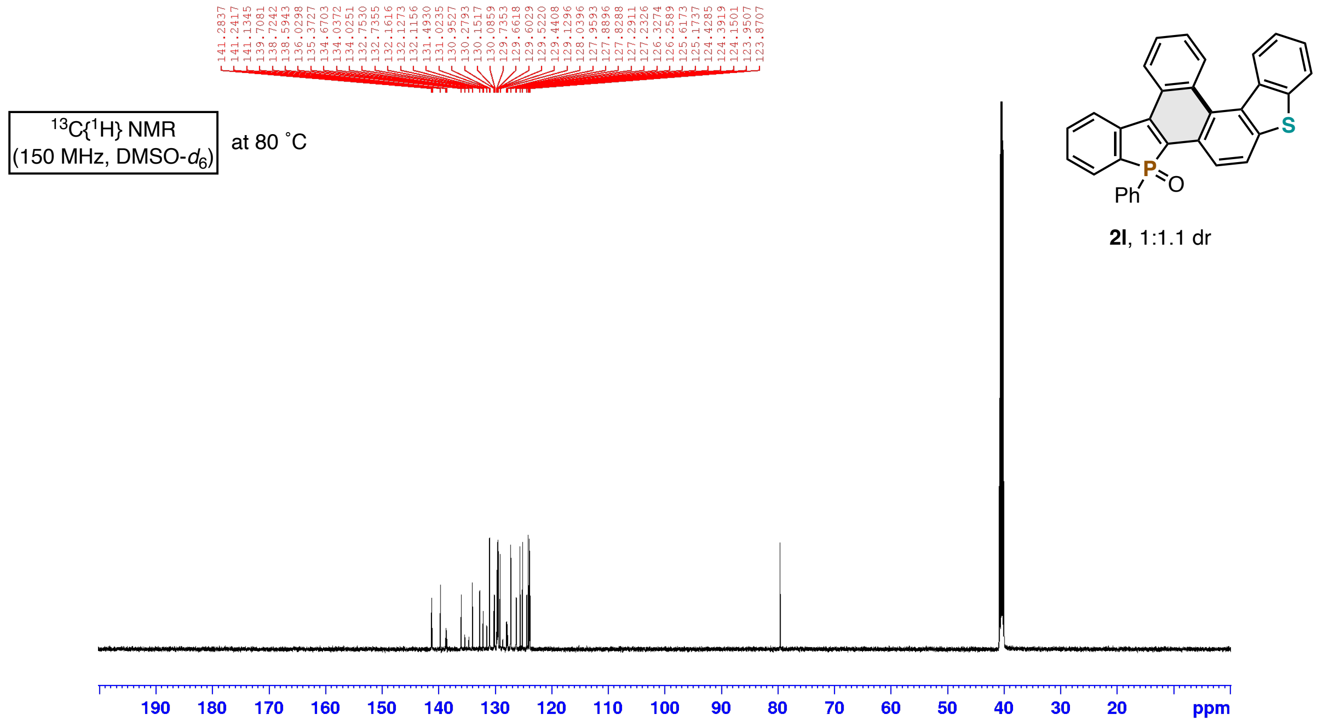

2l, 1:1.1 dr

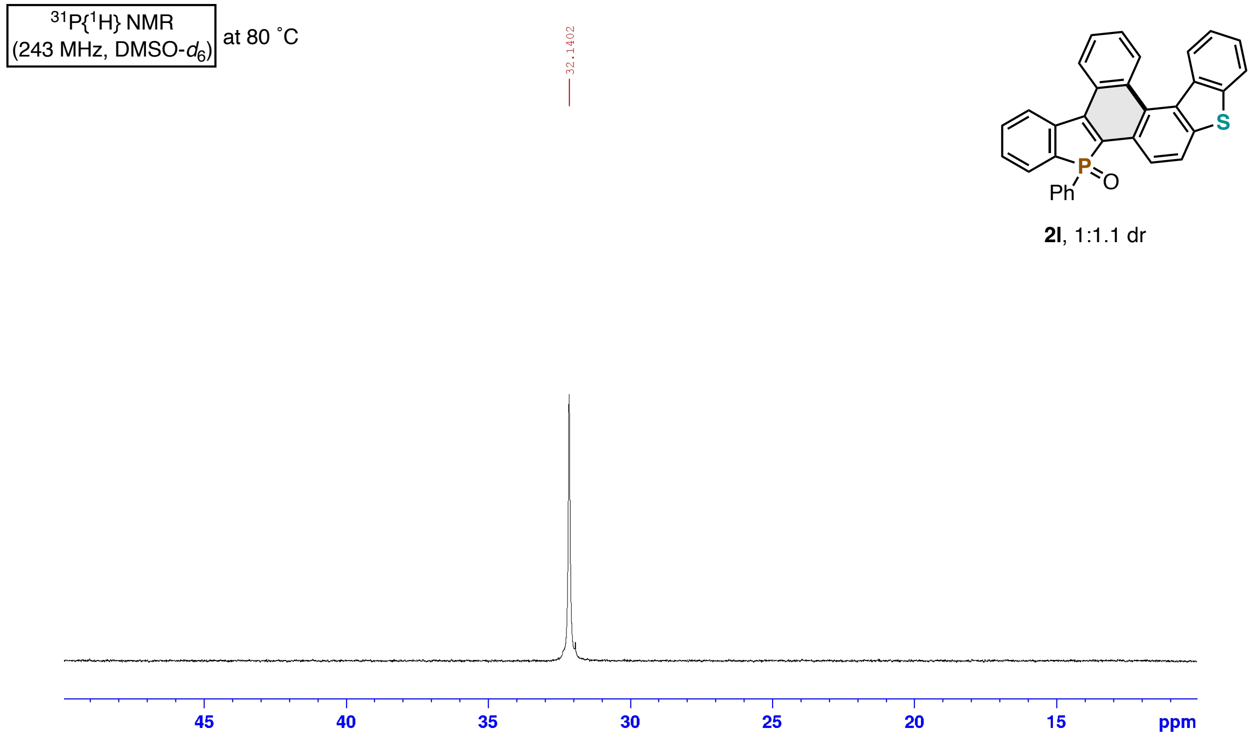

2l, 1:1.1 dr

[^{1}H, ^{13}C{^{1}H}, and ^{31}P{^{1}H} NMR Spectra of **2l'**]

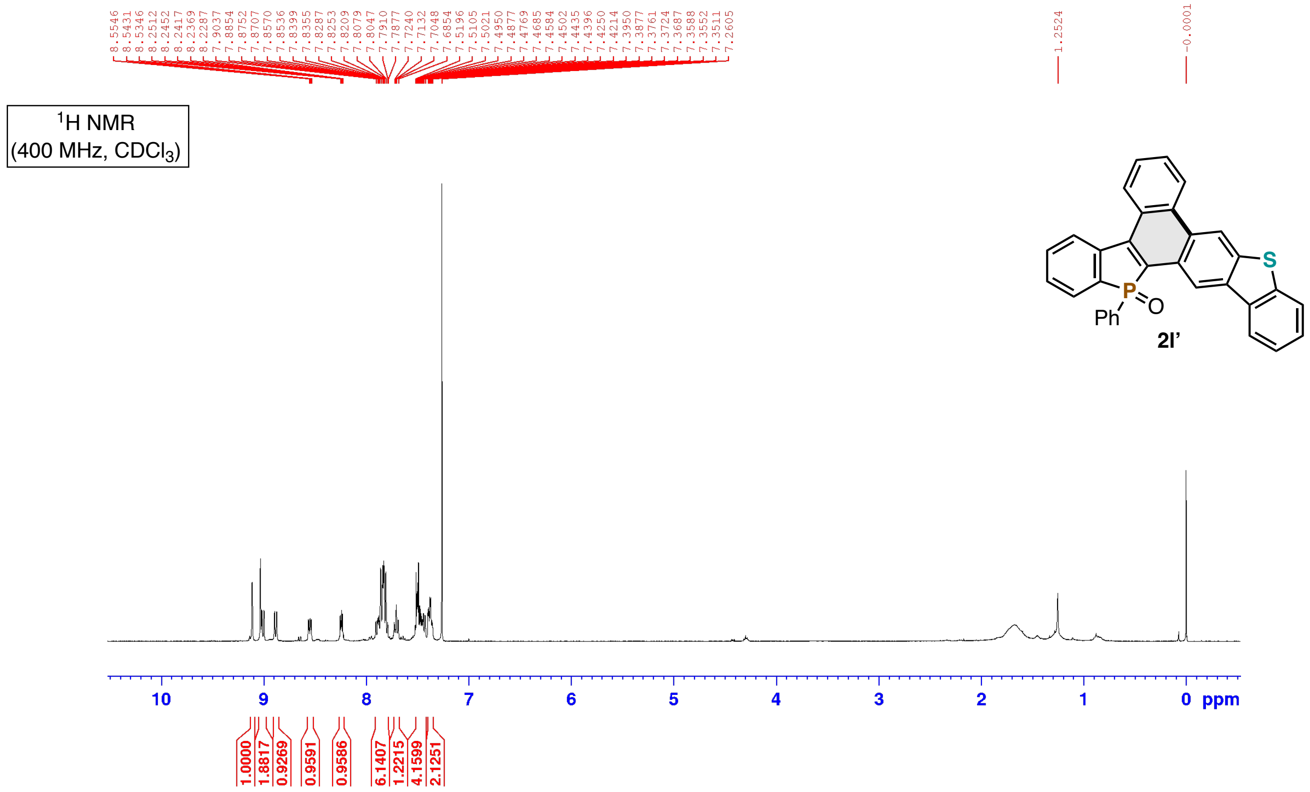

2l'

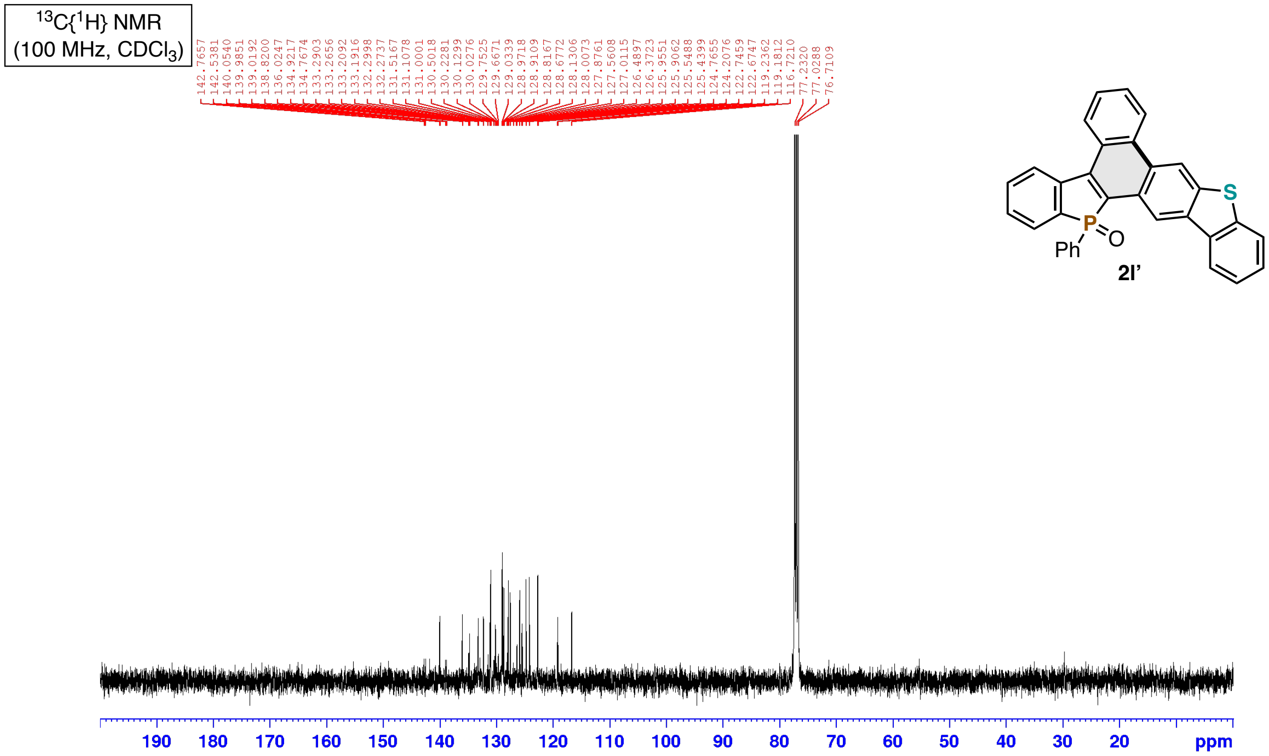

2l'

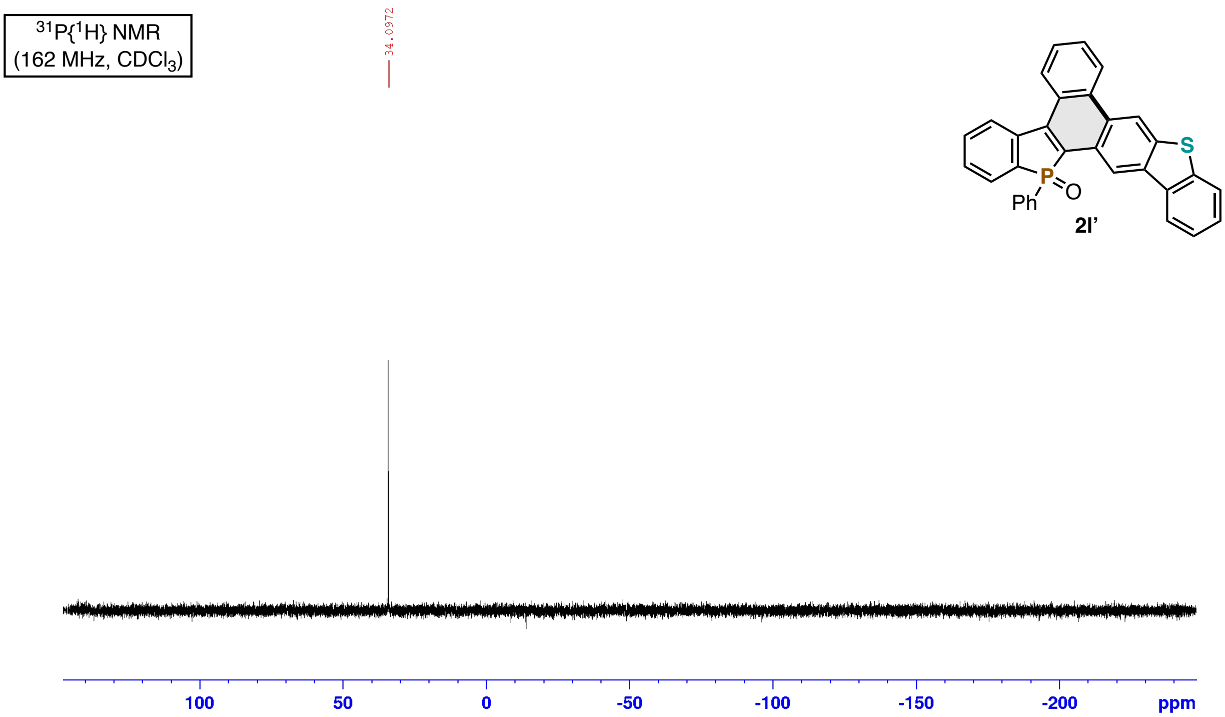
$^{31}P\{^{1}H\}$ NMR
(162 MHz, $CDCl_3$)
34.0972
S
P
O
Ph
2l'
100
50
0
-50
-100
-150
-200
ppm

[^{1}H, ^{13}C{^{1}H}, and ^{31}P{^{1}H} NMR Spectra of **2m**]

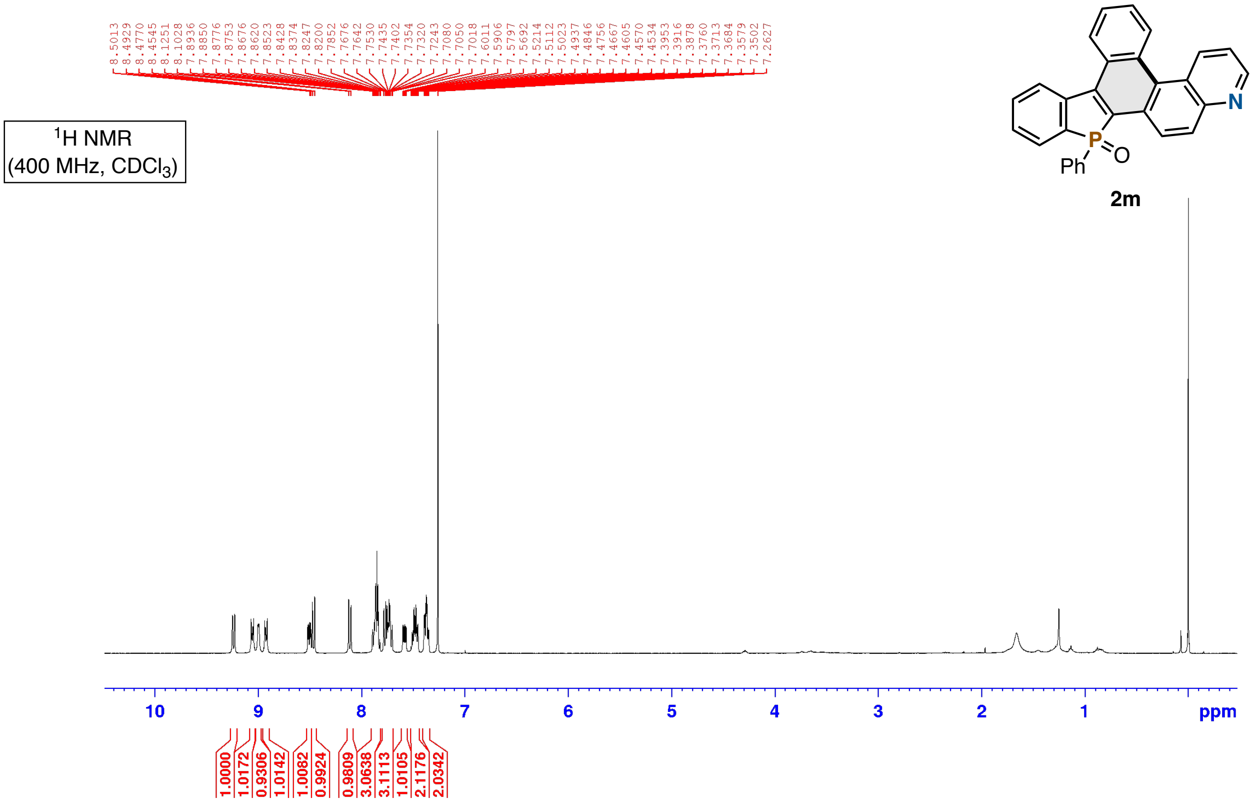

2m

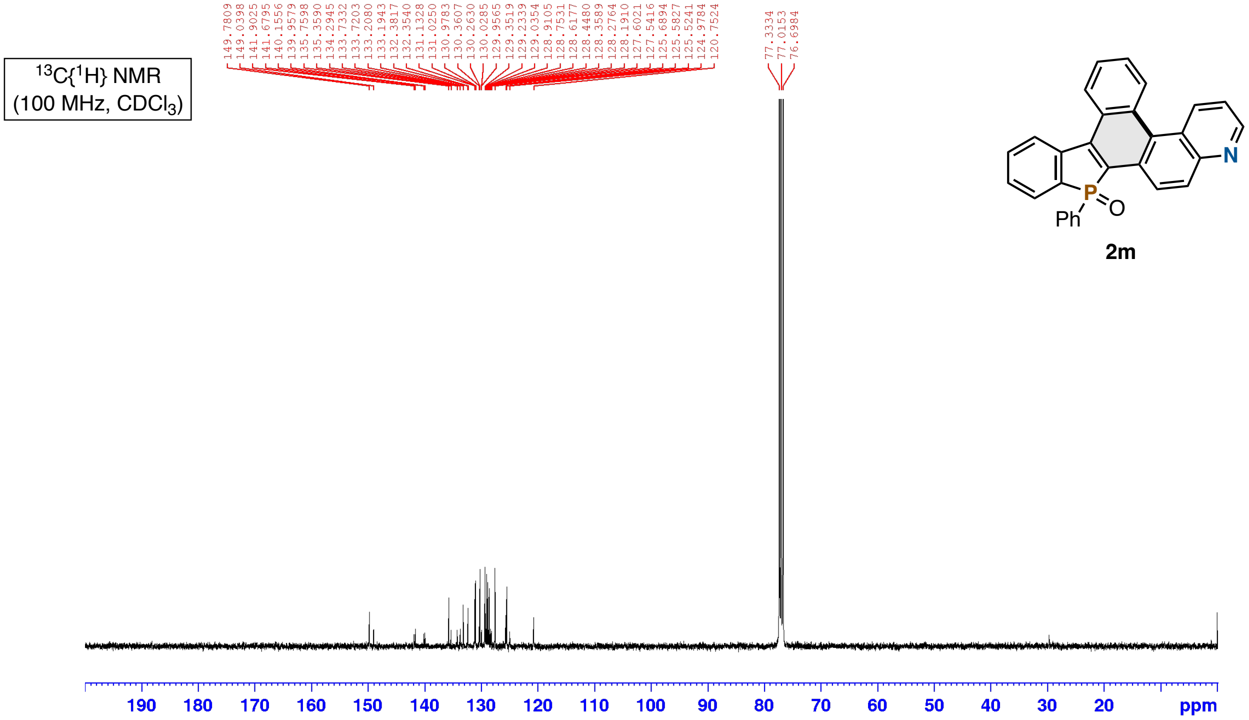

2m

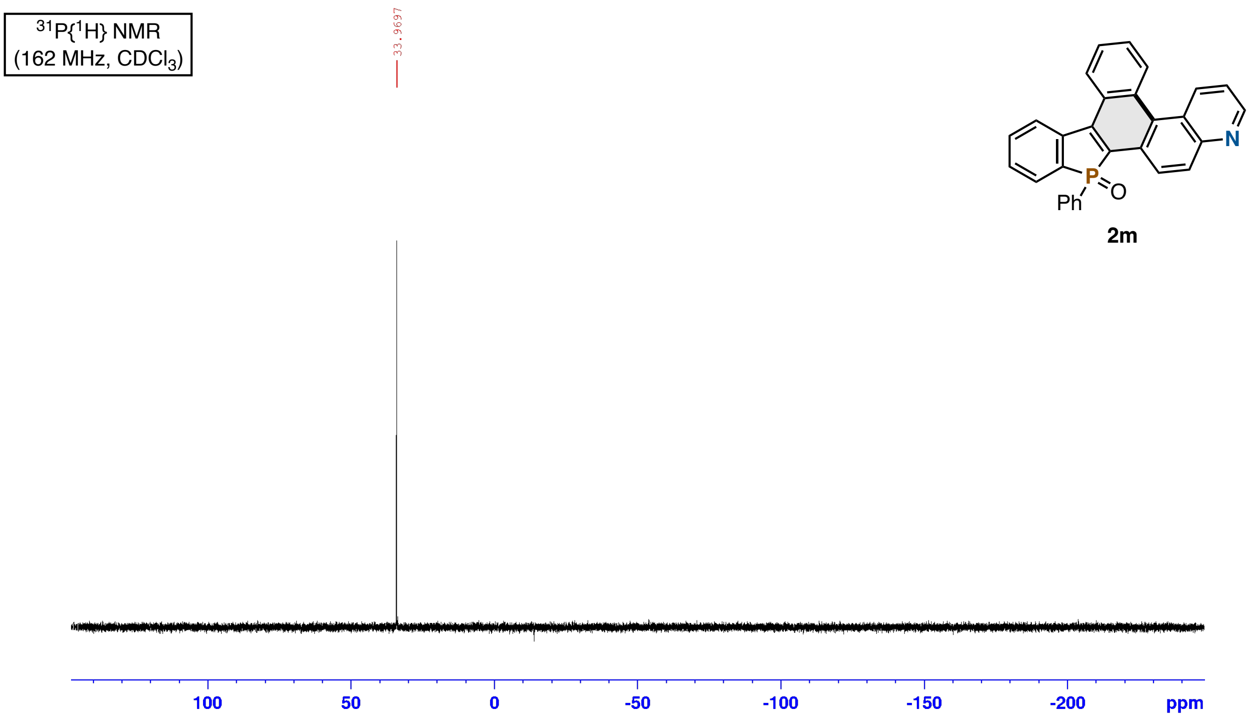

31P{1H} NMR
(162 MHz, CDCl3)
33.9697
Ph
P
O
N
2m
100
50
0
-50
-100
-150
-200
ppm

[^{1}H, ^{13}C{^{1}H}, and ^{31}P{^{1}H} NMR Spectra of **2n**]

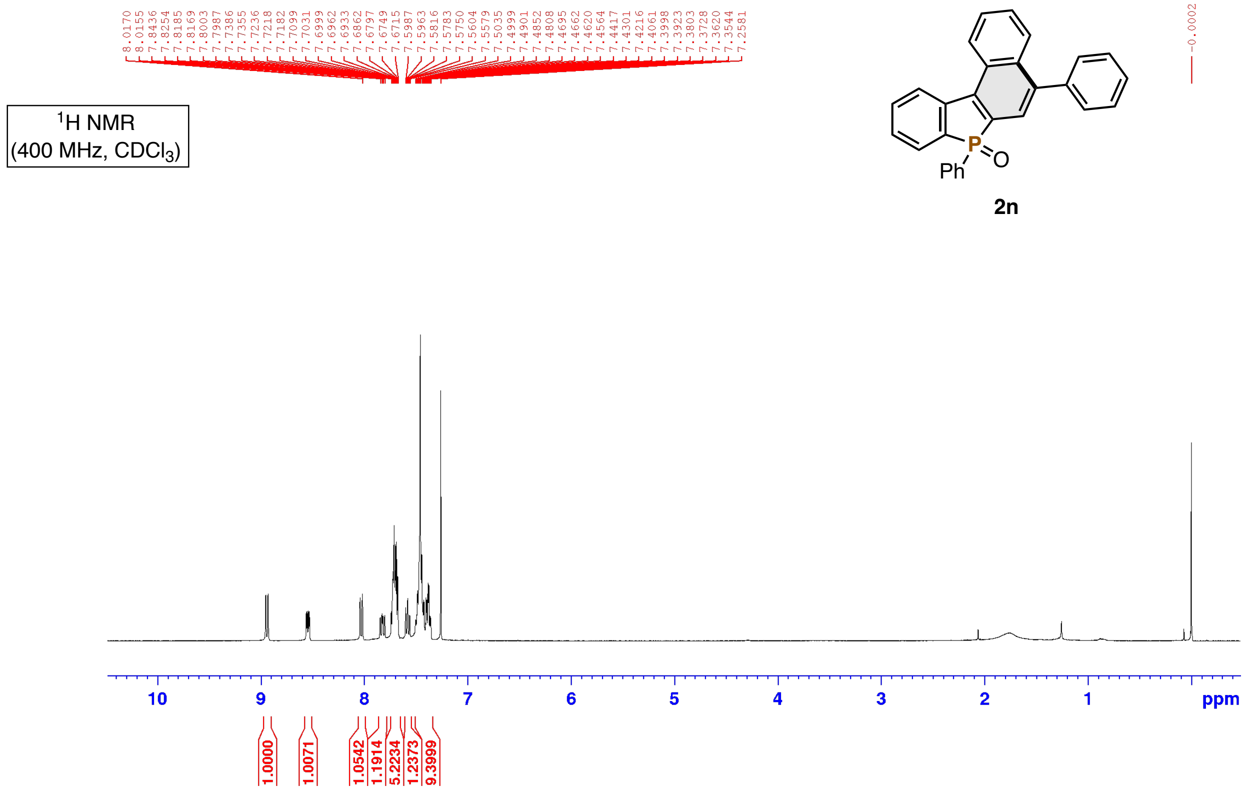

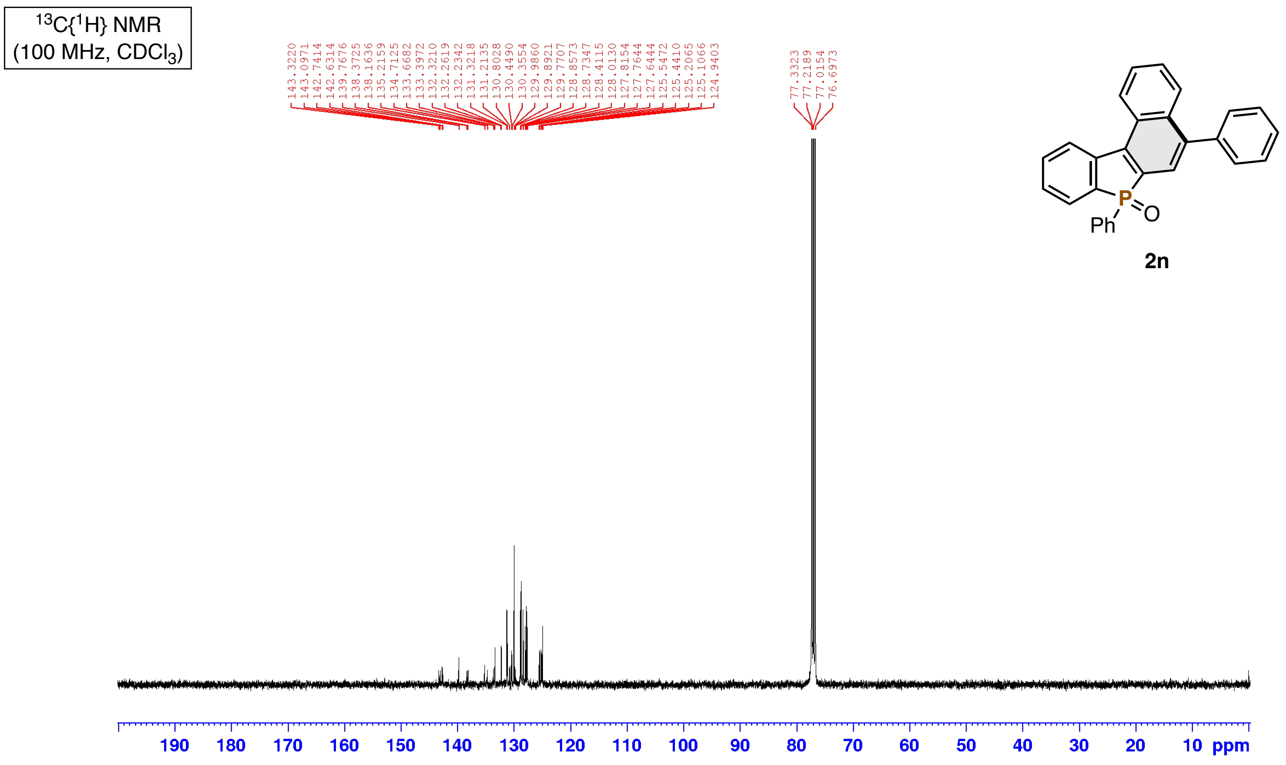

$^{31}P\{^1H\}$ NMR
(162 MHz, $CDCl_3$)

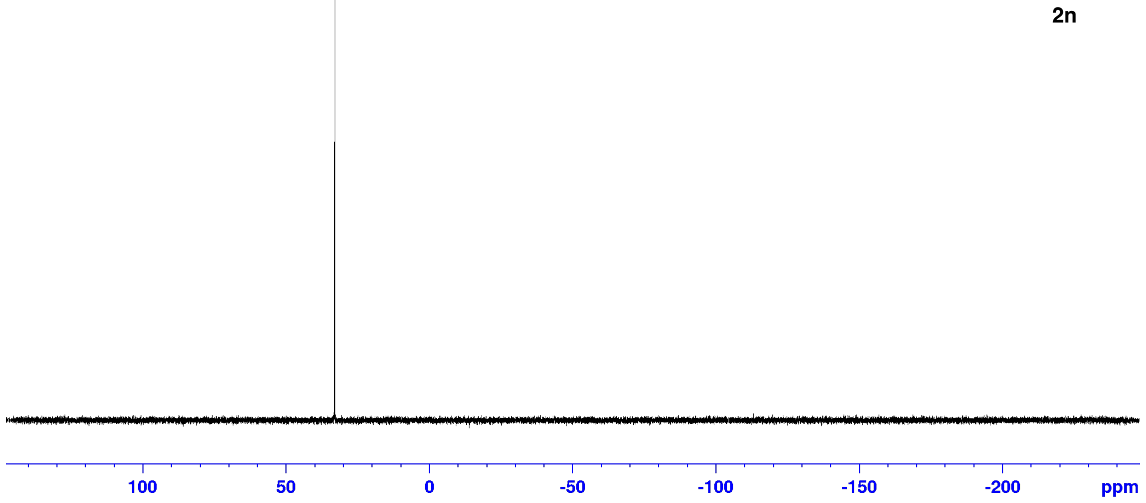

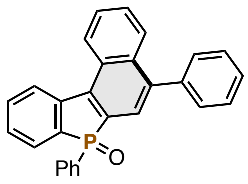

2n

[^{1}H, $^{13}C\{^{1}H\}$, and $^{31}P\{^{1}H\}$ NMR Spectra of **2o**]

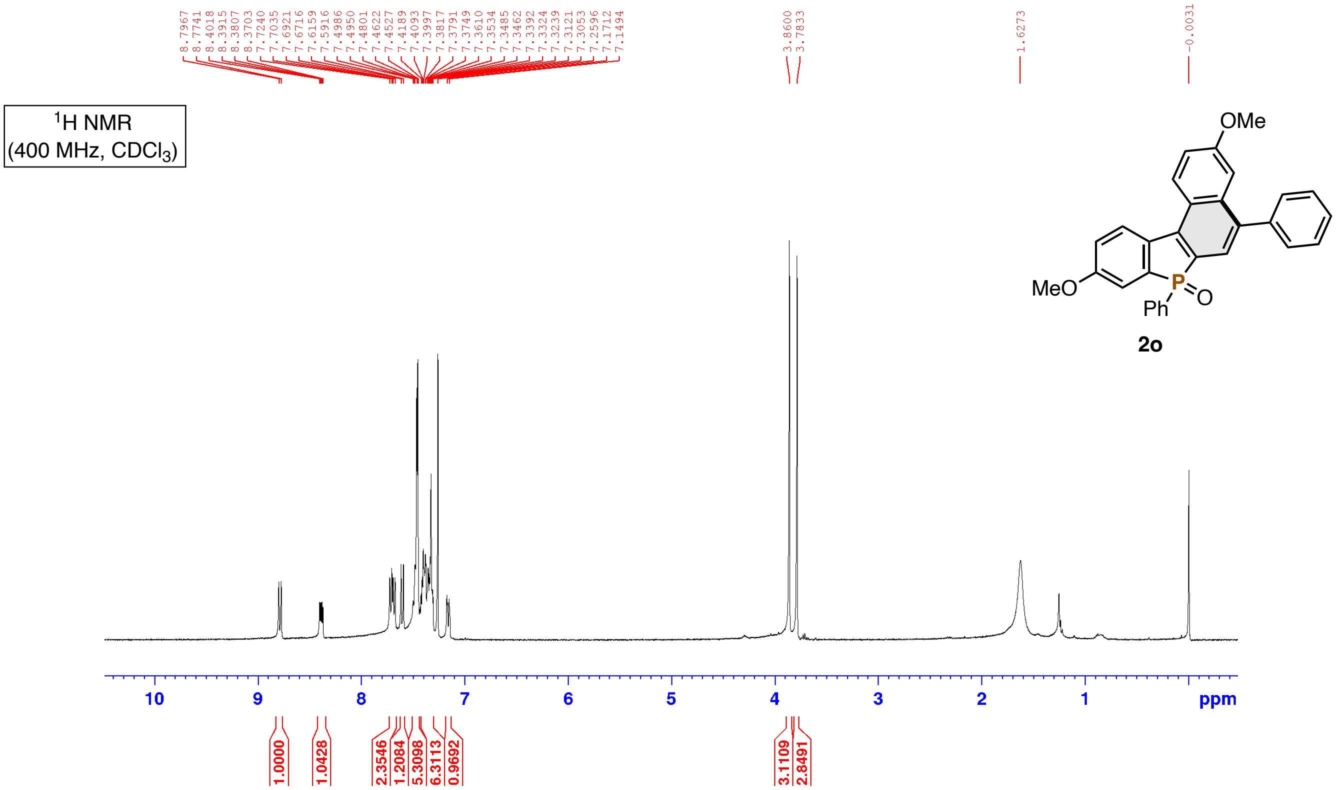

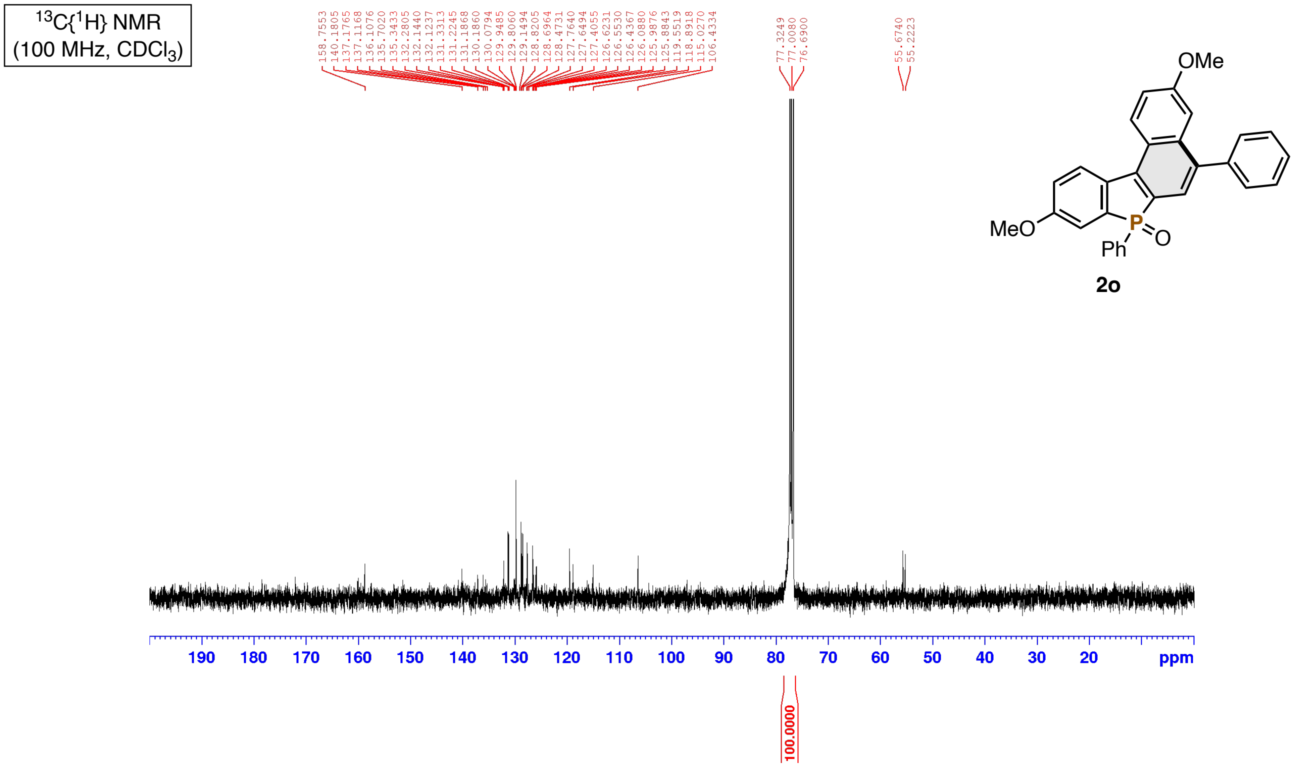

$^{31}P\{^{1}H\}$ NMR
(162 MHz, $CDCl_3$)

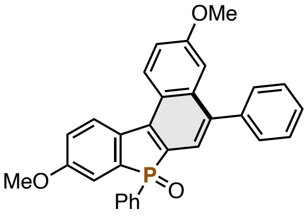

2o

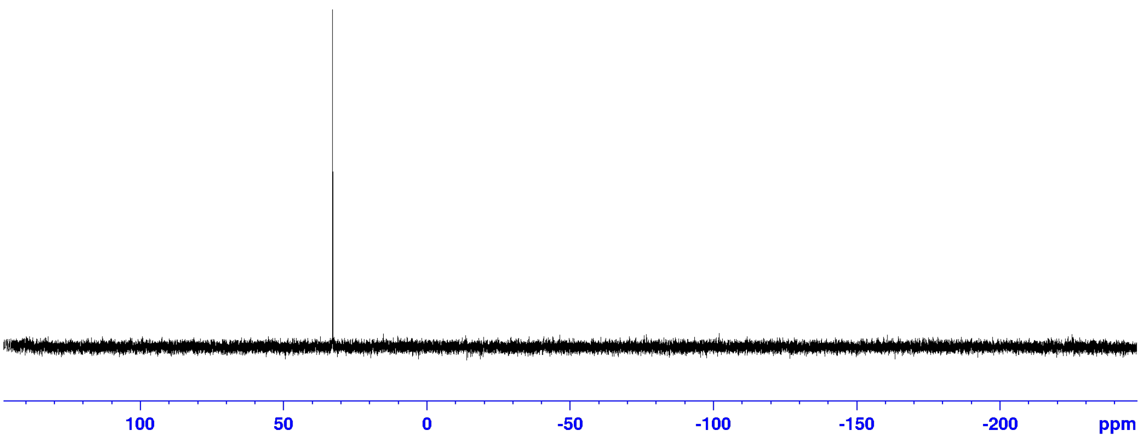

[^{1}H, ^{13}C{^{1}H}, and ^{31}P{^{1}H} NMR Spectra of **2o-H**]

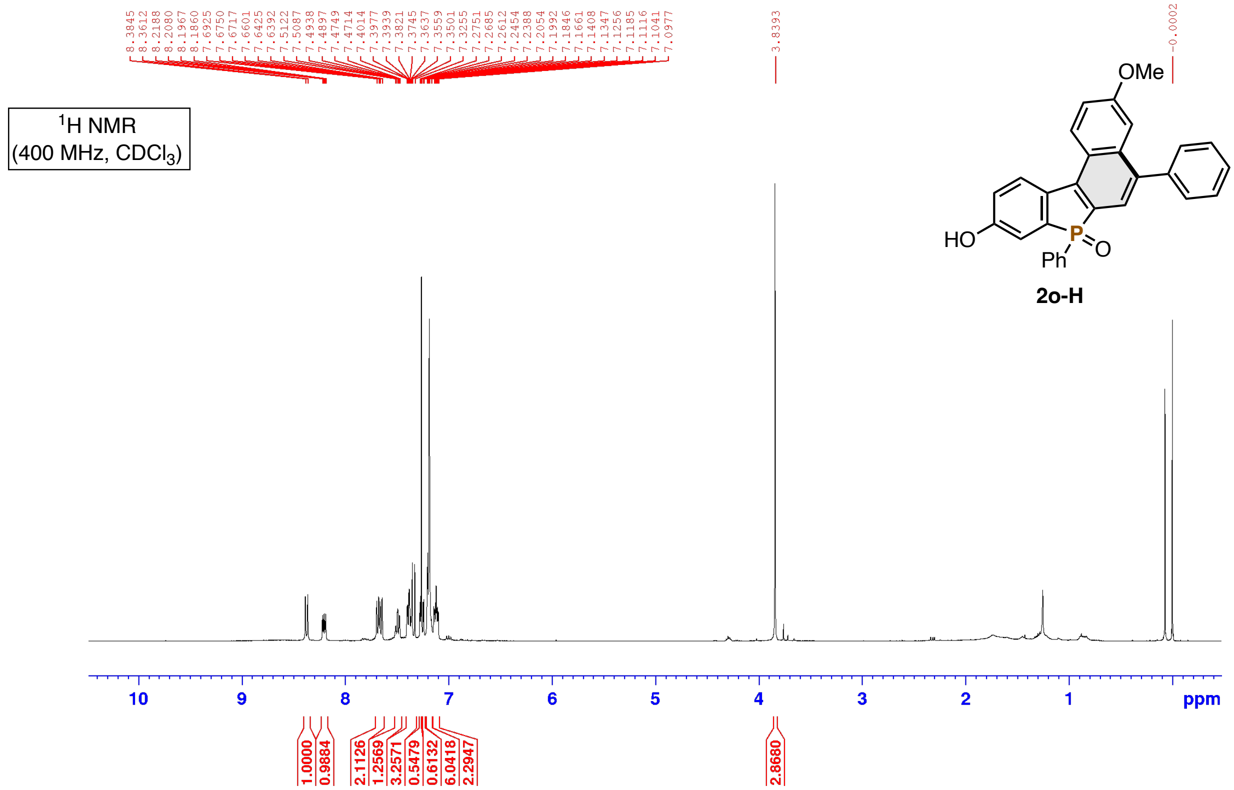

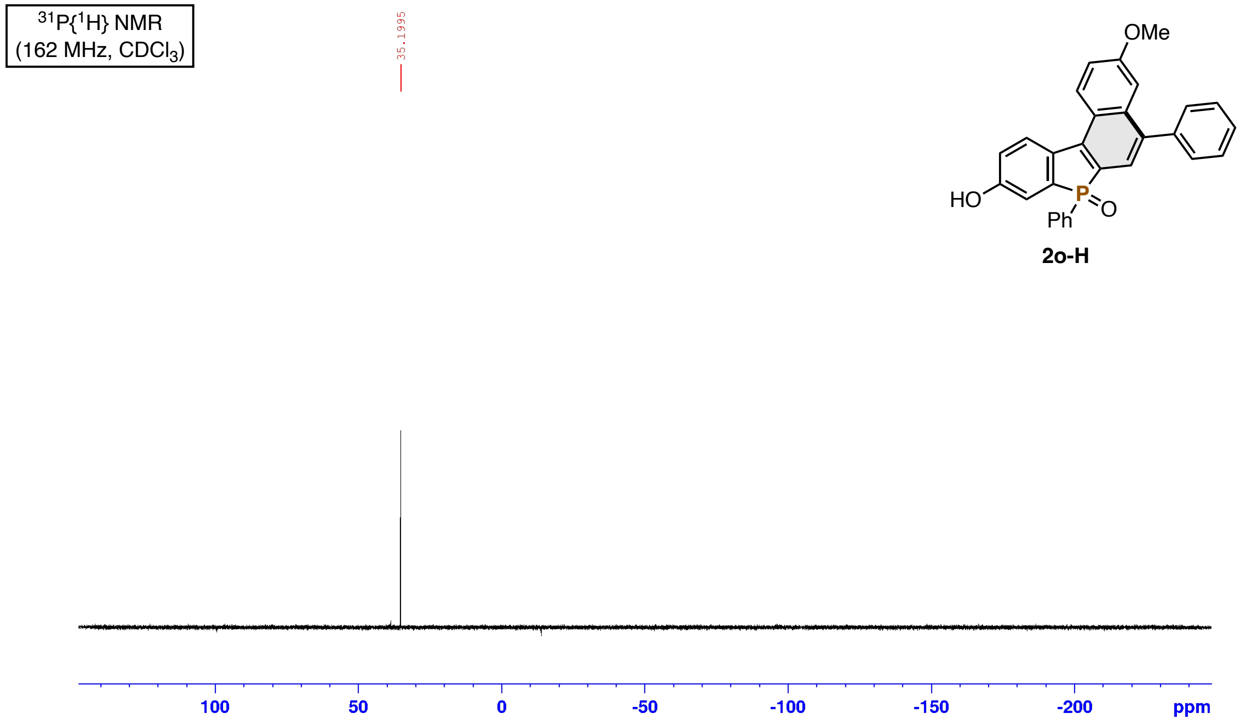

[^{1}H, ^{13}C{^{1}H}, and ^{31}P{^{1}H} NMR Spectra of **3fa**]

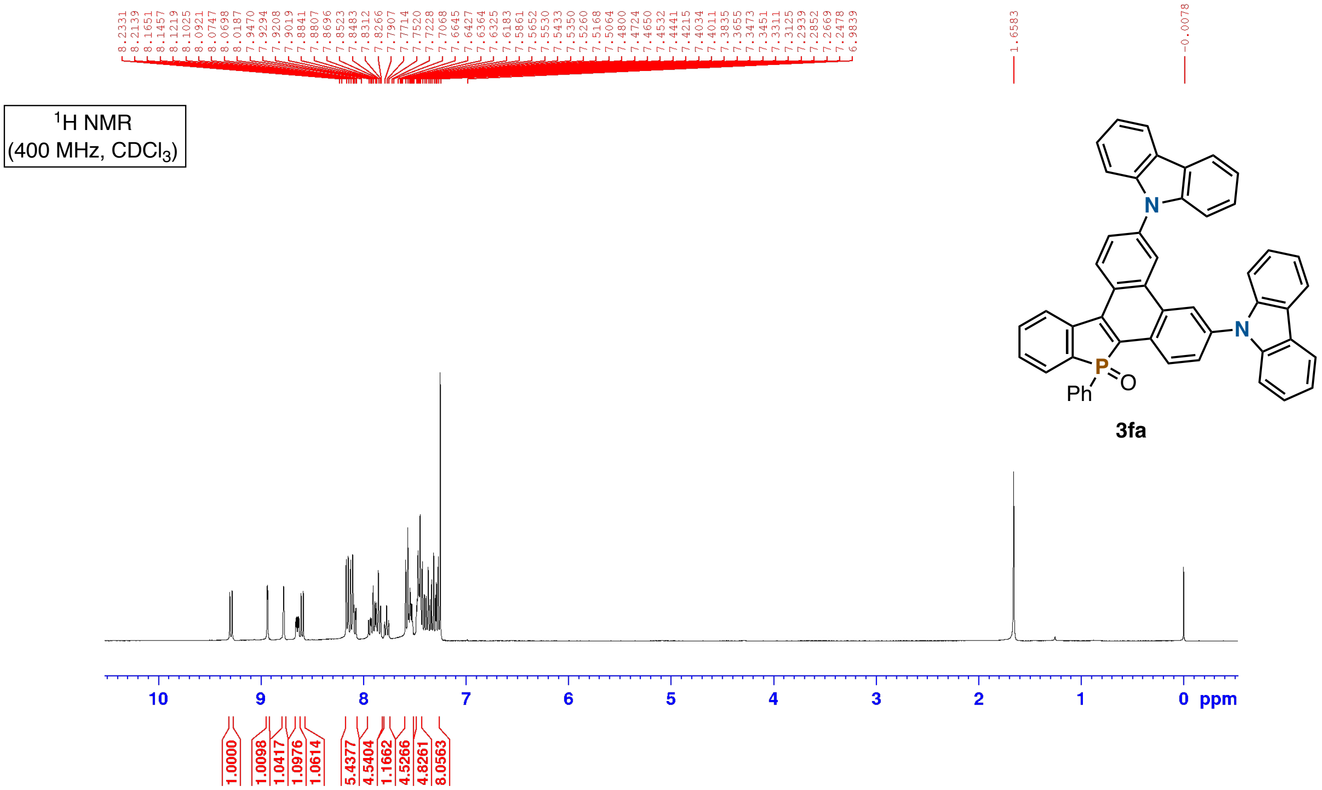

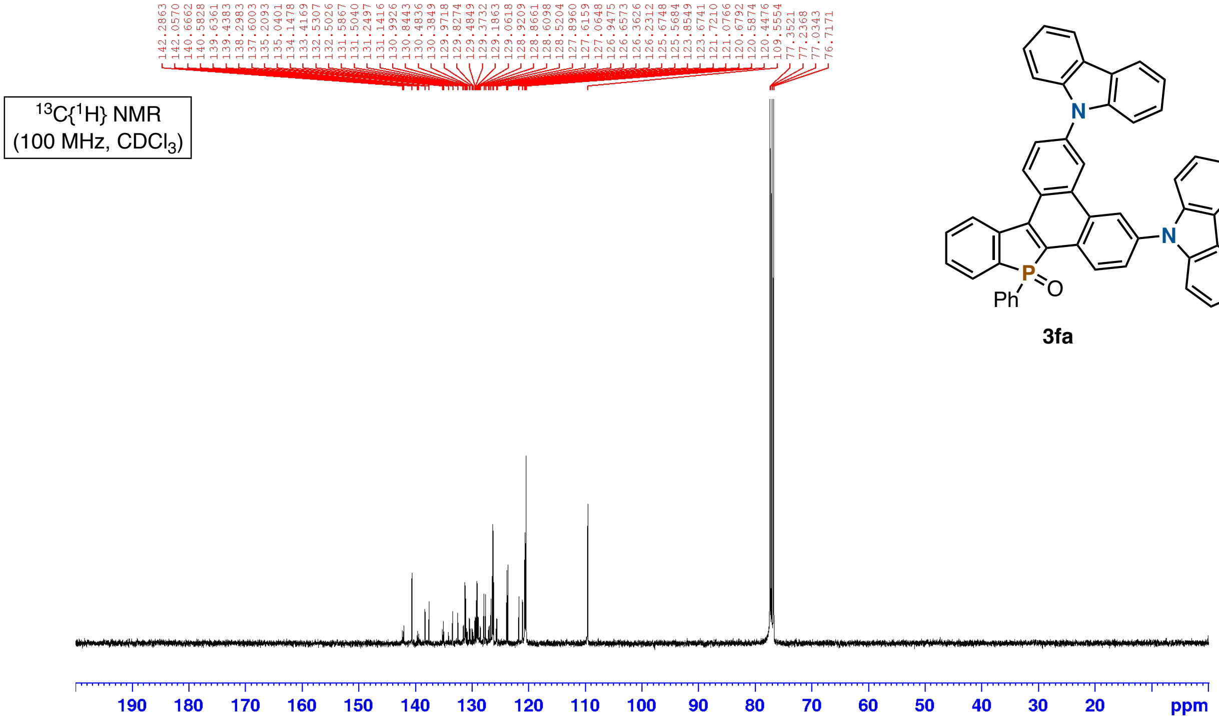

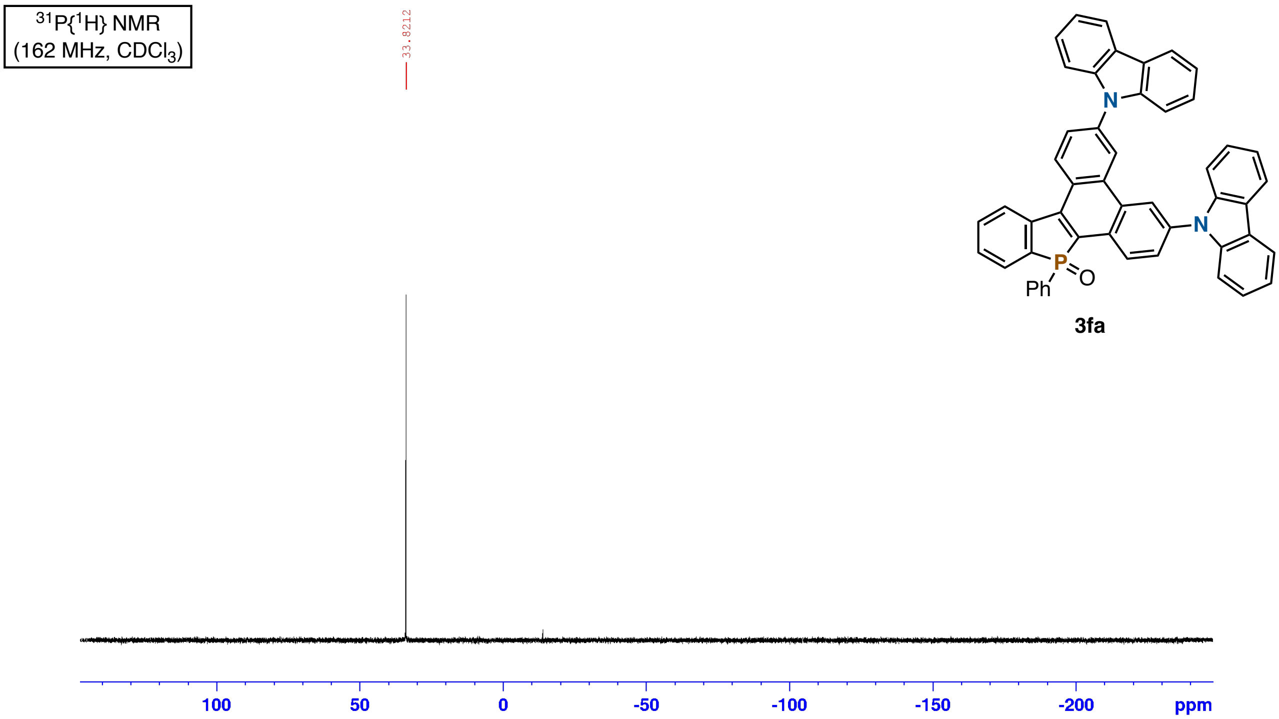

$^{31}P\{^1H\}$ NMR
(162 MHz, $CDCl_3$)
33.8212
100
50
0
-50
-100
-150
-200
ppm
N
N
P
O
Ph

3fa

[^{1}H, ^{13}C{^{1}H}, and ^{31}P{^{1}H} NMR Spectra of **3fb**]

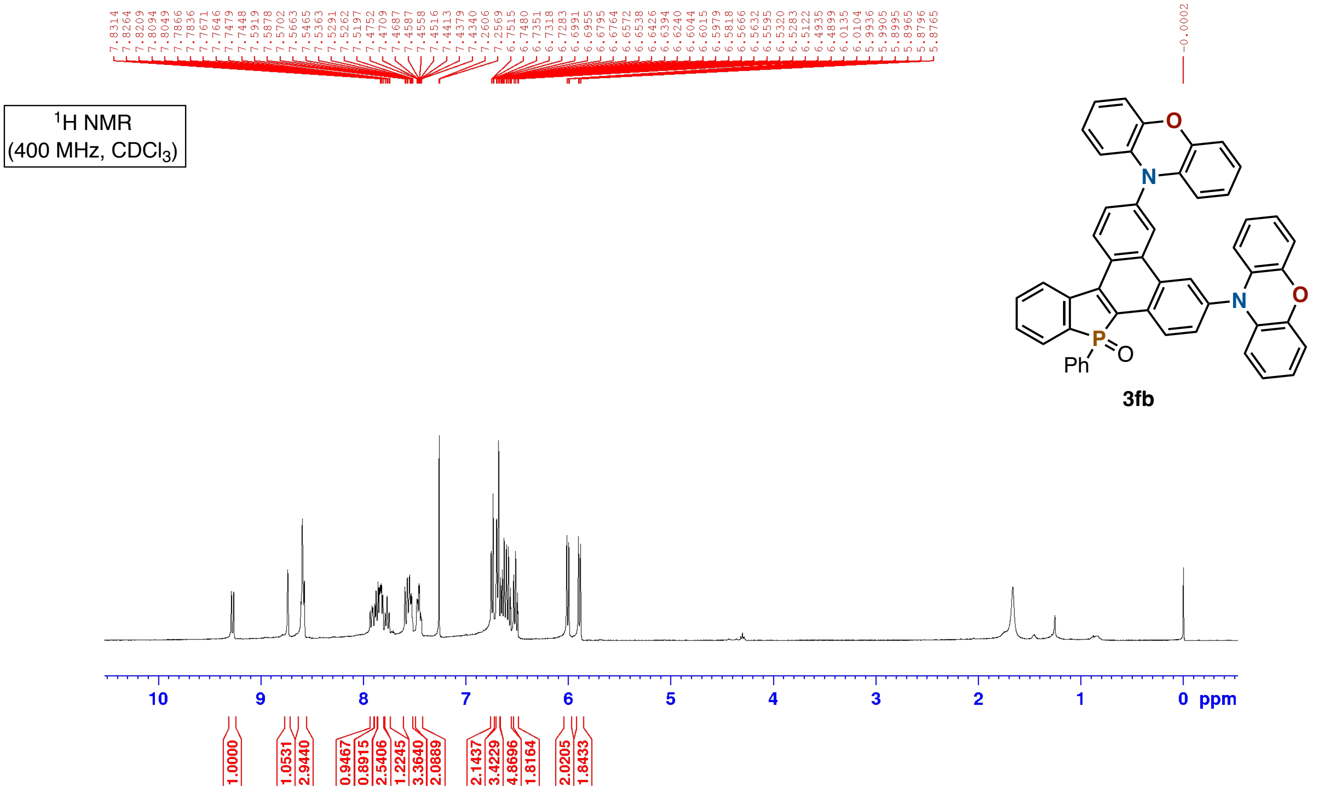

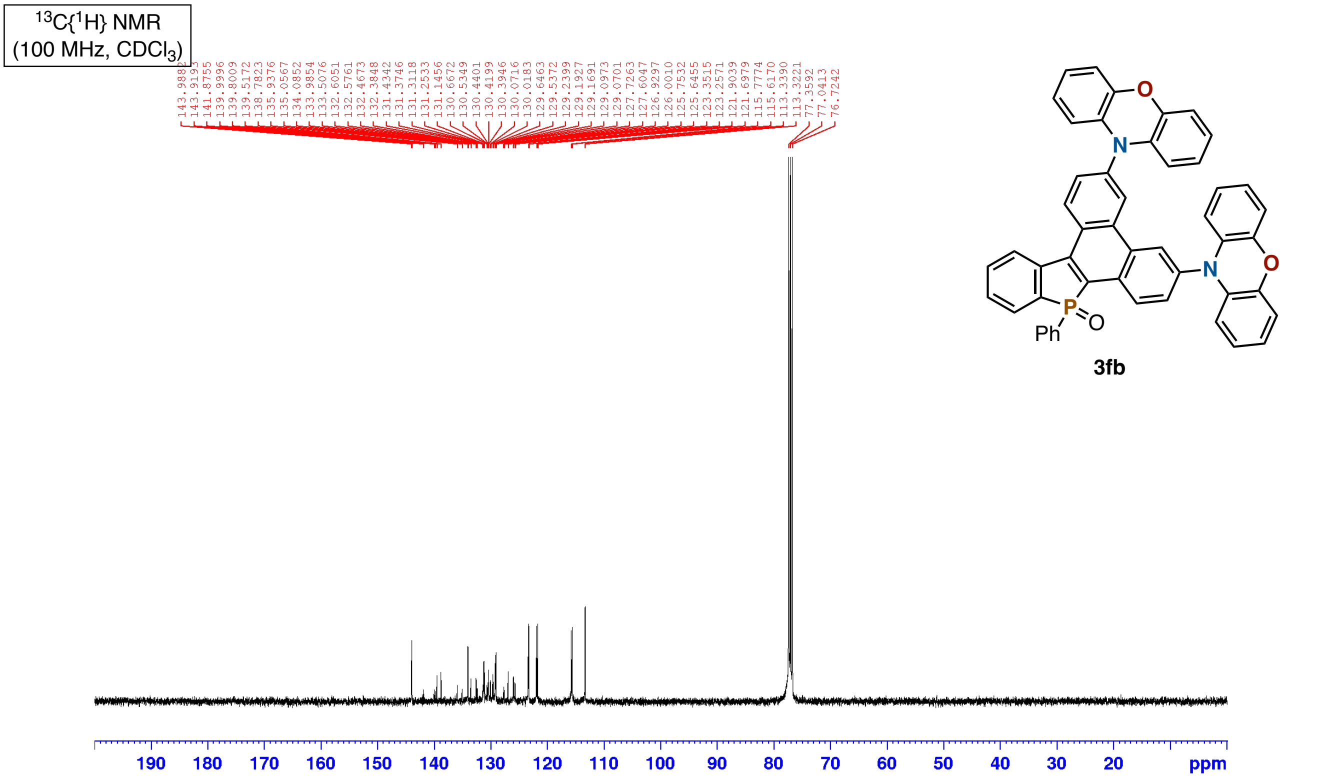

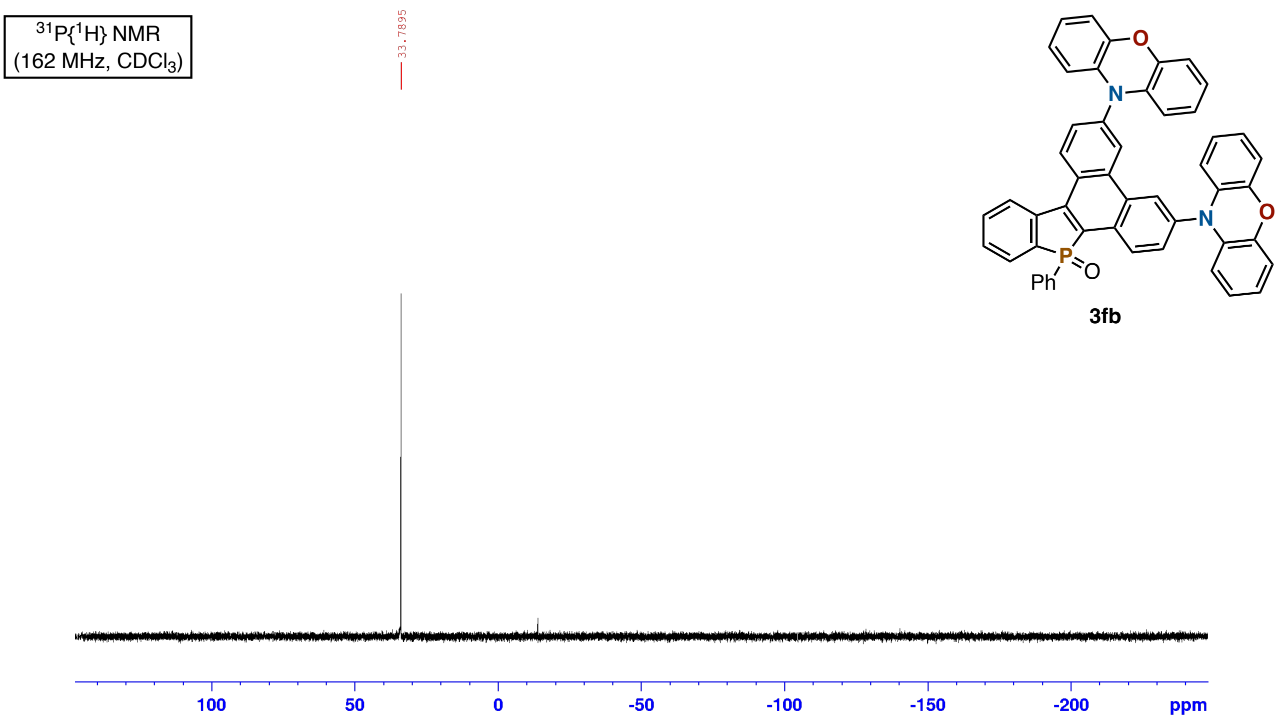

$^{31}P\{^{1}H\}$ NMR
(162 MHz, $CDCl_3$)
33.7895
100
50
0
-50
-100
-150
-200
ppm
O
N
O
N
P
O
Ph

3fb

References

(S1) Y. Unoh, K. Hirano, T. Satoh and M. Miura, *Angew. Chem., Int. Ed.,* 2013, **52**, 12975.

(S2) K. Nishimura, Y. Unoh, K. Hirano and M. Miura, *Chem. Eur. J.,* 2018, **24**, 13089.

(S3) S. Xu, K. Nishimura, K. Saito, K. Hirano and M. Miura, *Chem. Sci.,* 2022, **13**, 10950.

(S4) Y. Tokura, S. Xu, Y. Kojima, M. Miura and K. Hirano, *Chem. Commun*., 2022, **58**, 12208.

(S5) M. J. Frisch, G. W. Trucks, H. B. Schlegel, G. E. Scuseria, M. A. Robb, J. R. Cheeseman, G. Scalmani, V. Barone, G. A. Petersson, H. Nakatsuji, X. Li, M. Caricato, A. V. Marenich, J. Bloino, B. G. Janesko, R. Gomperts, B. Mennucci, H. P. Hratchian, J. V. Ortiz, A. F. Izmaylov, J. L. Sonnenberg, D. Williams-Young, F. Ding, F. Lipparini, F. Egidi, J. Goings, B. Peng, A. Petrone, T. Henderson, D. Ranasinghe, V. G. Zakrzewski, J. Gao, N. Rega, G. Zheng, W. Liang, M. Hada, M. Ehara, K. Toyota, R. Fukuda, J. Hasegawa, M. Ishida, T. Nakajima, Y. Honda, O. Kitao, H. Nakai, T. Vreven, K. Throssell, J. A. Jr., Montgomery, J. E. Peralta, F. Ogliaro, M. J. Bearpark, J. J. Heyd, E. N. Brothers, K. N. Kudin, V. N. Staroverov, T. A. Keith, R. Kobayashi, J. Normand, K. Raghavachari, A. P. Rendell, J. C. Burant, S. S. Iyengar, J. Tomasi, M. Cossi, J. M. Millam, M. Klene, C. Adamo, R. Cammi, J. W. Ochterski, R. L. Martin, K. Morokuma, O. Farkas, J. B. Foresman, D. J. Fox, Gaussian, Inc., Wallingford CT, 2016.

(S6) Y. Zhao and D. G. Truhlar, *Theor. Chem. Acc.,* 2008, **120**, 215.
